# Supplementary material for: New approaches for capturing and estimating variation in complex animal color patterns from digital photographs: application to the Eastern Box Turtle (Terrapene carolina)
Source: PeerJ. 2025 Jul 21;13:e19690. doi: 10.7717/peerj.19690 (PMC12288748; doi:10.7717/peerj.19690)
Supplement: Supplemental Information 1 [file peerj-13-19690-s001.docx]

**Supplemental Material**


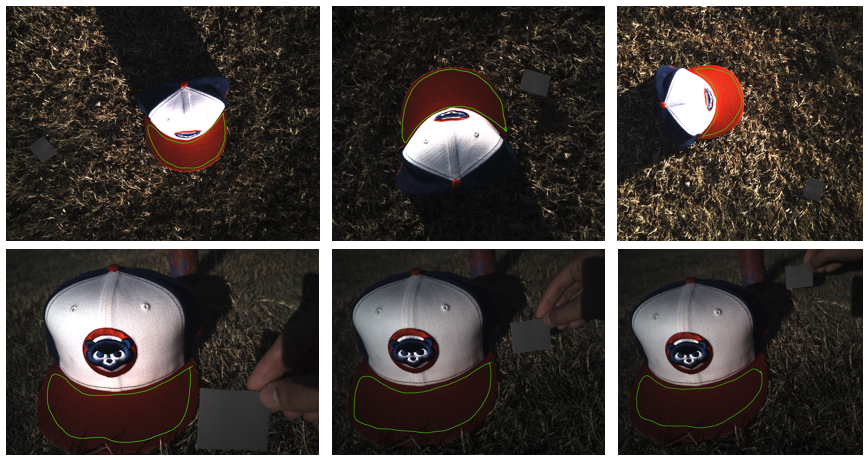


**Figure S1: Testing the image calibration.** We tested how the placement of the 18% color standard impacted the calibration of the image. This simulated the methods of the image process in the field where the standard was placed further from the images for future analysis of the background. Row 1 Coefficient of variation (CV) of the Brightness = 0.4665; Row 2 CV of the Brightness = 0.3785.


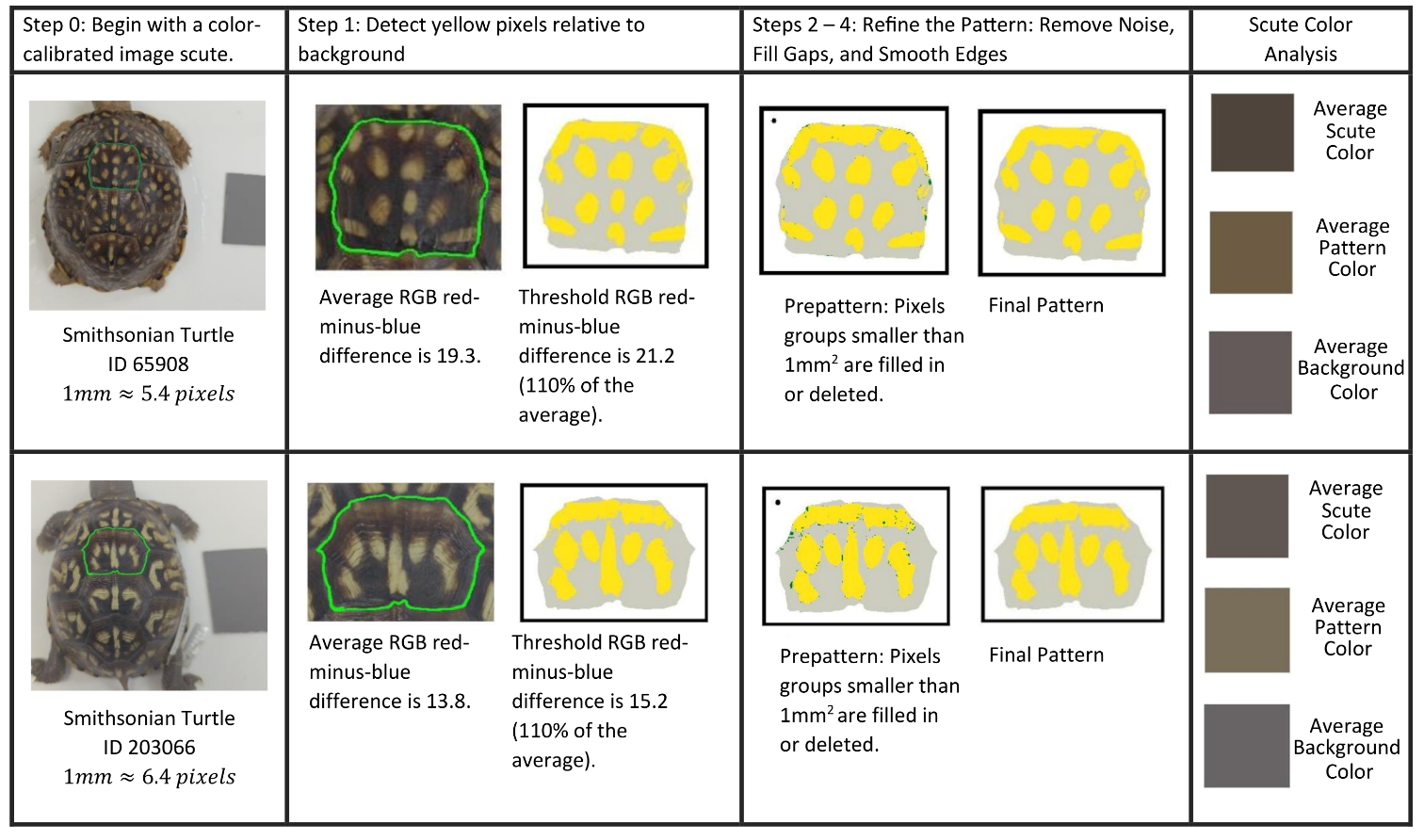


**Figure S2: The steps for pattern extraction shown for two example scutes.**

**Step 0: Begin with a color-calibrated image**. Even if light conditions are different for each image, after calibration the gray card has the same average pixel value photo to photo. The length of the card doubly operates as a 50mm scale bar so that the length scale of the image is known. For each turtle image, a scute in the plane of the view is outlined for segmentation.

**Step 1:** **Determining the Threshold Yellow** For each scute, the average red-minus-blue value across all pixels is computed. A pixel is marked as “yellow” if its red-minus-blue value exceeds 110% of that average. This set of pixels with the threshold red-minus-blue difference establishes a “pre-pattern” that is then refined. The effect of adjusting the threshold value (as *t*% of the average where *t%* varies) on the final pattern is described in Section 3.3 and Figure R6.

**Steps 2—4: Refining to Remove Noise, Fill Gaps, and Smooth Edges** The first scute shows the pre-pattern in yellow and overlaid on top in dark green, are all of the pixels that are added or deleted during refinement of the pattern. Although the number of changed pixels is relatively small, removing such noise has a large effect on the final number of pixel groupings that are considered discrete pattern objects. A disk approximating the 1mm^2^ area for the spatial scale of each scute is added to the image of the prepattern in the upper left-hand corner. The second scute shows the final output of the pattern algorithm, a binary pattern identifying the yellow pattern of the scute.

**Scute Color Analysis:** From the identified yellow pattern, the average RGB pixel values of the scute, the average RGB pixel values of the yellow pattern, and the average RGB pixel values of background pixels are provided to highlight color differences of the groupings.

**Table S1: Color measurement definitions**

| Terms | Definitions |
| --- | --- |
| RGB color space | An additive color model in which the red, green and blue primary colors of light are added together in various ways to reproduce a broad array of colors |
| HSB color space | Uses hue, saturation, and brightness as components for defining color. |
| Luminosity | Is an absolute measure of radiated electromagnetic energy per unit time. |
| Contrast | How much one color stands out from another color. |
| Reflectance | Measures the amount of visible and usable light that reflects from or absorbs into a surface. |
| Hue | Is the measure of the shade of the color. |
| Saturation | is the measure of the intensity of the coloration. |
| Brightness | Is the measure of how the color radiates light. |
| Irradiance | Is the amount of energy emitted at each wavelength of color. |
| Spatial distribution | Is the distribution of the pattern in space. |

**Table S2A**: Coefficient of variation (CV) *across individual turtles* for each measurement per viewpoint is shown in the table. The CV averaged for all views (CV All Views) provides a single CV that captures the variation between individual animals for each measurement. For each view for each of the 55 individual turtles, the average pattern measurement was computed for two images. The mean and standard deviation of these 55 individual turtle measurements were used to calculate the CV for each view (CV=$\frac{\sigma}{\mu}$).

| **Pattern measurement** | **CV Top View** | **CV Right View** | **CV Left View** | **CV Front View** | **CV Back View** | **CV All Views (Averaged)** |
| --- | --- | --- | --- | --- | --- | --- |
| **Fractional Area** | **0.217** | **0.176** | **0.177** | **0.219** | **0.169** | **0.192** |
| **Mean Eccentricity** | **0.079** | **0.074** | **0.058** | **0.078** | **0.099** | **0.078** |
| **Peak Length** | **0.187** | **0.163** | **0.159** | **0.184** | **0.219** | **0.182** |
| **Perimeter/Area** | **0.224** | **0.183** | **0.185** | **0.214** | **0.303** | **0.221** |
| **Hue** | **0.271** | **0.264** | **0.260** | **0.306** | **0.258** | **0.272** |
| **Saturation** | **0.265** | **0.250** | **0.233** | **0.236** | **0.243** | **0.246** |
| **Brightness** | **0.444** | **0.302** | **0.315** | **0.342** | **0.389** | **0.358** |
| **Symmetry** | **0.157** | **0.135** | **0.134** | **0.174** | **0.146** | **0.149** |
| **Euclidean Distance** | **0.625** | **0.452** | **0.454** | **0.436** | **0.515** | **0.496** |
| **Intensity Ratio** | **0.629** | **0.527** | **0.576** | **0.510** | **0.532** | **0.555** |
| **Number of Objects** | **0.514** | **0.598** | **0.583** | **0.508** | **0.798** | **0.600** |
| **Average Area of Objects** | **0.745** | **0.590** | **0.769** | **0.475** | **0.645** | **0.645** |
| **Red Contrast** | **0.630** | **0.468** | **0.502** | **0.441** | **0.504** | **0.509** |
| **Blue Contrast** | **0.729** | **0.972** | **1.109** | **1.374** | **0.740** | **0.985** |
| **Green Contrast** | **0.647** | **0.556** | **0.612** | **0.541** | **0.554** | **0.582** |
| **Yellow Contrast** | **0.746** | **0.877** | **1.004** | **1.125** | **0.699** | **0.890** |
| **Centrality Ratio** | **0.134** | **0.125** | **0.132** | **0.146** | **0.182** | **0.144** |
| **Occupation Factor** | **0.203** | **0.161** | **0.155** | **0.216** | **0.225** | **0.192** |
| **Normalized Offset** | **0.646** | **0.733** | **0.635** | **0.522** | **0.605** | **0.628** |

**Table S2B**: Coefficient of variation (CV) *within individual turtles* (variation between independent images for the same turtle) for each measurement per viewpoint is shown in the table. Pairwise difference divided by the mean (CV) average shows how reliable each measurement is and the variation present between each measurement. The average CV (across all views) is shown for each measure in Figure R9.

| **Pattern measurement** | **CV Top View** | **CV Right View** | **CV Left View** | **CV Front View** | **CV Back View** | **CV All Views (Averaged)** |
| --- | --- | --- | --- | --- | --- | --- |
| **Fractional Area** | **0.031** | **0.021** | **0.025** | **0.038** | **0.037** | **0.031** |
| **Mean Eccentricity** | **0.033** | **0.028** | **0.020** | **0.031** | **0.045** | **0.031** |
| **Peak Length** | **0.052** | **0.035** | **0.032** | **0.058** | **0.073** | **0.050** |
| **Perimeter/Area** | **0.044** | **0.042** | **0.040** | **0.050** | **0.048** | **0.045** |
| **Hue** | **0.046** | **0.025** | **0.027** | **0.034** | **0.029** | **0.032** |
| **Saturation** | **0.028** | **0.011** | **0.011** | **0.018** | **0.014** | **0.016** |
| **Brightness** | **0.079** | **0.041** | **0.031** | **0.060** | **0.046** | **0.051** |
| **Symmetry** | **0.041** | **0.040** | **0.020** | **0.062** | **0.037** | **0.040** |
| **Euclidean Distance** | **0.083** | **0.044** | **0.040** | **0.066** | **0.056** | **0.058** |
| **Intensity Ratio** | **0.082** | **0.050** | **0.043** | **0.105** | **0.059** | **0.068** |
| **Number of Objects** | **0.112** | **0.133** | **0.107** | **0.112** | **0.178** | **0.128** |
| **Average Area of Objects** | **0.934** | **0.132** | **0.241** | **0.180** | **0.192** | **0.336** |
| **Red Contrast** | **0.085** | **0.046** | **0.039** | **0.071** | **0.052** | **0.059** |
| **Blue Contrast** | **0.084** | **0.070** | **0.145** | **0.277** | **0.107** | **0.136** |
| **Green Contrast** | **0.083** | **0.055** | **0.048** | **0.310** | **0.063** | **0.112** |
| **Yellow Contrast** | **0.097** | **0.069** | **0.125** | **0.335** | **0.097** | **0.145** |
| **Centrality Ratio** | **0.017** | **0.011** | **0.010** | **0.021** | **0.025** | **0.017** |
| **Occupation Factor** | **0.040** | **0.025** | **0.023** | **0.046** | **0.056** | **0.038** |
| **Normalized Offset** | **0.168** | **0.110** | **0.129** | **0.133** | **0.141** | **0.136** |

**Table S2C**: Ratio of the CV within turtles and CV across turtles, ordered from greatest to least ratio.

|  | **CV across turtles** | **CV within turtles** | **Ratio** |
| --- | --- | --- | --- |
| **Average Area of Objects** | 0.645 | 0.336 | 0.521 |
| **Mean Eccentricity** | 0.078 | 0.031 | 0.401 |
| **Peak Length** | 0.182 | 0.050 | 0.274 |
| **Symmetry** | 0.149 | 0.040 | 0.267 |
| **Normalized Offset** | 0.628 | 0.136 | 0.217 |
| **Number of Objects** | 0.600 | 0.128 | 0.214 |
| **Perimeter/Area** | 0.221 | 0.045 | 0.202 |
| **Occupation Factor** | 0.192 | 0.038 | 0.198 |
| **Green Contrast** | 0.582 | 0.112 | 0.192 |
| **Yellow Contrast** | 0.890 | 0.145 | 0.163 |
| **Fractional Area** | 0.192 | 0.031 | 0.159 |
| **Brightness** | 0.358 | 0.051 | 0.143 |
| **Blue Contrast** | 0.985 | 0.136 | 0.138 |
| **Intensity Ratio** | 0.555 | 0.068 | 0.123 |
| **Hue** | 0.272 | 0.032 | 0.118 |
| **Centrality Ratio** | 0.144 | 0.017 | 0.116 |
| **Euclidean Distance** | 0.496 | 0.058 | 0.116 |
| **Red Contrast** | 0.509 | 0.059 | 0.115 |
| **Saturation** | 0.246 | 0.016 | 0.066 |

**Table S3:** Measuring the significance of mean, sd and cv of the viewpoints between Smithsonian and field individuals per measure. P-values are used to represent the significance of the difference between the two groups of individuals and were adjusted for 19 independent measures using the Benjamini-Hochberg procedure to control the false discovery rate. A t-test was used to test for significance between the means of the two groups, a f-test was used to test for significance between the sd and cv of the two groups. These tests were computed in Excel for the measurement data for 55 turtles separated as 43 field samples (n=43) and 12 museum samples (n=12). Significant differences of means, sd and cv are indicated in orange, green and indigo, respectively.

| **Pattern Measurement** | **Top View** | **Right View** | **Left View** | **Front View** | **Back View** |
| --- | --- | --- | --- | --- | --- |
| **Fractional Area** |  |  |  |  |  |
| **Museum Vs. Field T-Test, p-value** | T(42,11)=-0.07, p=0.99 | T(42,11)=0.72, p=0.60 | T(42,11)=-0.14, p=0.91 | T(42,11)=-0.30, p=1.00 | T(42,11)=0.72, p=0.69 |
| **Museum Vs. Field F-Test, p-value** | T(42,11)=2.19, p=0.27 | T(42,11)=1.29, p=0.63 | T(42,11)=1.12, p=0.87 | T(42,11)=2.42, p=0.15 | T(42,11)=1.31, p=0.73 |
| **Museum Vs. Field CV diff., p-value** | T(42,11)=2.63, p=0.02 | T(42,11)=2.26, p=0.45 | T(42,11)=3.02, p=0.09 | T(42,11)=1.98, p=0.10 | T(42,11)=1.77, p=0.12 |
| **Mean Eccentricity** |  |  |  |  |  |
| **Museum Vs. Field T-Test, p-value** | T(42,11)=-0.41, p=0.96 | T(42,11)=1.28, p=0.44 | T(42,11)=1.30, p=0.47 | T(42,11)=-1.07, p=0.81 | T(42,11)=0.56, p=0.69 |
| **Museum Vs. Field F-Test, p-value** | T(42,11)=1.55, p=0.49 | T(42,11)=1.81, p=0.32 | T(42,11)=1.34, p=0.72 | T(42,11)=2.26, p=0.30 | T(42,11)=1.27, p=0.73 |
| **Museum Vs. Field CV diff., p-value** | T(42,11)=7.71, p<0.01 | T(42,11)=2.11, p=0.47 | T(42,11)=2.24, p=0.23 | T(42,11)=1.55, p=0.42 | T(42,11)=1.54, p=0.56 |
| **Peak Length** |  |  |  |  |  |
| **Museum Vs. Field T-Test, p-value** | T(42,11)=2.85, p=0.03 | T(42,11)=-2.43, p=0.15 | T(42,11)=-1.72, p=0.31 | T(42,11)=-0.20, p=1.00 | T(42,11)=-1.27, p=0.63 |
| **Museum Vs. Field F-Test, p-value** | T(42,11)=1.83, p=0.37 | T(42,11)=4.78, p<0.01 | T(42,11)=2.05, p=0.20 | T(42,11)=2.24, p=0.15 | T(42,11)=2.42, p=0.10 |
| **Museum Vs. Field CV diff., p-value** | T(42,11)=15.87, p<0.01 | T(42,11)=1.80, p=0.33 | T(42,11)=1.87, p=0.23 | T(42,11)=1.12, p=0.66 | T(42,11)=5.01, p=0.56 |
| **Perimeter Divided Area** |  |  |  |  |  |
| **Museum Vs. Field T-Test, p-value** | T(42,11)=-0.70, p=0.75 | T(42,11)=2.80, p=0.15 | T(42,11)=2.12, p=0.31 | T(42,11)=0.70, p=0.81 | T(42,11)=1.54, p=0.56 |
| **Museum Vs. Field F-Test, p-value** | T(42,11)=3.21, p=0.09 | T(42,11)=1.39, p=0.63 | T(42,11)=1.06, p=0.99 | T(42,11)=1.14, p=0.80 | T(42,11)=1.01, p=0.90 |
| **Museum Vs. Field CV diff., p-value** | T(42,11)=10.24, p<0.01 | T(42,11)=2.55, p=0.71 | T(42,11)=1.16, p=0.83 | T(42,11)=14.12, p<0.01 | T(42,11)=1.33, p=0.99 |
| **Hue** |  |  |  |  |  |
| **Museum Vs. Field T-Test, p-value** | T(42,11)=-3.17, p=0.03 | T(42,11)=-2.64, p=0.15 | T(42,11)=-2.68, p=0.31 | T(42,11)=-2.61, p=0.40 | T(42,11)=-1.92, p=0.50 |
| **Museum Vs. Field F-Test, p-value** | T(42,11)=1.02, p=0.94 | T(42,11)=10.03, p<0.01 | T(42,11)=11.37, p<0.01 | T(42,11)=10.54, p<0.01 | T(42,11)=9.50, p<0.01 |
| **Museum Vs. Field CV diff., p-value** | T(42,11)=13.76, p<0.01 | T(42,11)=3.56, p=0.09 | T(42,11)=1.95, p=0.03 | T(42,11)=11.12, p<0.01 | T(42,11)=4.24, p=0.20 |
| **Saturation** |  |  |  |  |  |
| **Museum Vs. Field T-Test, p-value** | T(42,11)=-0.21, p=0.97 | T(42,11)=1.47, p=0.44 | T(42,11)=1.79, p=0.31 | T(42,11)=1.02, p=0.81 | T(42,11)=0.62, p=0.69 |
| **Museum Vs. Field F-Test, p-value** | T(42,11)=1.51, p=0.41 | T(42,11)=2.29, p=0.13 | T(42,11)=2.20, p=0.20 | T(42,11)=6.62, p<0.01 | T(42,11)=3.21, p=0.04 |
| **Museum Vs. Field CV diff., p-value** | T(42,11)=2.78, p=0.53 | T(42,11)=9.65, p<0.01 | T(42,11)=1.79, p=0.70 | T(42,11)=3.47, p=0.06 | T(42,11)=3.19, p=0.44 |
| **Brightness** |  |  |  |  |  |
| **Museum Vs. Field T-Test, p-value** | T(42,11)=-3.46, p<0.01 | T(42,11)=1.33, p=0.44 | T(42,11)=1.46, p=0.43 | T(42,11)=1.07, p=0.81 | T(42,11)=2.34, p=0.40 |
| **Museum Vs. Field F-Test, p-value** | T(42,11)=5.60, p<0.01 | T(42,11)=3.36, p<0.01 | T(42,11)=3.49, p<0.01 | T(42,11)=1.37, p=0.69 | T(42,11)=2.94, p=0.04 |
| **Museum Vs. Field CV diff., p-value** | T(42,11)=32.11, p<0.01 | T(42,11)=2.28, p=0.20 | T(42,11)=1.66, p=0.03 | T(42,11)=1.20, p=0.80 | T(42,11)=13.22, p=0.07 |
| **Symmetry** |  |  |  |  |  |
| **Museum Vs. Field T-Test, p-value** | T(42,11)=1.64, p=0.20 | T(42,11)=0.61, p=0.63 | T(42,11)=-0.58, p=0.67 | T(42,11)=1.25, p=0.81 | T(42,11)=-0.87, p=0.69 |
| **Museum Vs. Field F-Test, p-value** | T(42,11)=1.03, p=0.97 | T(42,11)=1.26, p=0.63 | T(42,11)=1.01, p=0.98 | T(42,11)=1.44, p=0.65 | T(42,11)=1.84, p=0.45 |
| **Museum Vs. Field CV diff., p-value** | T(42,11)=3.12, p=0.83 | T(42,11)=1.08, p=0.07 | T(42,11)=2.03, p=0.47 | T(42,11)=3.41, p=0.84 | T(42,11)=4.50, p=0.99 |
| **Euclidean Distance** |  |  |  |  |  |
| **Museum Vs. Field T-Test, p-value** | T(42,11)=-4.28, p<0.01 | T(42,11)=-1.32, p=0.44 | T(42,11)=-0.63, p=0.67 | T(42,11)=-1.04, p=0.81 | T(42,11)=0.60, p=0.69 |
| **Museum Vs. Field F-Test, p-value** | T(42,11)=2.97, p=0.03 | T(42,11)=2.37, p=0.11 | T(42,11)=2.95, p=0.05 | T(42,11)=2.28, p=0.15 | T(42,11)=2.52, p=0.10 |
| **Museum Vs. Field CV diff., p-value** | T(42,11)=28.96, p<0.01 | T(42,11)=2.26, p=0.73 | T(42,11)=1.16, p<0.01 | T(42,11)=1.15, p=0.27 | T(42,11)=10.58, p=0.99 |
| **Intensity Contrast** |  |  |  |  |  |
| **Museum Vs. Field T-Test, p-value** | T(42,11)=-4.30, p<0.01 | T(42,11)=-0.90, p=0.55 | T(42,11)=-0.87, p=0.59 | T(42,11)=-0.91, p=0.81 | T(42,11)=0.70, p=0.69 |
| **Museum Vs. Field F-Test, p-value** | T(42,11)=2.60, p=0.08 | T(42,11)=2.63, p=0.07 | T(42,11)=1.61, p=0.43 | T(42,11)=1.98, p=0.24 | T(42,11)=2.27, p=0.13 |
| **Museum Vs. Field CV diff., p-value** | T(42,11)=30.94, p<0.01 | T(42,11)=2.16, p=0.42 | T(42,11)=1.35, p=0.13 | T(42,11)=1.15, p=0.26 | T(42,11)=11.17, p=0.88 |
| **Num. of Objects** |  |  |  |  |  |
| **Museum Vs. Field T-Test, p-value** | T(42,11)=-0.17, p=0.97 | T(42,11)=-0.87, p=0.55 | T(42,11)=-0.90, p=0.59 | T(42,11)=-0.59, p=0.81 | T(42,11)=1.67, p=0.50 |
| **Museum Vs. Field F-Test, p-value** | T(42,11)=2.11, p=0.28 | T(42,11)=1.62, p=0.52 | T(42,11)=1.15, p=0.87 | T(42,11)=1.47, p=0.51 | T(42,11)=7.02, p<0.01 |
| **Museum Vs. Field CV diff., p-value** | T(42,11)=5.25, p<0.01 | T(42,11)=1.82, p=0.94 | T(42,11)=1.37, p=0.23 | T(42,11)=1.30, p=0.66 | T(42,11)=2.68, p=0.30 |
| **Average Area of Objects** |  |  |  |  |  |
| **Museum Vs. Field T-Test, p-value** | T(42,11)=1.91, p=0.12 | T(42,11)=-1.38, p=0.44 | T(42,11)=0.12, p=0.91 | T(42,11)<0.01, p=1.00 | T(42,11)=0.25, p=0.85 |
| **Museum Vs. Field F-Test, p-value** | T(42,11)=5.10, p=0.03 | T(42,11)=1.96, p=0.42 | T(42,11)=3.62, p=0.12 | T(42,11)=2.07, p=0.33 | T(42,11)=1.47, p=0.73 |
| **Museum Vs. Field CV diff., p-value** | T(42,11)=24.11, p<0.01 | T(42,11)=2.97, p=0.94 | T(42,11)=4.43, p=0.85 | T(42,11)=4.51, p=0.06 | T(42,11)=4.77, p=0.38 |
| **Red Contrast** |  |  |  |  |  |
| **Museum Vs. Field T-Test, p-value** | T(42,11)=-4.17, p<0.01 | T(42,11)=-1.05, p=0.52 | T(42,11)=-0.90, p=0.59 | T(42,11)=-1.27, p=0.81 | T(42,11)=0.44, p=0.74 |
| **Museum Vs. Field F-Test, p-value** | T(42,11)=3.80, p<0.01 | T(42,11)=3.96, p<0.01 | T(42,11)=2.84, p=0.05 | T(42,11)=2.66, p=0.10 | T(42,11)=3.13, p=0.04 |
| **Museum Vs. Field CV diff., p-value** | T(42,11)=30.23, p<0.01 | T(42,11)=2.56, p=0.94 | T(42,11)=1.10, p<0.01 | T(42,11)=1.14, p=0.78 | T(42,11)=9.50, p=0.99 |
| **Peak Length** |  |  |  |  |  |
| **Museum Vs. Field T-Test, p-value** | T(42,11)=2.85, p=0.03 | T(42,11)=-2.43, p=0.15 | T(42,11)=-1.72, p=0.31 | T(42,11)=-0.20, p=1.00 | T(42,11)=-1.27, p=0.63 |
| **Museum Vs. Field F-Test, p-value** | T(42,11)=1.83, p=0.37 | T(42,11)=4.78, p<0.01 | T(42,11)=2.05, p=0.20 | T(42,11)=2.24, p=0.15 | T(42,11)=2.42, p=0.10 |
| **Museum Vs. Field CV diff., p-value** | T(42,11)=15.87, p<0.01 | T(42,11)=1.80, p=0.33 | T(42,11)=1.87, p=0.23 | T(42,11)=1.12, p=0.66 | T(42,11)=5.01, p=0.56 |
| **Blue Contrast** |  |  |  |  |  |
| **Museum Vs. Field T-Test, p-value** | T(42,11)=-1.71, p=0.20 | T(42,11)=-0.33, p=0.75 | T(42,11)=-0.71, p=0.65 | T(42,11)=0.03, p=1.00 | T(42,11)=1.17, p=0.65 |
| **Museum Vs. Field F-Test, p-value** | T(42,11)=3.38, p<0.01 | T(42,11)=1.23, p=0.63 | T(42,11)=2.25, p=0.27 | T(42,11)=1.21, p=0.81 | T(42,11)=1.11, p=0.84 |
| **Museum Vs. Field CV diff., p-value** | T(42,11)=8.87, p=0.02 | T(42,11)=2.73, p=0.09 | T(42,11)=2.06, p<0.01 | T(42,11)=2.37, p=0.06 | T(42,11)=3.74, p=0.23 |
| **Green Contrast** |  |  |  |  |  |
| **Museum Vs. Field T-Test, p-value** | T(42,11)=-4.31, p<0.01 | T(42,11)=-0.85, p=0.55 | T(42,11)=-0.85, p=0.59 | T(42,11)=-0.75, p=0.81 | T(42,11)=0.83, p=0.69 |
| **Museum Vs. Field F-Test, p-value** | T(42,11)=2.28, p=0.10 | T(42,11)=2.22, p=0.13 | T(42,11)=1.27, p=0.75 | T(42,11)=1.74, p=0.33 | T(42,11)=1.90, p=0.25 |
| **Museum Vs. Field CV diff., p-value** | T(42,11)=33.41, p<0.01 | T(42,11)=1.81, p=0.08 | T(42,11)=1.83, p=0.51 | T(42,11)=1.23, p=0.20 | T(42,11)=13.19, p<0.01 |
| **Yellow Contrast** |  |  |  |  |  |
| **Museum Vs. Field T-Test, p-value** | T(42,11)=-2.42, p=0.07 | T(42,11)=-1.47, p=0.44 | T(42,11)=-1.91, p=0.31 | T(42,11)=-1.50, p=0.81 | T(42,11)=0.75, p=0.69 |
| **Museum Vs. Field F-Test, p-value** | T(42,11)=4.24, p<0.01 | T(42,11)=1.02, p=0.90 | T(42,11)=2.66, p=0.20 | T(42,11)=1.18, p=0.81 | T(42,11)=1.08, p=0.84 |
| **Museum Vs. Field CV diff., p-value** | T(42,11)=45.00, p<0.01 | T(42,11)=1.04, p=0.08 | T(42,11)=1.55, p=0.60 | T(42,11)=3.59, p=0.38 | T(42,11)=3.33, p=0.33 |
| **Centrality Ratio** |  |  |  |  |  |
| **Museum Vs. Field T-Test, p-value** | T(42,11)=0.19, p=0.97 | T(42,11)=1.18, p=0.45 | T(42,11)=1.15, p=0.52 | T(42,11)=-0.77, p=0.81 | T(42,11)=0.87, p=0.69 |
| **Museum Vs. Field F-Test, p-value** | T(42,11)=5.89, p<0.01 | T(42,11)=1.87, p=0.42 | T(42,11)=2.30, p=0.27 | T(42,11)=1.67, p=0.51 | T(42,11)=1.10, p=0.84 |
| **Museum Vs. Field CV diff., p-value** | T(42,11)=7.00, p<0.01 | T(42,11)=1.05, p=0.94 | T(42,11)=2.96, p=0.13 | T(42,11)=3.45, p=0.06 | T(42,11)=34.43, p<0.01 |
| **Occupation Factor** |  |  |  |  |  |
| **Museum Vs. Field T-Test, p-value** | T(42,11)=0.02, p=0.99 | T(42,11)=0.59, p=0.63 | T(42,11)=-0.35, p=0.81 | T(42,11)=-0.06, p=1.00 | T(42,11)=0.14, p=0.94 |
| **Museum Vs. Field F-Test, p-value** | T(42,11)=2.38, p=0.22 | T(42,11)=1.60, p=0.42 | T(42,11)=1.26, p=0.75 | T(42,11)=1.36, p=0.61 | T(42,11)=1.18, p=0.84 |
| **Museum Vs. Field CV diff., p-value** | T(42,11)=2.95, p<0.01 | T(42,11)=1.07, p=0.22 | T(42,11)=4.00, p<0.01 | T(42,11)=3.71, p<0.01 | T(42,11)=6.89, p=0.99 |
| **Normalized Offset** |  |  |  |  |  |
| **Museum Vs. Field T-Test, p-value** | T(42,11)=-0.37, p=0.96 | T(42,11)=0.42, p=0.72 | T(42,11)=1.85, p=0.31 | T(42,11)=0.59, p=0.81 | T(42,11)=2.76, p=0.20 |
| **Museum Vs. Field F-Test, p-value** | T(42,11)=1.68, p=0.42 | T(42,11)=1.49, p=0.49 | T(42,11)=1.00, p=0.98 | T(42,11)=4.57, p<0.01 | T(42,11)=2.30, p=0.25 |
| **Museum Vs. Field CV diff., p-value** | T(42,11)=9.04, p=0.02 | T(42,11)=1.46, p=0.07 | T(42,11)=1.54, p=0.59 | T(42,11)=6.02, p=0.78 | T(42,11)=4.74, p=0.12 |

**Table S4**: Coefficient of variation (CV) of 7 measurements per view across individuals and across all viewpoints as the angle of photography was varied. Each viewpoint had three pictures taken per view point per angle: top (-10°, -5°, 0°, 5°, 10°) and front, back, left, and right (0°, 5°, 10°). For each view, angle CV was computed for three turtles, with three pictures per turtle, for either 3 or 5 angles, and thus computed from 27 (=3x3x3) or 45 (=3x3x5) data points. The four pattern measurements with highest variation (CV averaged for all views) are highlighted in red and the three with the lowest variation (lowest CV averaged for all views) are highlighted in blue. The within individual turtle variation across all views in the absence of angle variation is shown in the last column for comparison (“Comparison Across Views”). Figure R10 shows the average CV (across views) versus each measure.

| **Pattern measurement** | **Angle CV,**  **Top View** | **Angle CV, Front View** | **Angle CV, Back View** | **Angle CV, Left View** | **Angle CV, Right View** | **Angle CV, Across Views** | **Baseline CV,**  **ComparisonAcross Views** |
| --- | --- | --- | --- | --- | --- | --- | --- |
| **Mean Eccentricity** | 0.0369 | 0.0494 | 0.0376 | 0.0504 | 0.0335 | 0.042 | 0.031 |
| **Euclidean Distance** | 0.0780 | 0.2517 | 0.3109 | 0.3811 | 0.3549 | 0.275 | 0.058 |
| **Intensity Ratio** | 0.076 | 0.2551 | 0.3114 | 0.7096 | 0.3103 | 0.332 | 0.068 |
| **Average Area of Objects** | 0.1027 | 0.4284 | 0.2948 | 0.2948 | 0.4013 | 0.304 | 0.336 |
| **Centrality Ratio** | 0.0091 | 0.019 | 0.0125 | 0.026 | 0.054 | 0.024 | 0.017 |
| **Occupation Factor** | 0.0144 | 0.0497 | 0.0645 | 0.0355 | 0.0371 | 0.040 | 0.038 |
| **Normalized Offset** | 0.1287 | 0.4575 | 0.2709 | 0.4751 | 0.676 | 0.402 | 0.136 |

**Table S5**: Summary of best practices for capturing and analyzing complex animal color patterns from digital photographs

| Digital Photography | For slow-moving animal specimens that can be closely approached:   - Take photos of animals with a calibration card (e.g., an 18% gray card) that provides useful information for animal scale, photo color calibration and photo exposure calibration. - Place the photo card in the same light as the animal. - Take photos of the animal in bright, uniform lighting if possible. - With the use of a calibration card, photos can be taken from different distances or with different lighting conditions. Try to minimize the variation of the angle of viewing between photos. - Take photos from several views since this increases the probability that a located feature of interest will be within the plane of view without much distortion. - To measure error of the methodology, take two or more independent photos from each view. |
| --- | --- |
| Choice of Animal Specimens | Additional points to be aware of:   - Use photos of the animal without extensive shadows or dappled light since this adds noise to color information. (E.g. see the photo of the turtle with ID mn01 in Figure 3 with dappled lighting.) - Use photos of animals with more vibrant colors since this enhances pattern-background contrast and improves the algorithm's reliability in detecting pattern objects. - If relevant and practical, dry or clean the animal of dust and debris. |
| Pattern Measures | - For complex patterns, focus on pattern measures that do not depend on the number of objects or the area of individual objects, since the separation of objects is unpredictable for complex patterns (see Figure 2). - Scale invariant measures are especially useful for comparison across animals of different ages (stages of development) and/or sizes. - The variation of pattern measures for two or more independent photos provides a quantitative measure of the methodology error. |
