# Supplementary figures and images for: New approaches for capturing and estimating variation in complex animal color patterns from digital photographs: application to the Eastern Box Turtle (Terrapene carolina)
Source: PeerJ. 2025 Jul 21;13:e19690. doi: 10.7717/peerj.19690 (PMC12288748; doi:10.7717/peerj.19690)

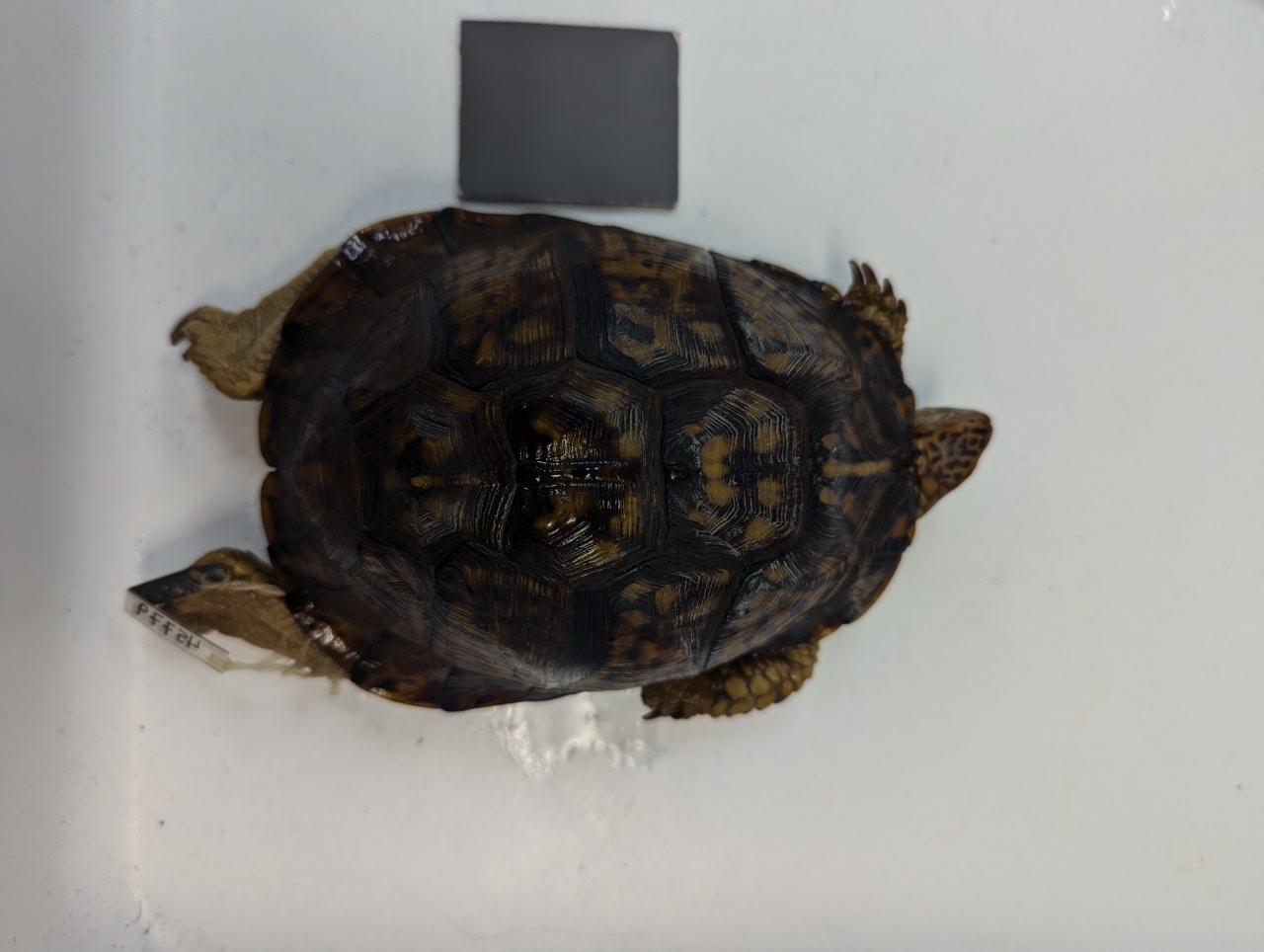

Supplement: Supplemental Information 6 — 98 photos of 98 turtles (single photo, all top view) that were used for the Citizen Science classification analysis. [file peerj-13-19690-s006.zip › TurtleClassification/TURTLE1001.jpg]

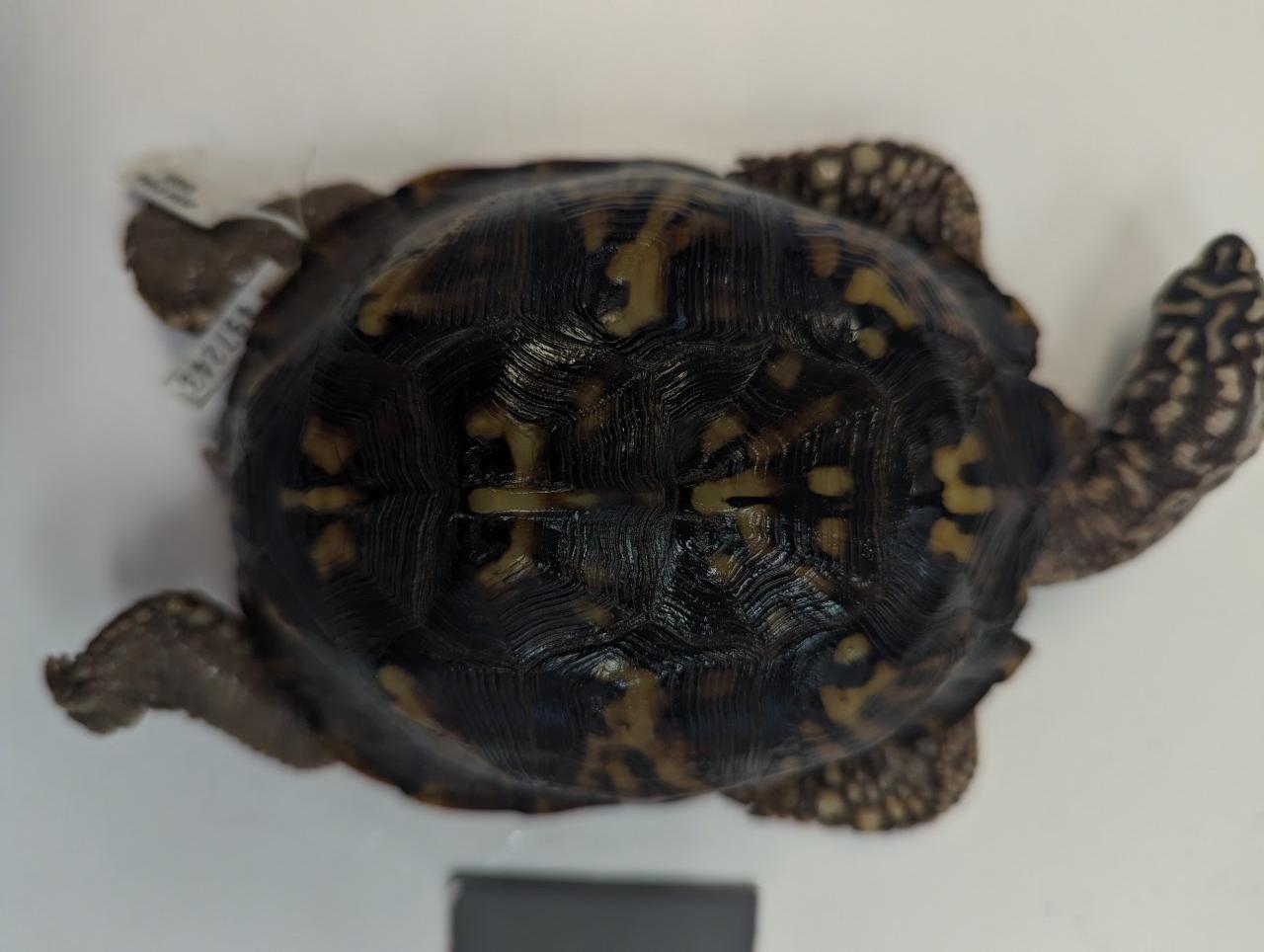

Supplement: Supplemental Information 6 — 98 photos of 98 turtles (single photo, all top view) that were used for the Citizen Science classification analysis. [file peerj-13-19690-s006.zip › TurtleClassification/TURTLE1002.jpg]

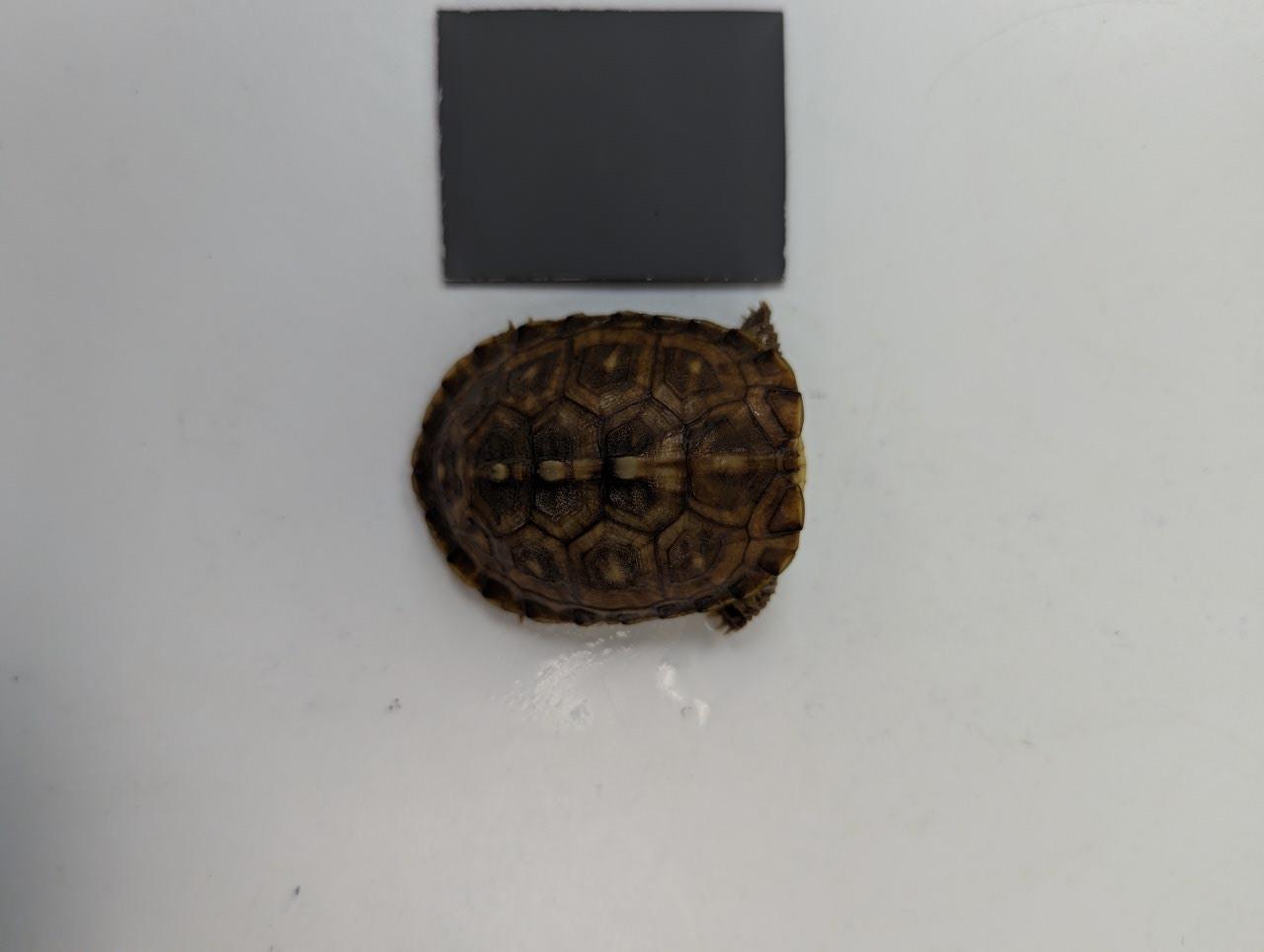

Supplement: Supplemental Information 6 — 98 photos of 98 turtles (single photo, all top view) that were used for the Citizen Science classification analysis. [file peerj-13-19690-s006.zip › TurtleClassification/TURTLE1003.jpg]

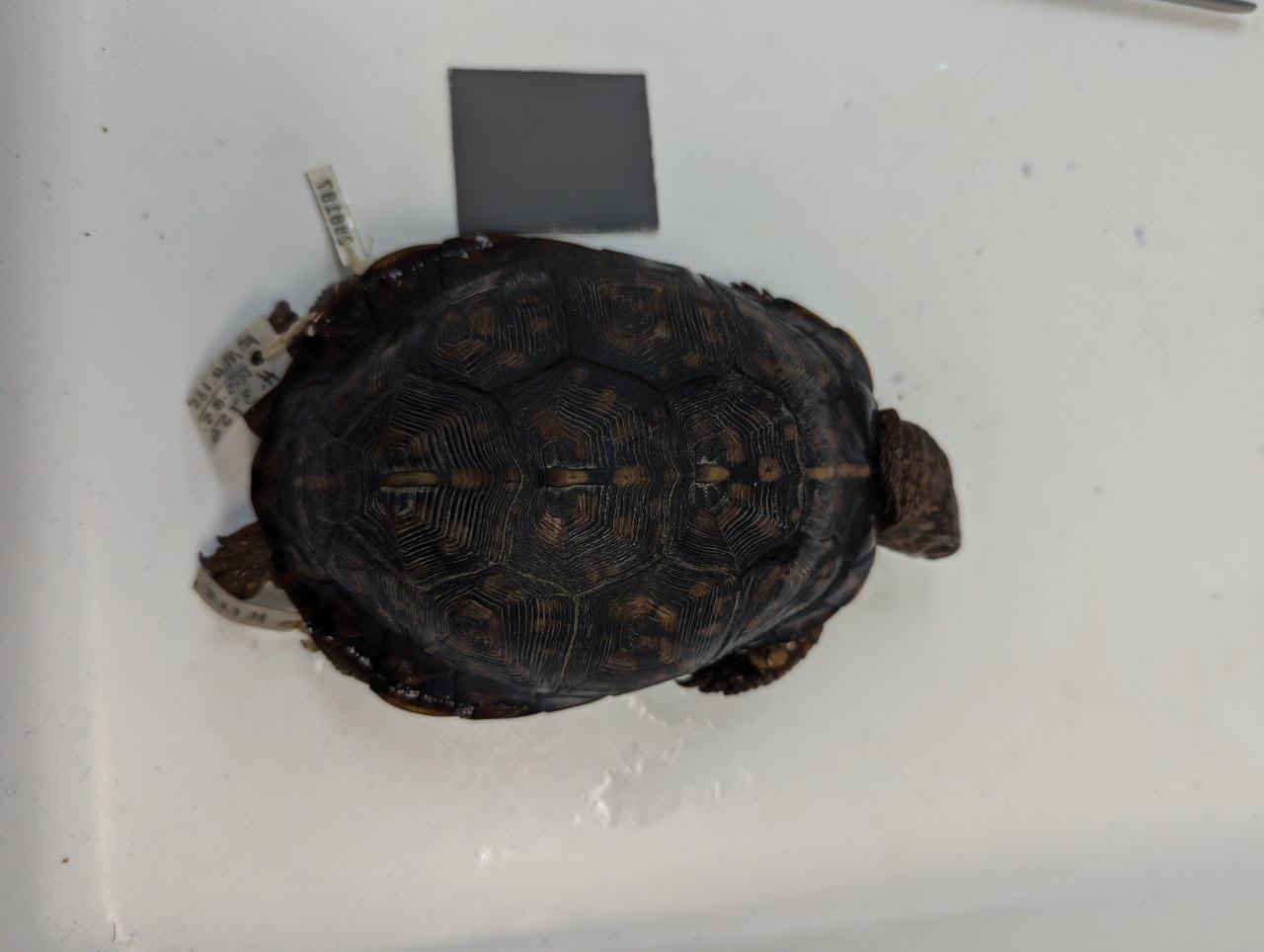

Supplement: Supplemental Information 6 — 98 photos of 98 turtles (single photo, all top view) that were used for the Citizen Science classification analysis. [file peerj-13-19690-s006.zip › TurtleClassification/TURTLE1004.jpg]

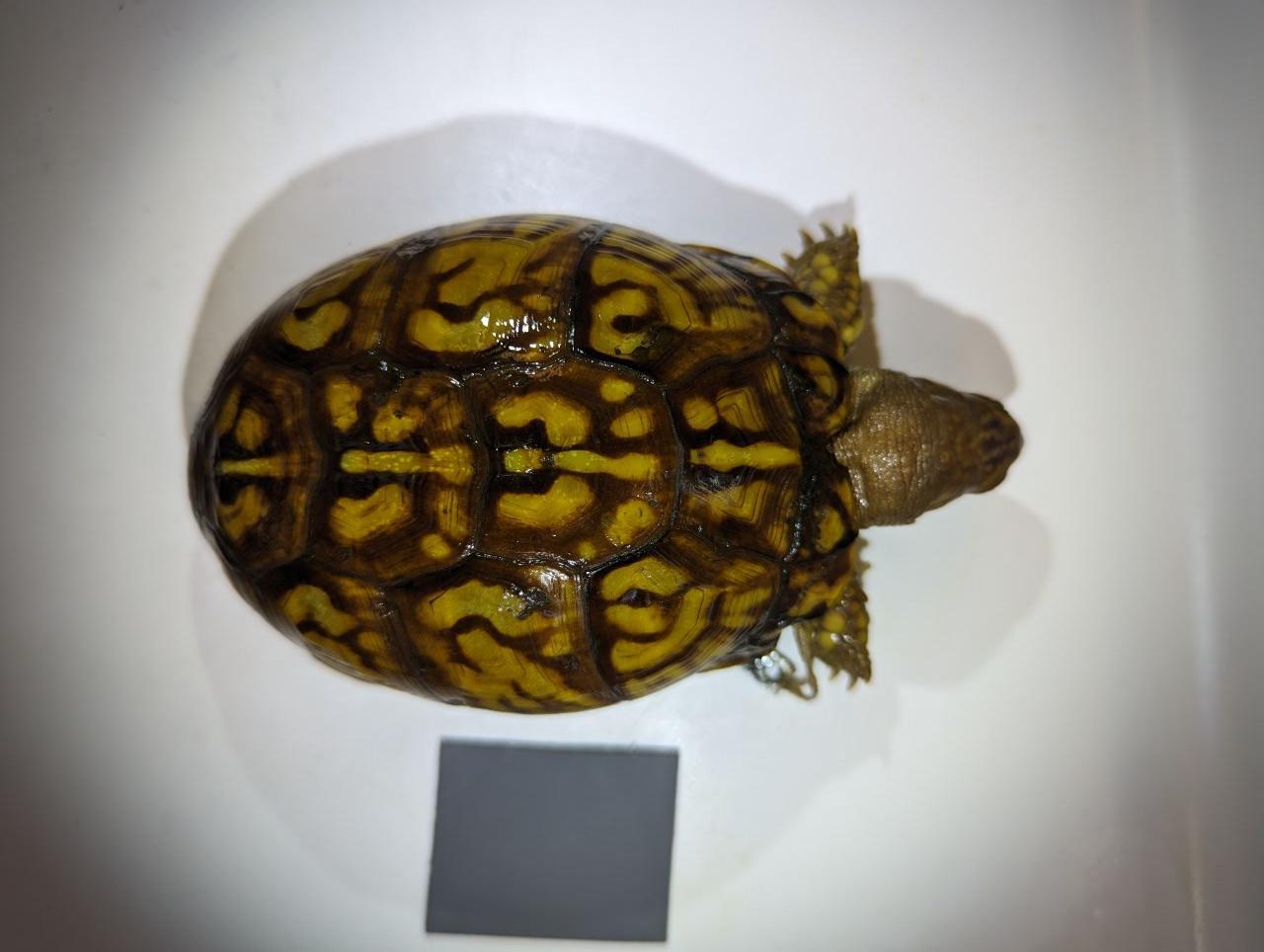

Supplement: Supplemental Information 6 — 98 photos of 98 turtles (single photo, all top view) that were used for the Citizen Science classification analysis. [file peerj-13-19690-s006.zip › TurtleClassification/TURTLE1005.jpg]

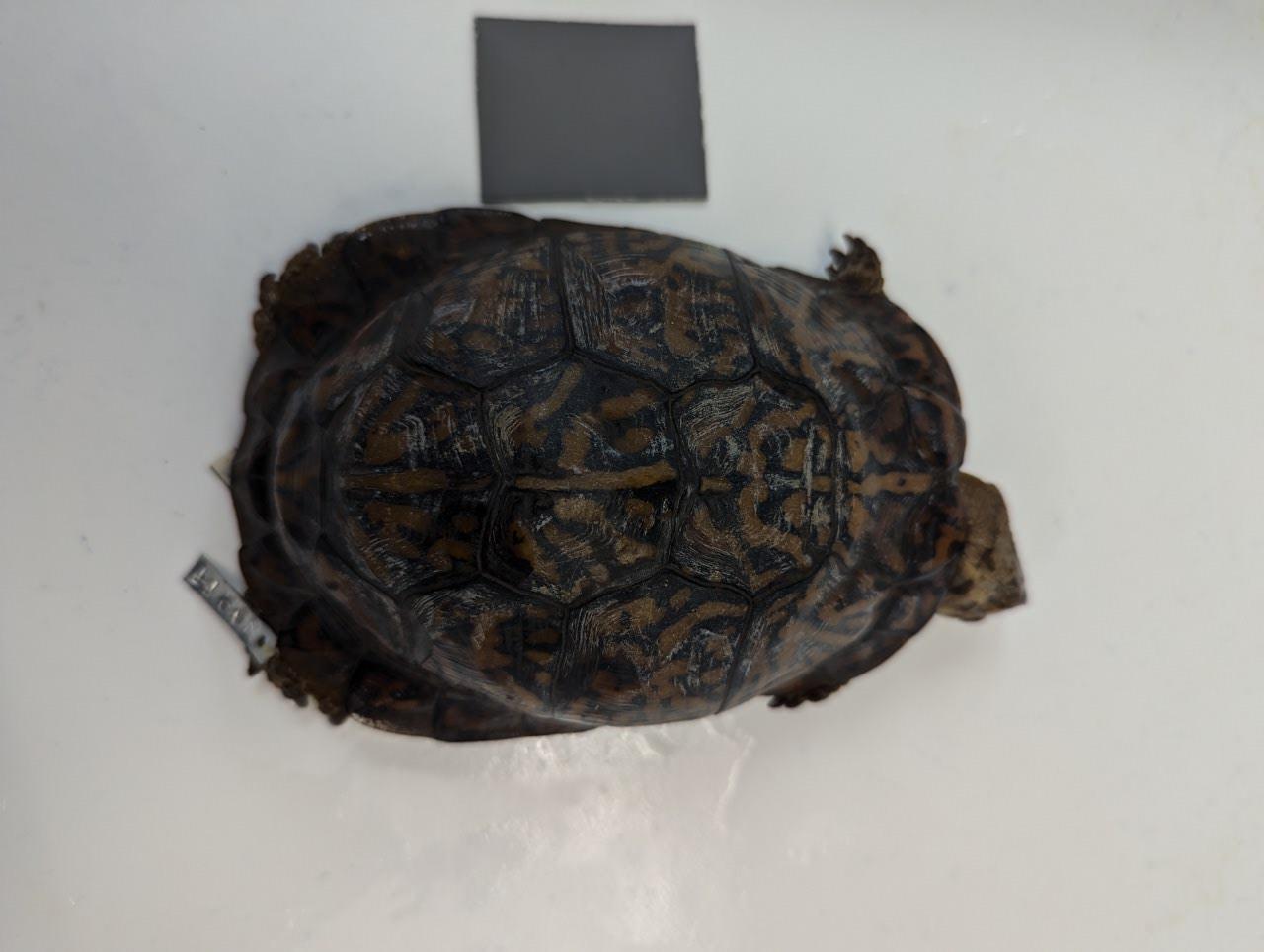

Supplement: Supplemental Information 6 — 98 photos of 98 turtles (single photo, all top view) that were used for the Citizen Science classification analysis. [file peerj-13-19690-s006.zip › TurtleClassification/TURTLE1006.jpg]

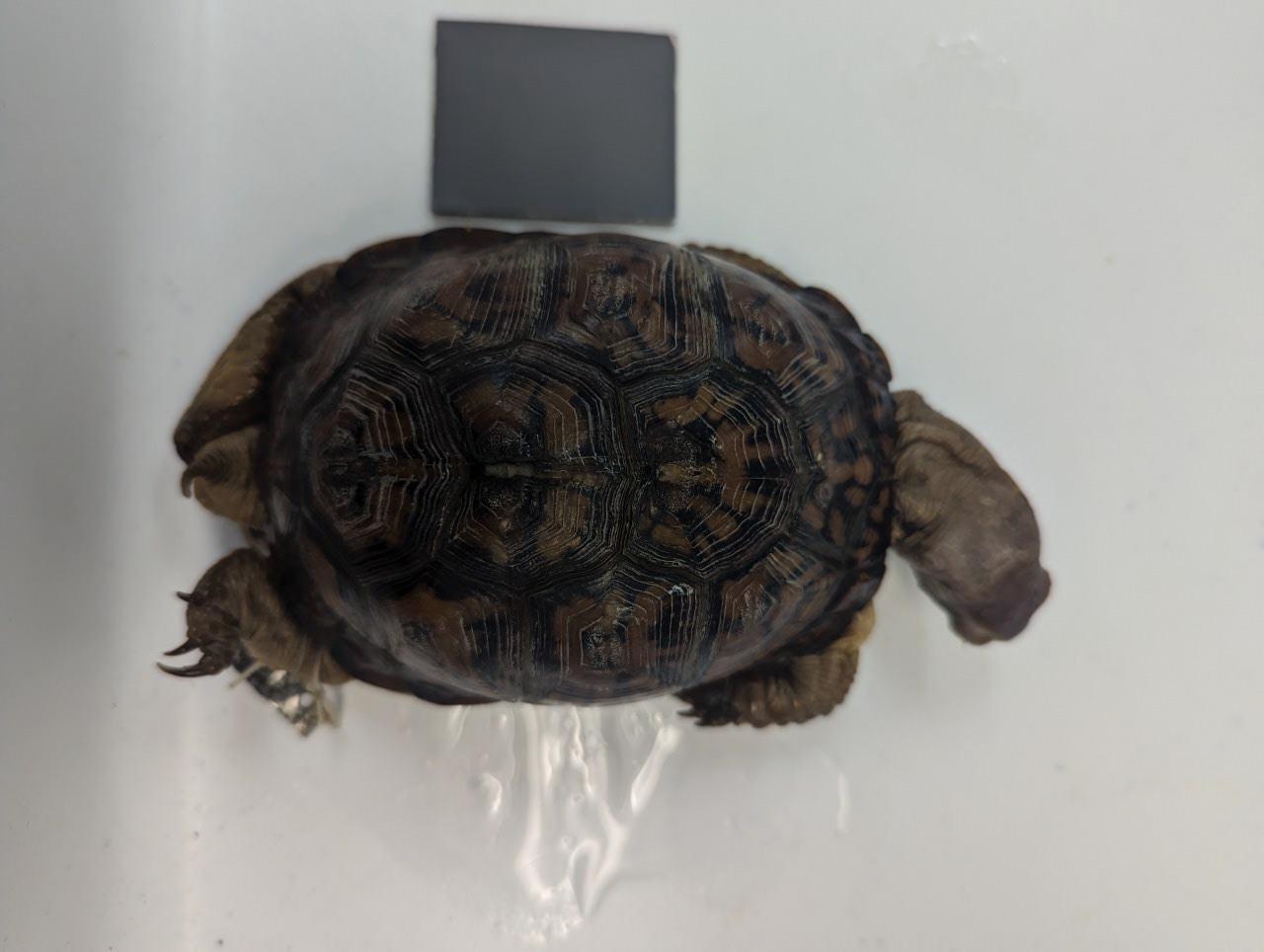

Supplement: Supplemental Information 6 — 98 photos of 98 turtles (single photo, all top view) that were used for the Citizen Science classification analysis. [file peerj-13-19690-s006.zip › TurtleClassification/TURTLE1007.jpg]

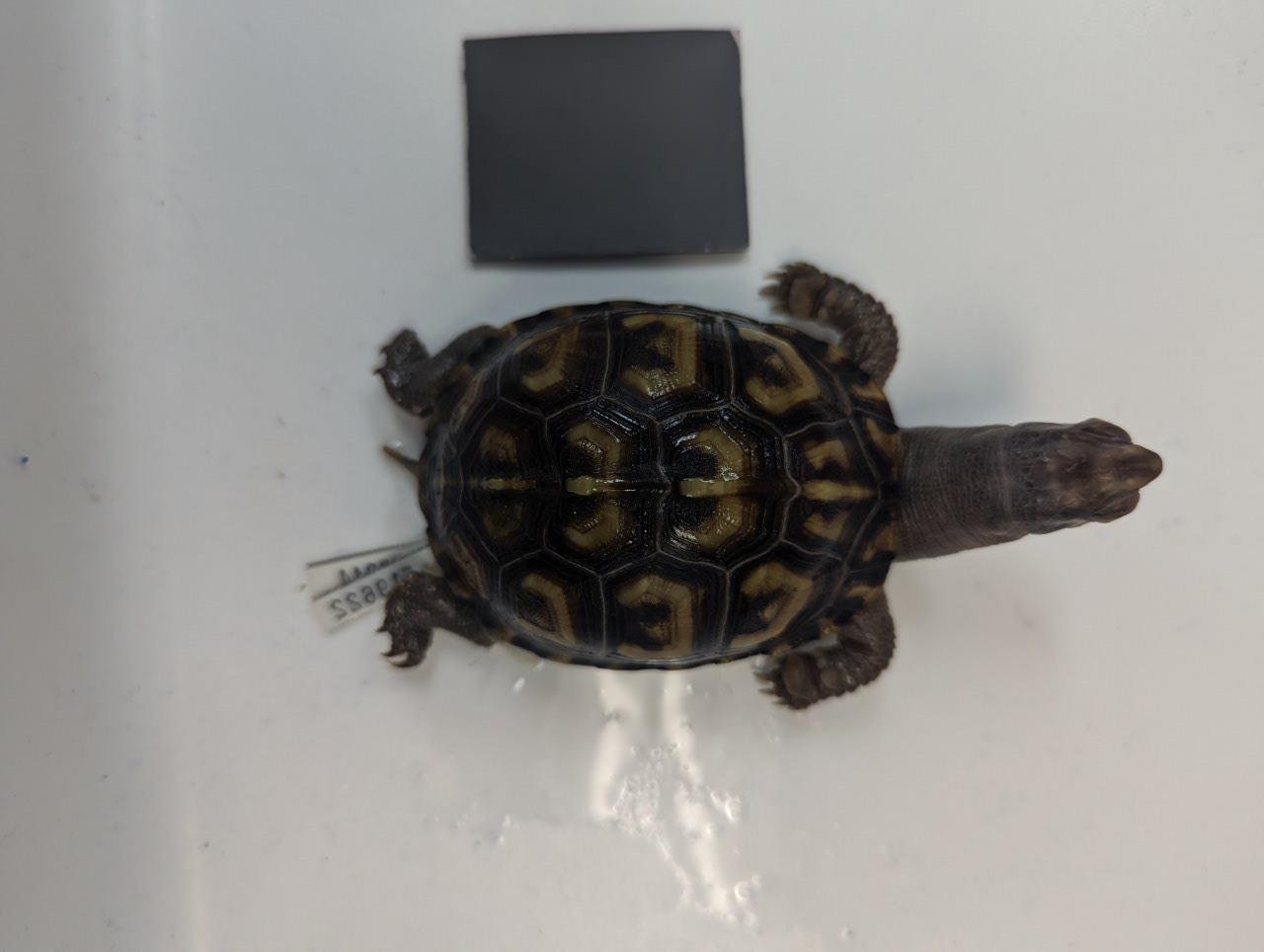

Supplement: Supplemental Information 6 — 98 photos of 98 turtles (single photo, all top view) that were used for the Citizen Science classification analysis. [file peerj-13-19690-s006.zip › TurtleClassification/TURTLE1008.jpg]

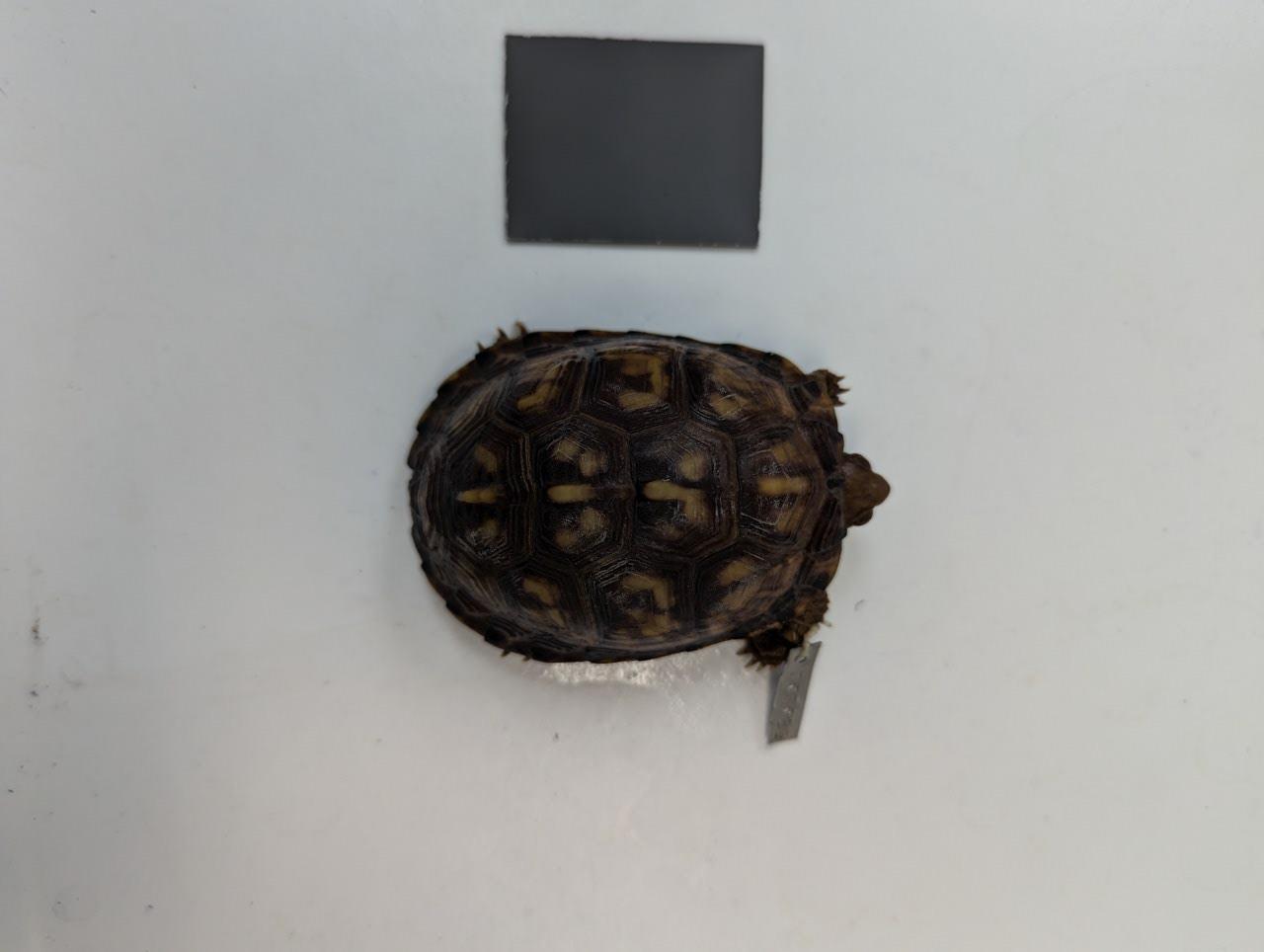

Supplement: Supplemental Information 6 — 98 photos of 98 turtles (single photo, all top view) that were used for the Citizen Science classification analysis. [file peerj-13-19690-s006.zip › TurtleClassification/TURTLE1009.jpg]

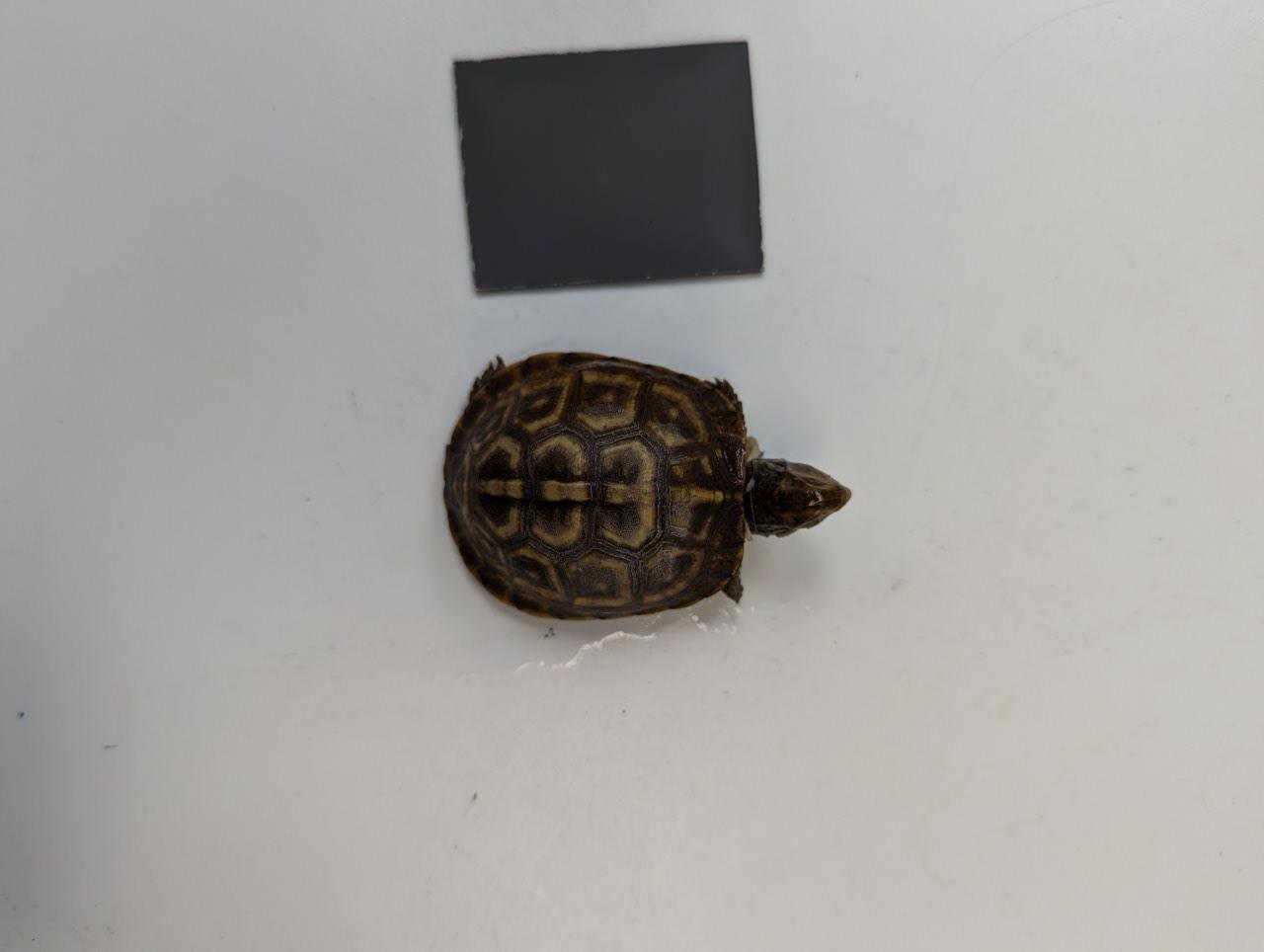

Supplement: Supplemental Information 6 — 98 photos of 98 turtles (single photo, all top view) that were used for the Citizen Science classification analysis. [file peerj-13-19690-s006.zip › TurtleClassification/TURTLE1010.jpg]

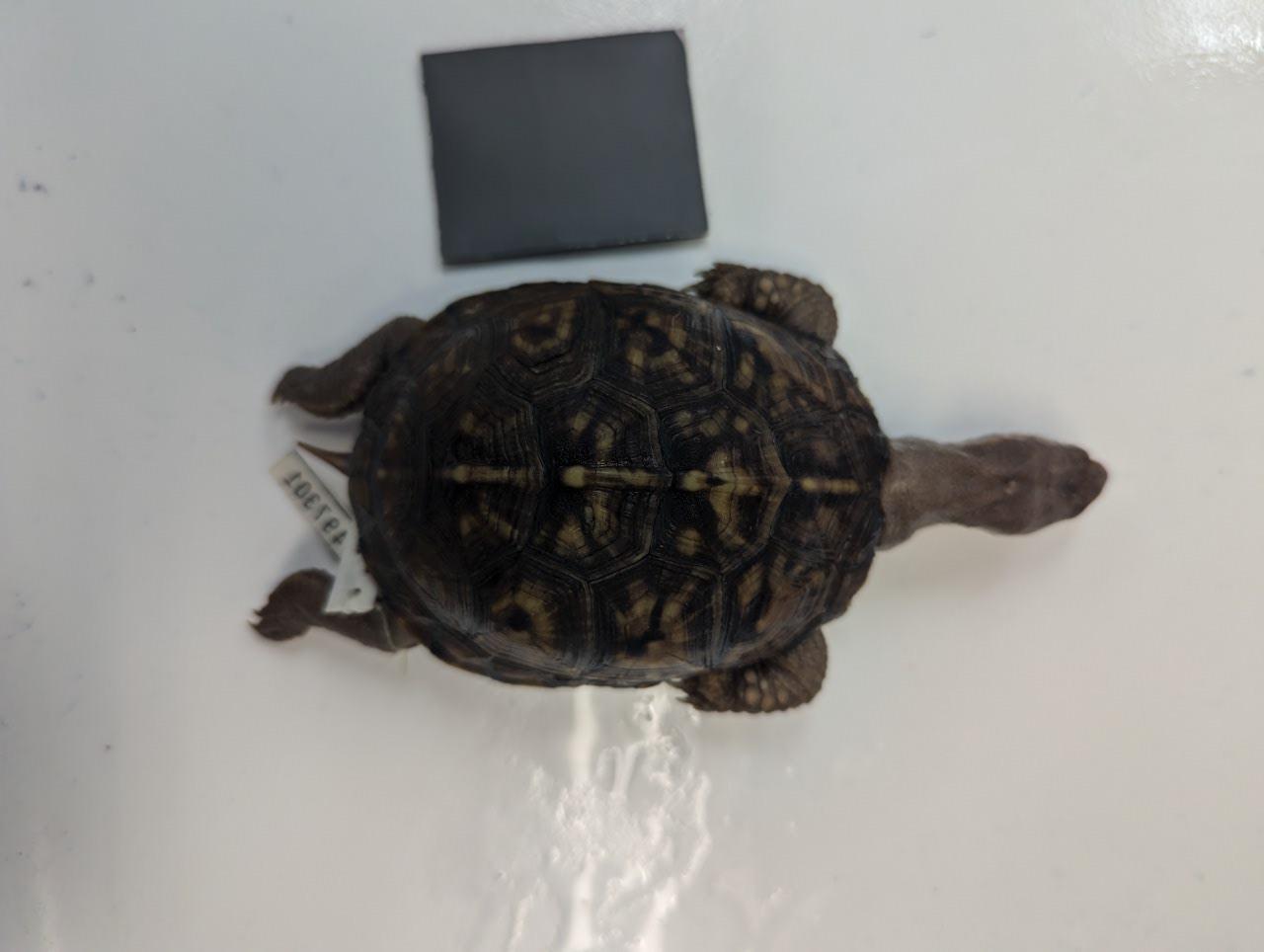

Supplement: Supplemental Information 6 — 98 photos of 98 turtles (single photo, all top view) that were used for the Citizen Science classification analysis. [file peerj-13-19690-s006.zip › TurtleClassification/TURTLE1011.jpg]

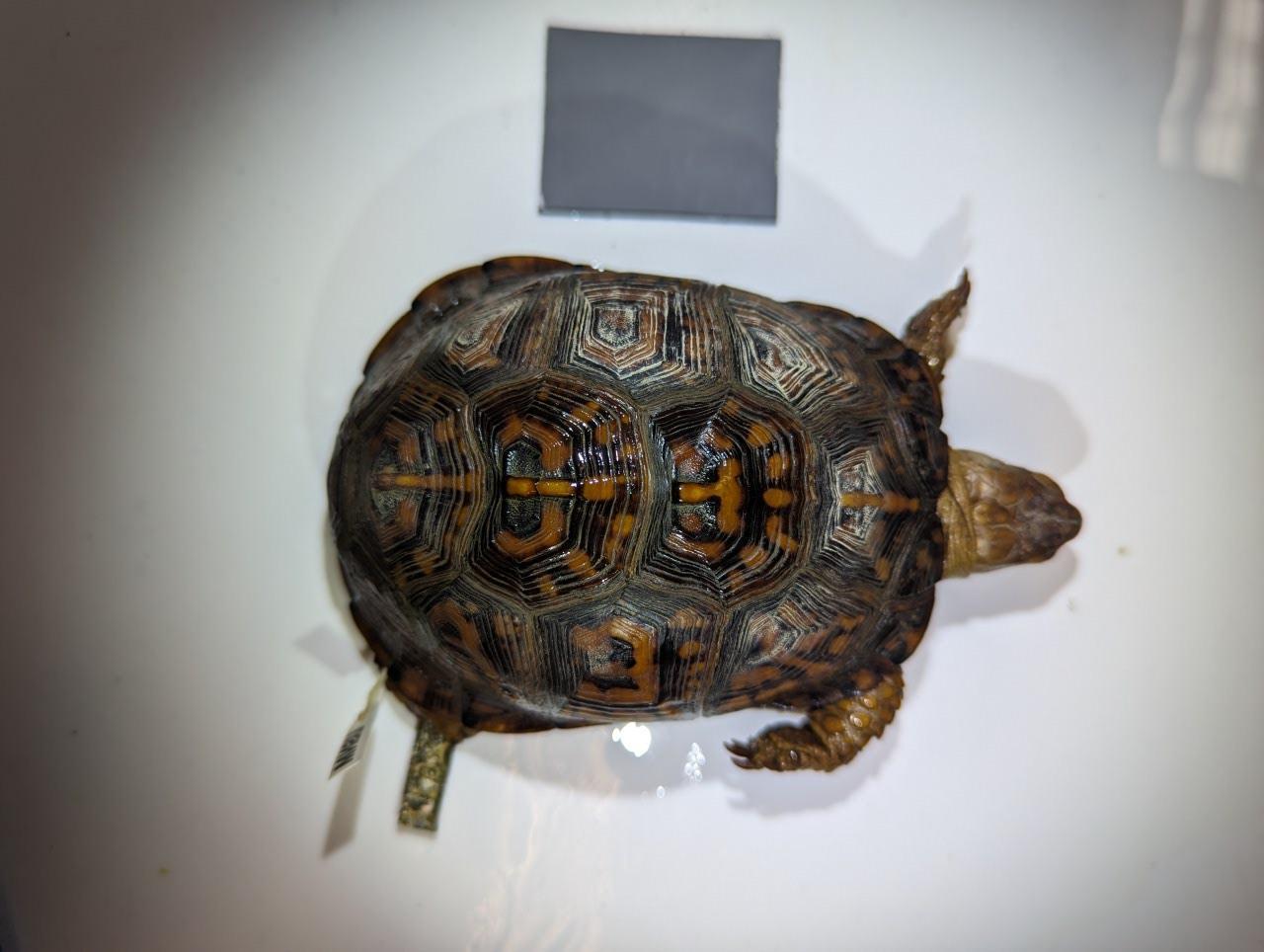

Supplement: Supplemental Information 6 — 98 photos of 98 turtles (single photo, all top view) that were used for the Citizen Science classification analysis. [file peerj-13-19690-s006.zip › TurtleClassification/TURTLE1012.jpg]

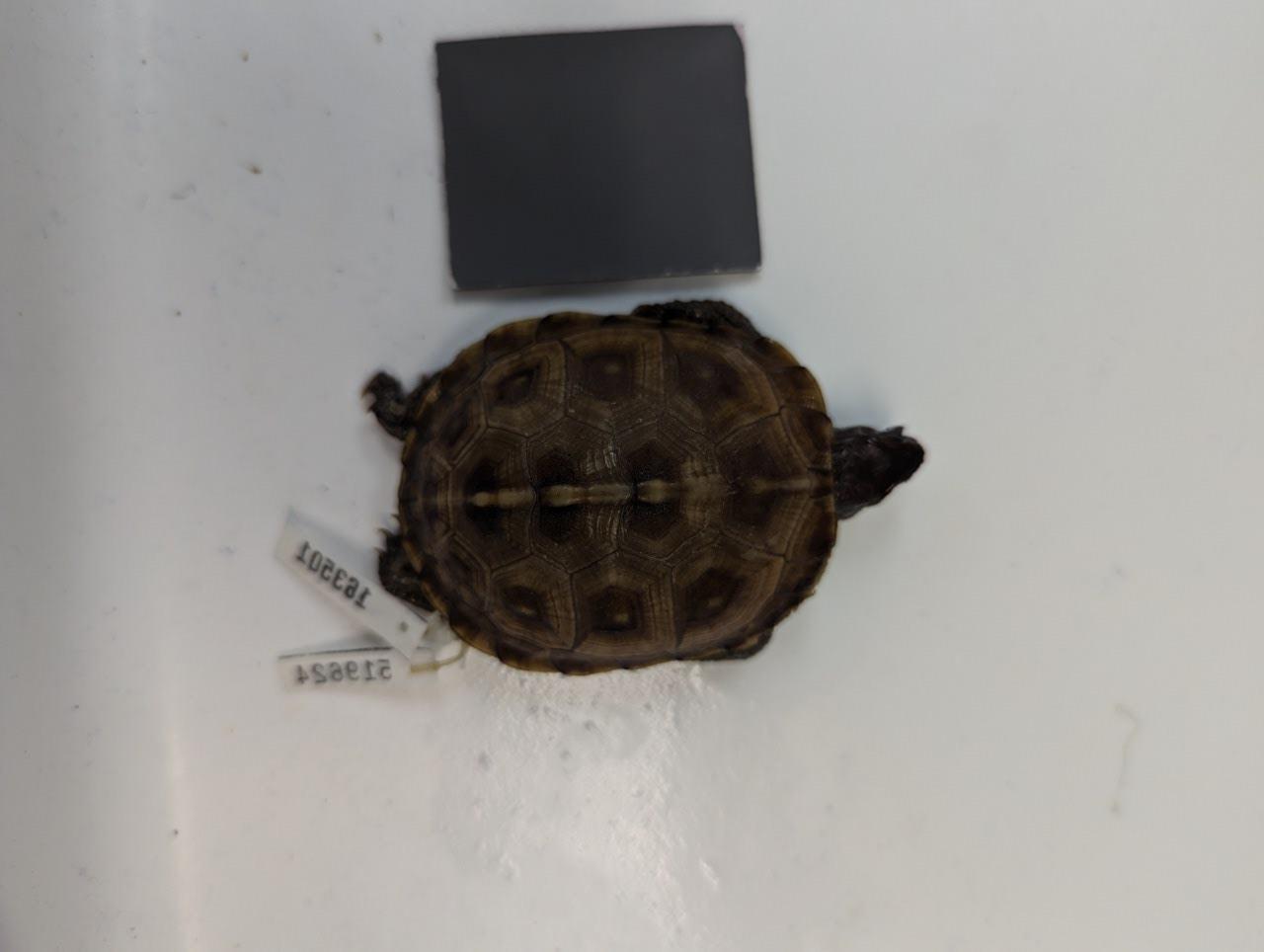

Supplement: Supplemental Information 6 — 98 photos of 98 turtles (single photo, all top view) that were used for the Citizen Science classification analysis. [file peerj-13-19690-s006.zip › TurtleClassification/TURTLE1013.jpg]

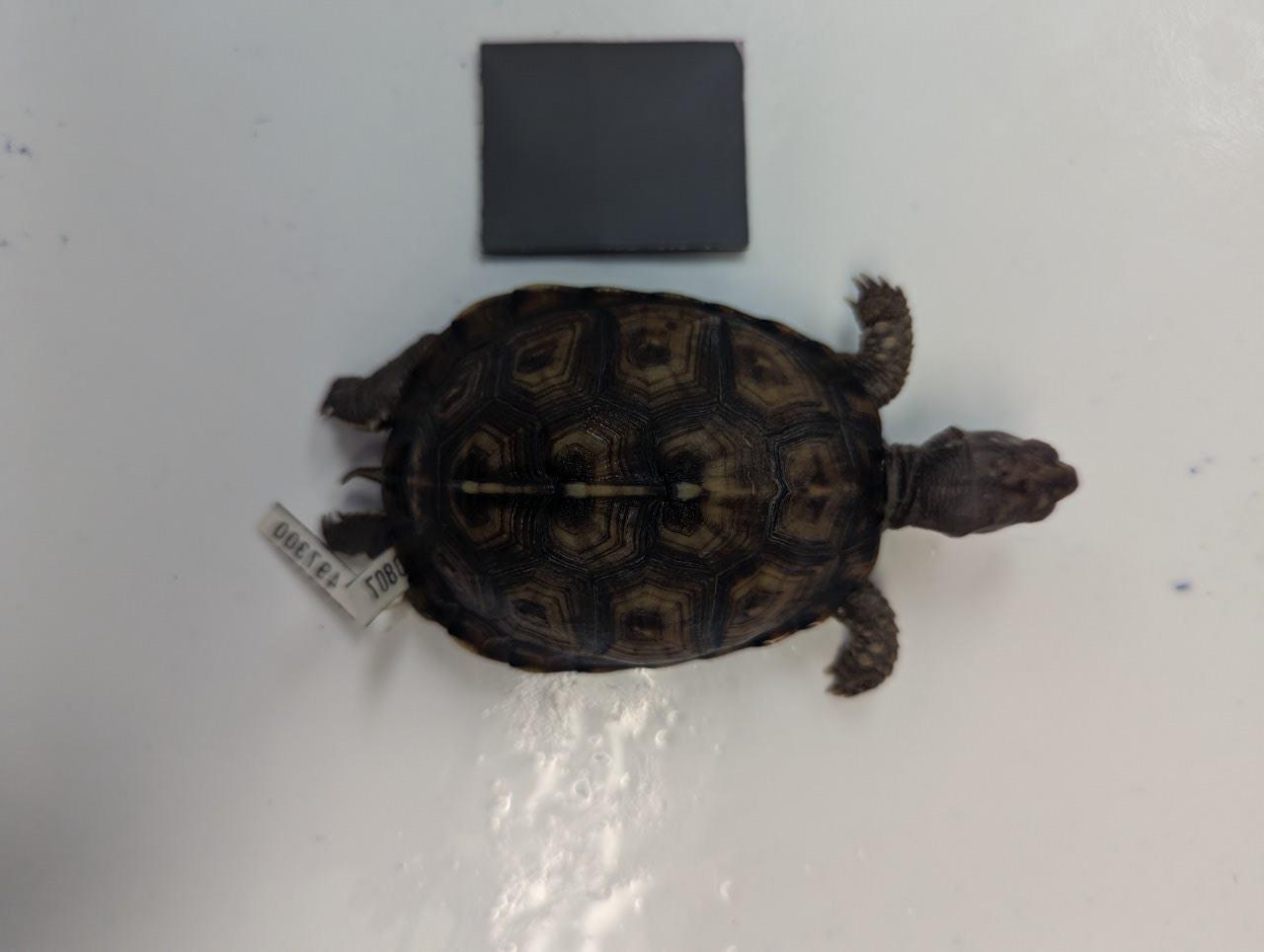

Supplement: Supplemental Information 6 — 98 photos of 98 turtles (single photo, all top view) that were used for the Citizen Science classification analysis. [file peerj-13-19690-s006.zip › TurtleClassification/TURTLE1014.jpg]

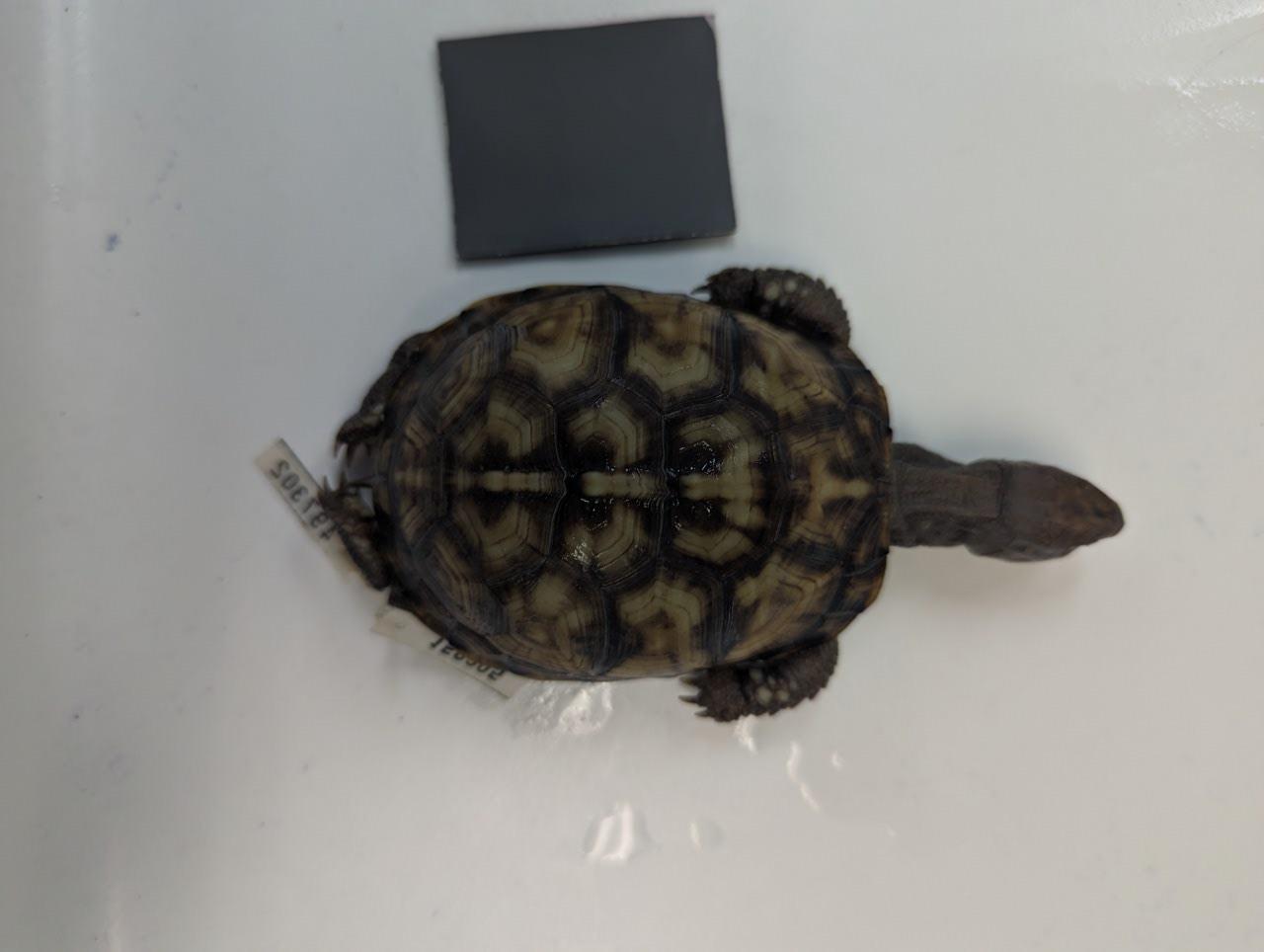

Supplement: Supplemental Information 6 — 98 photos of 98 turtles (single photo, all top view) that were used for the Citizen Science classification analysis. [file peerj-13-19690-s006.zip › TurtleClassification/TURTLE1015.jpg]

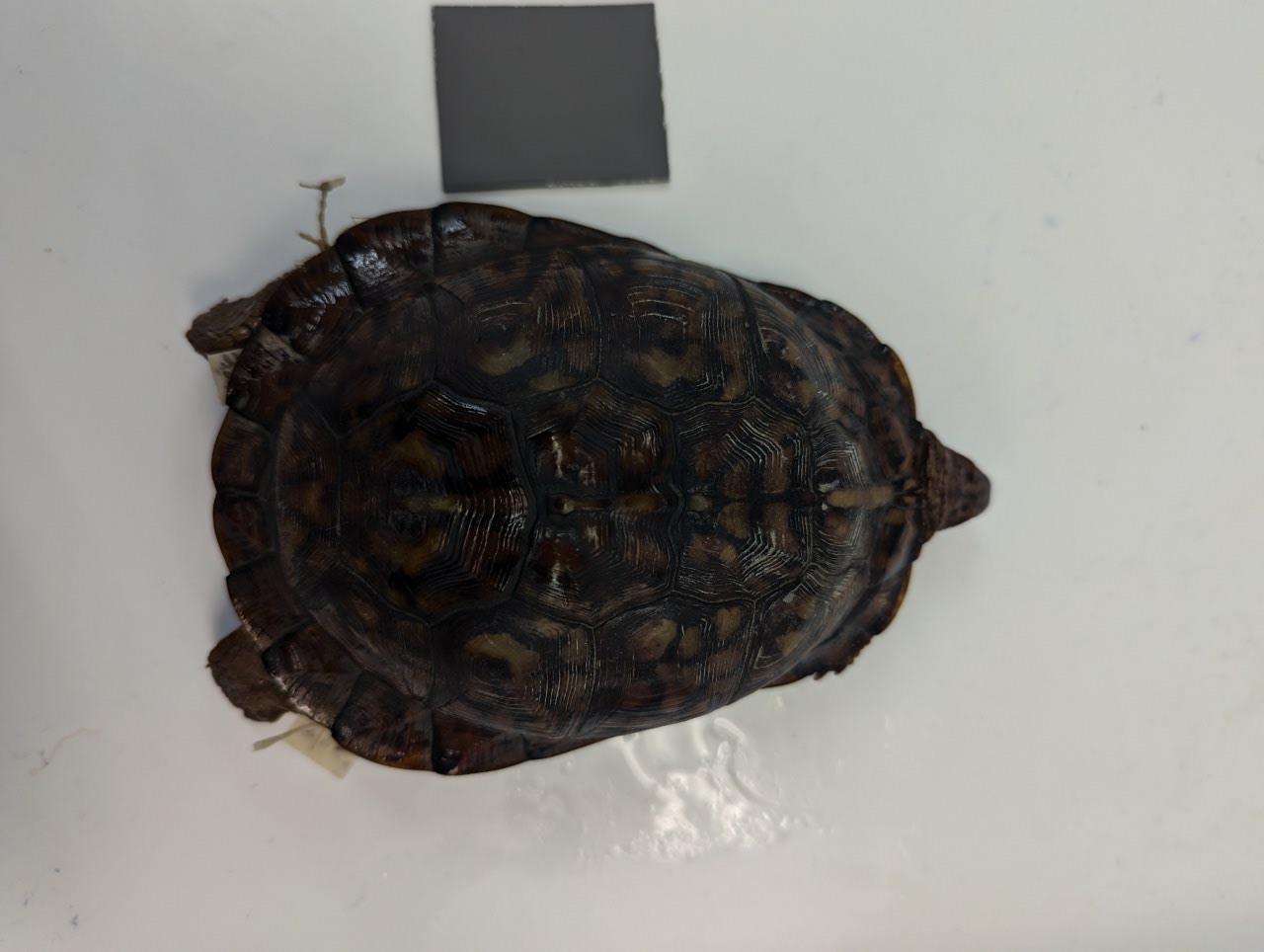

Supplement: Supplemental Information 6 — 98 photos of 98 turtles (single photo, all top view) that were used for the Citizen Science classification analysis. [file peerj-13-19690-s006.zip › TurtleClassification/TURTLE1016.jpg]

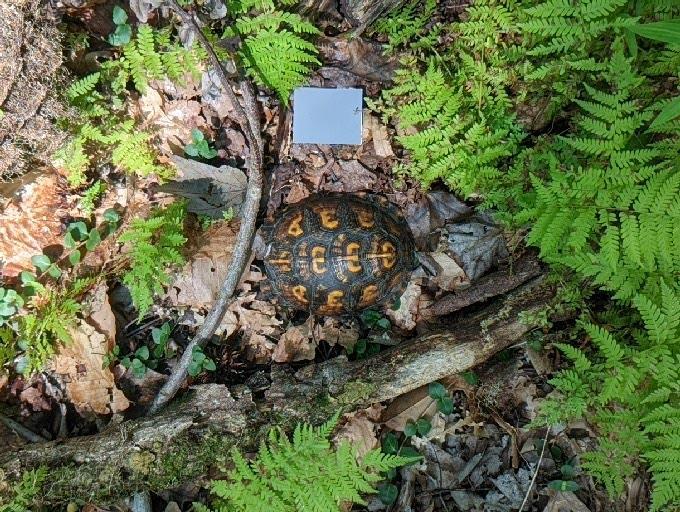

Supplement: Supplemental Information 6 — 98 photos of 98 turtles (single photo, all top view) that were used for the Citizen Science classification analysis. [file peerj-13-19690-s006.zip › TurtleClassification/TURTLE1017.jpg]

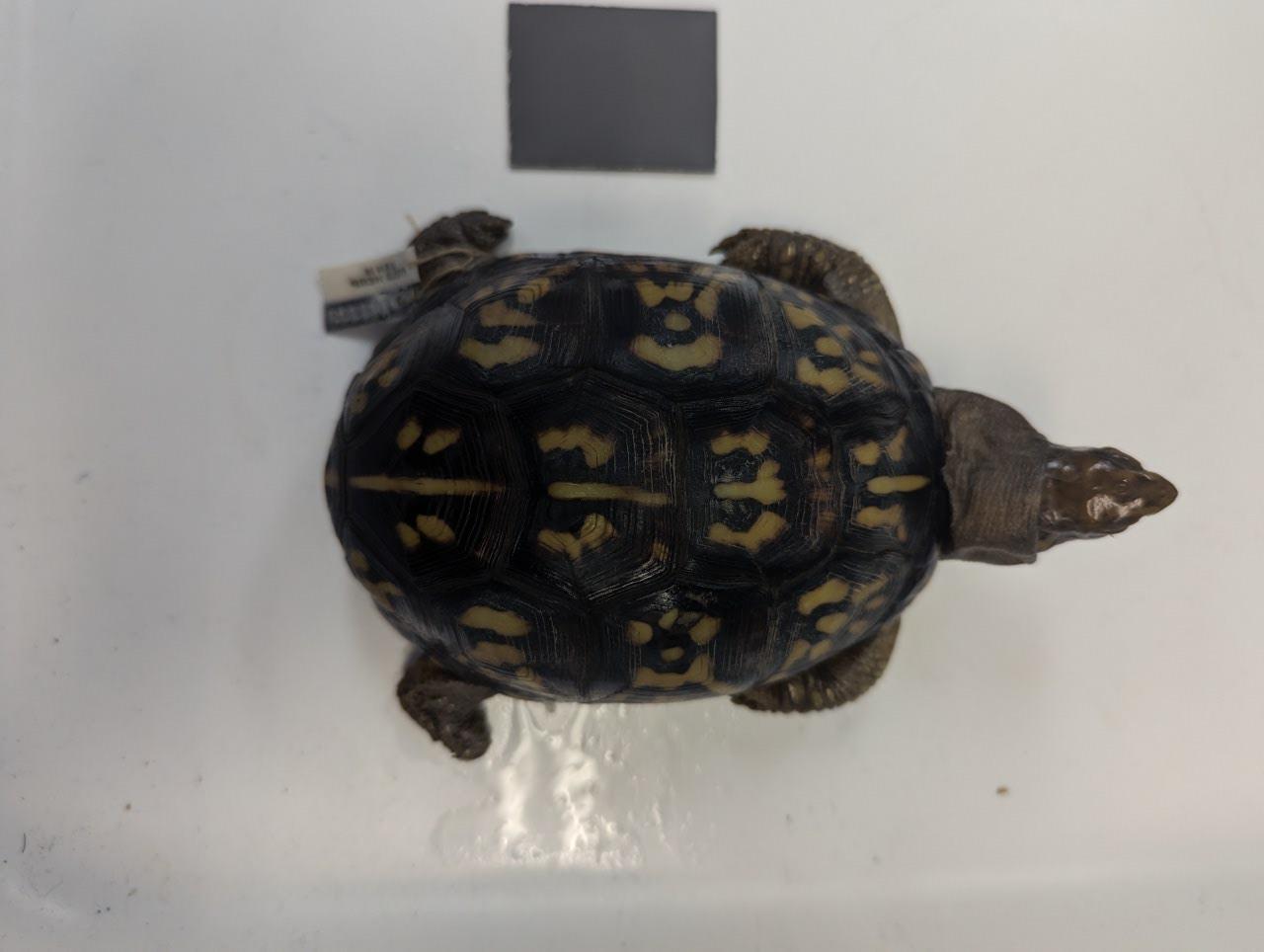

Supplement: Supplemental Information 6 — 98 photos of 98 turtles (single photo, all top view) that were used for the Citizen Science classification analysis. [file peerj-13-19690-s006.zip › TurtleClassification/TURTLE1018.jpg]

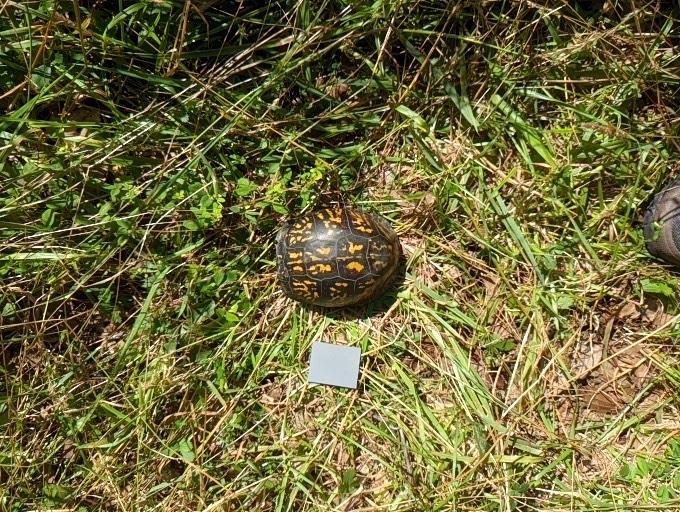

Supplement: Supplemental Information 6 — 98 photos of 98 turtles (single photo, all top view) that were used for the Citizen Science classification analysis. [file peerj-13-19690-s006.zip › TurtleClassification/TURTLE1019.jpg]

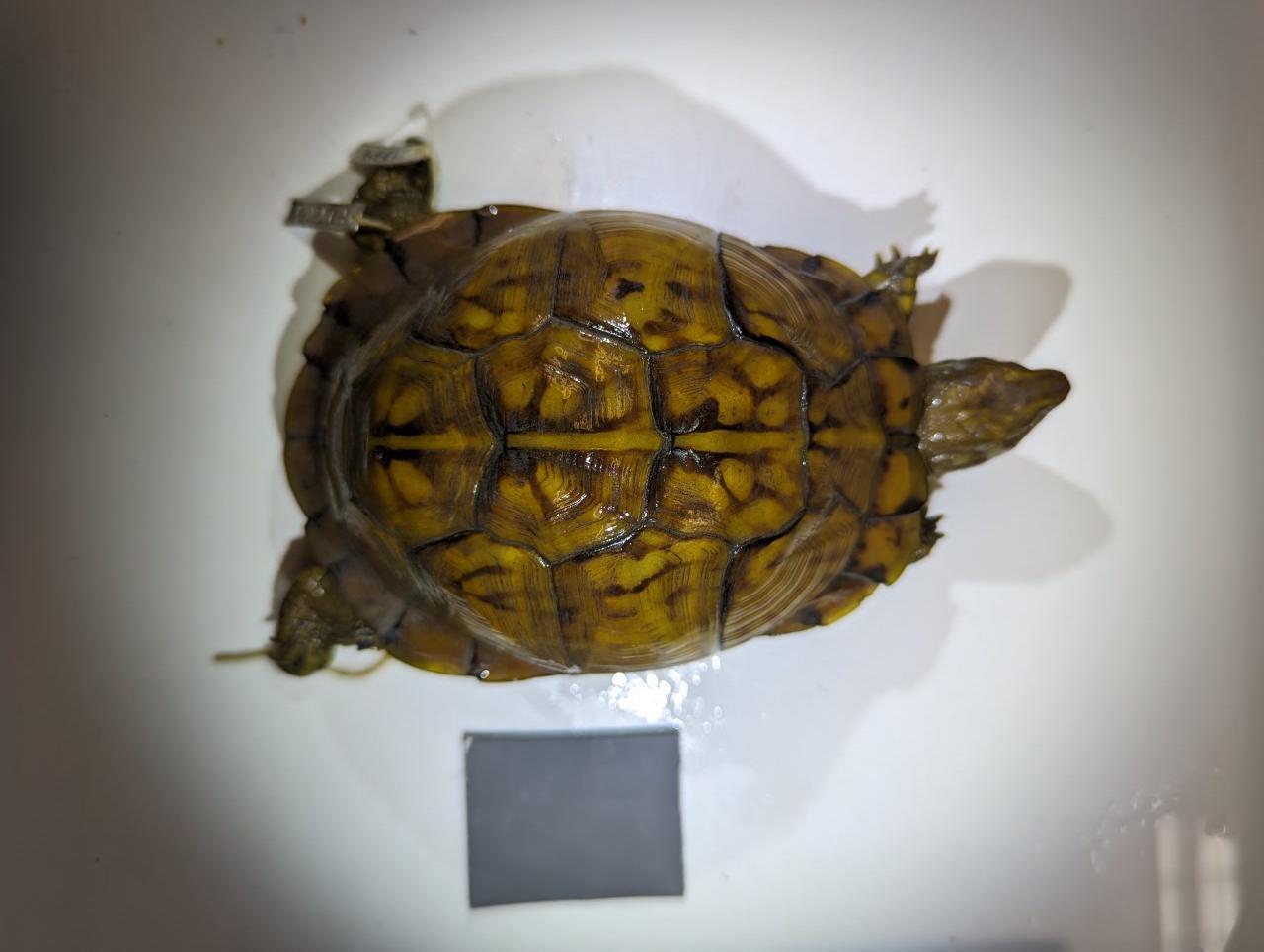

Supplement: Supplemental Information 6 — 98 photos of 98 turtles (single photo, all top view) that were used for the Citizen Science classification analysis. [file peerj-13-19690-s006.zip › TurtleClassification/TURTLE1020.jpg]

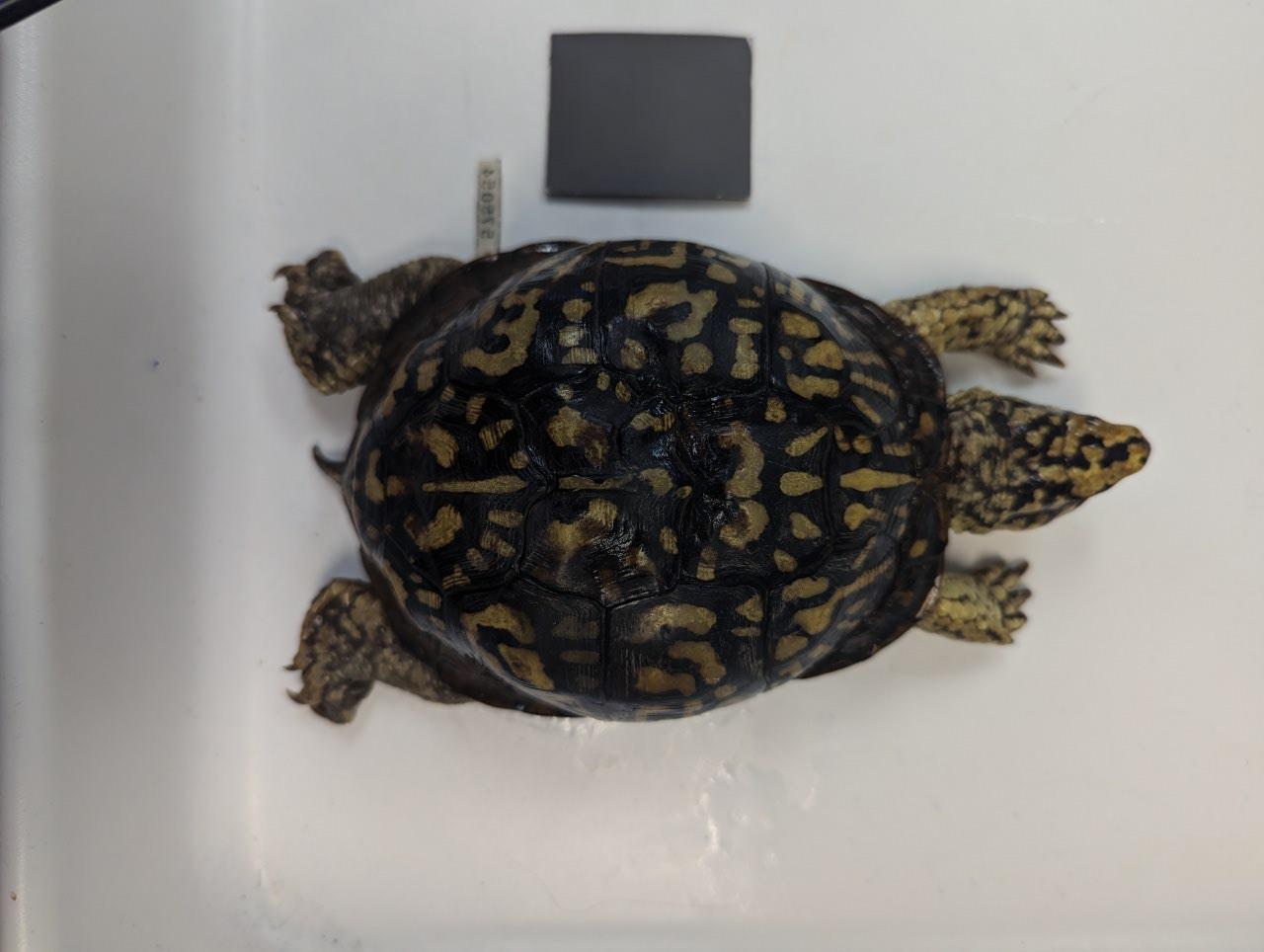

Supplement: Supplemental Information 6 — 98 photos of 98 turtles (single photo, all top view) that were used for the Citizen Science classification analysis. [file peerj-13-19690-s006.zip › TurtleClassification/TURTLE1021.jpg]

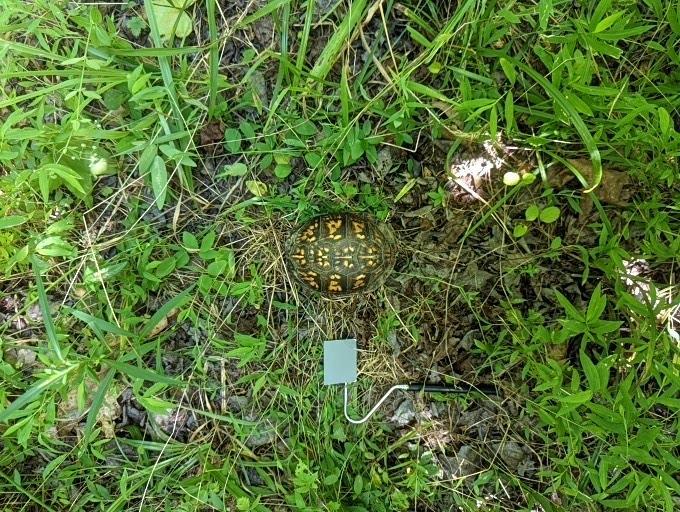

Supplement: Supplemental Information 6 — 98 photos of 98 turtles (single photo, all top view) that were used for the Citizen Science classification analysis. [file peerj-13-19690-s006.zip › TurtleClassification/TURTLE1022.jpg]

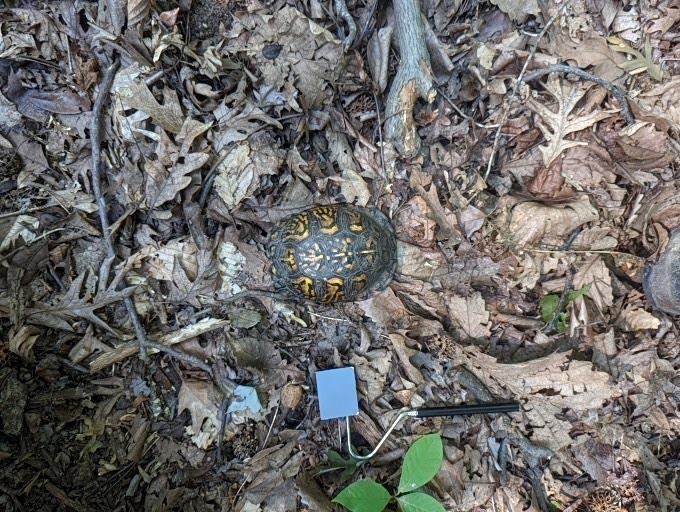

Supplement: Supplemental Information 6 — 98 photos of 98 turtles (single photo, all top view) that were used for the Citizen Science classification analysis. [file peerj-13-19690-s006.zip › TurtleClassification/TURTLE1023.jpg]

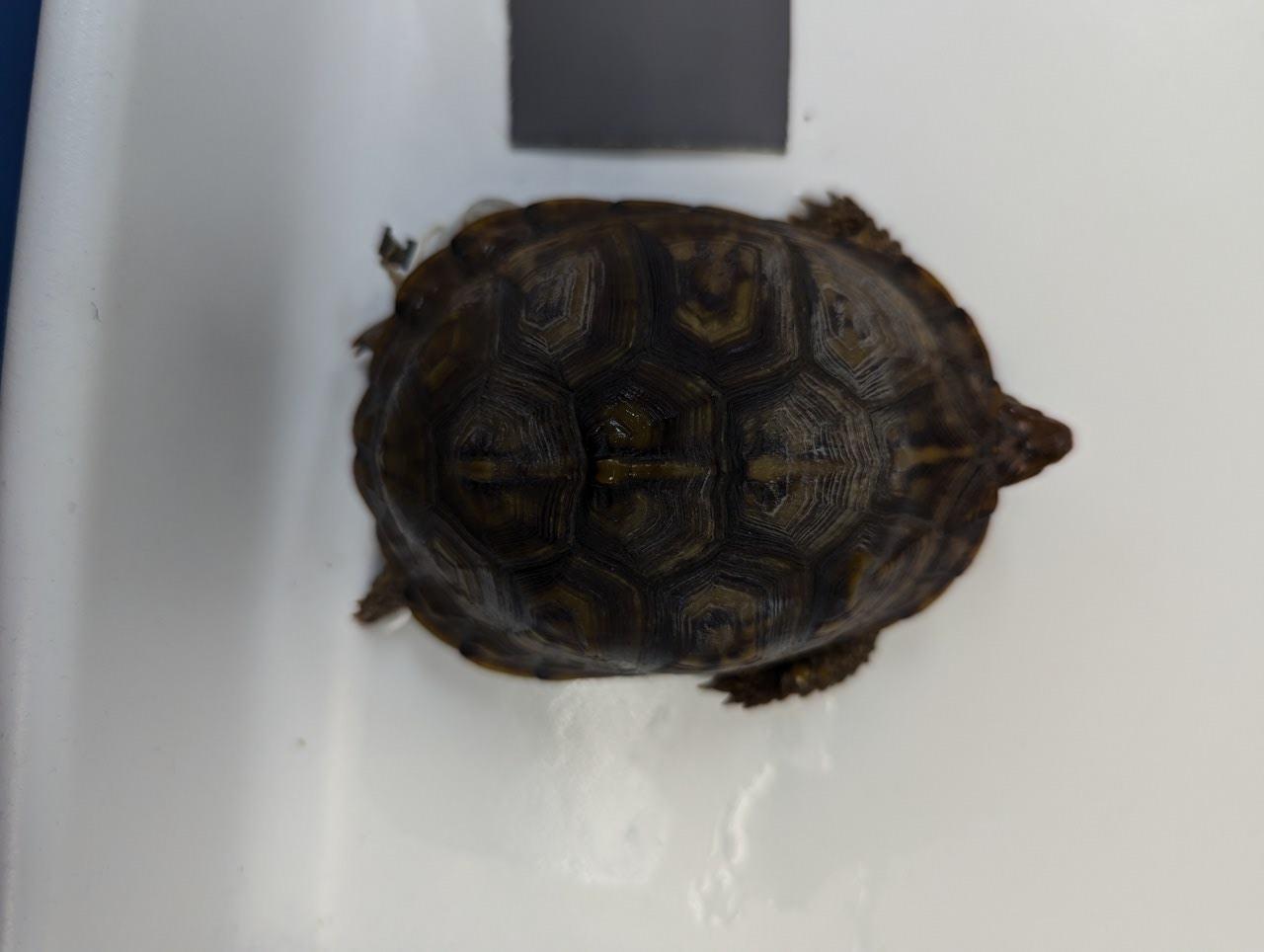

Supplement: Supplemental Information 6 — 98 photos of 98 turtles (single photo, all top view) that were used for the Citizen Science classification analysis. [file peerj-13-19690-s006.zip › TurtleClassification/TURTLE1024.jpg]

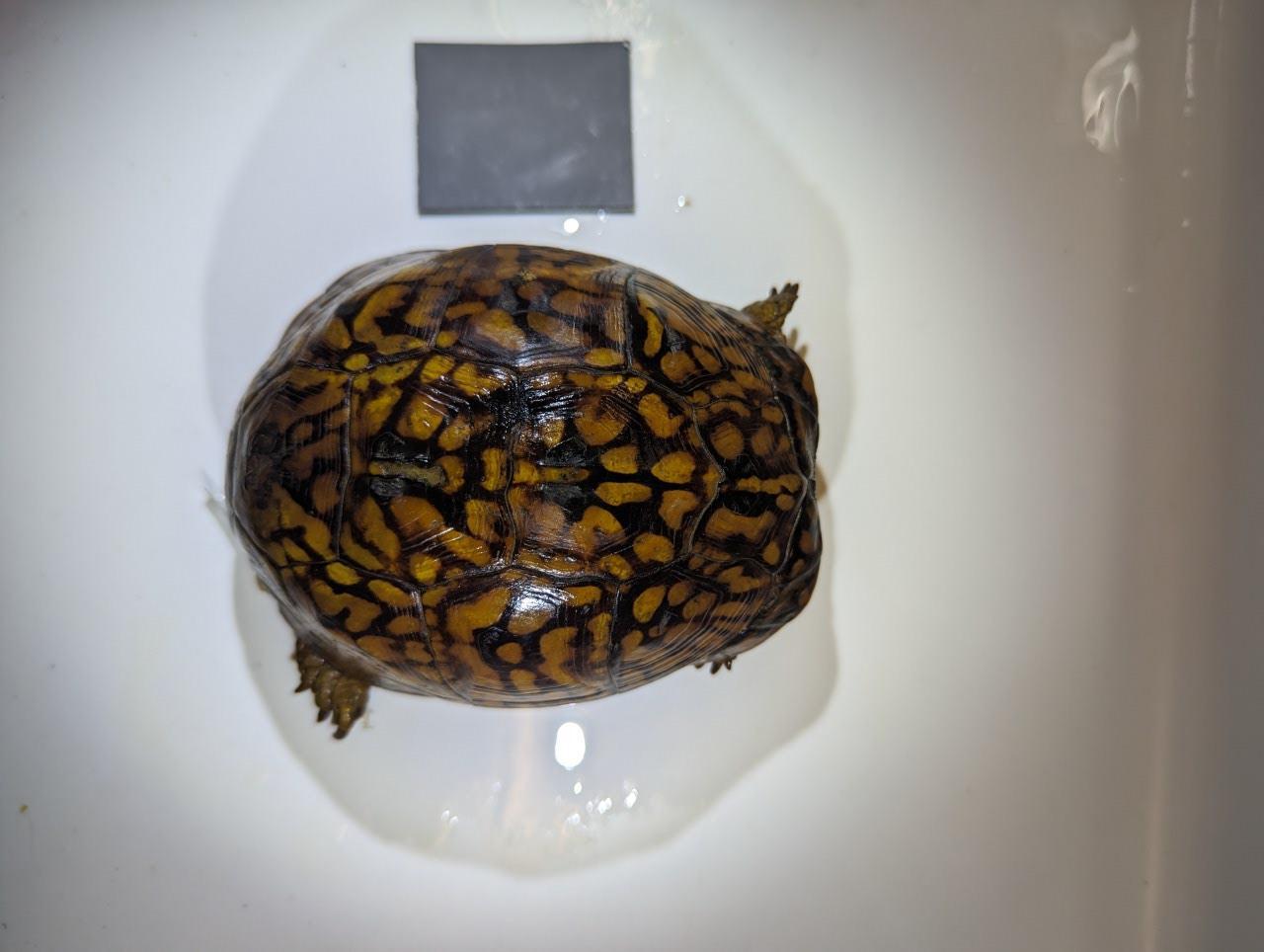

Supplement: Supplemental Information 6 — 98 photos of 98 turtles (single photo, all top view) that were used for the Citizen Science classification analysis. [file peerj-13-19690-s006.zip › TurtleClassification/TURTLE1025.jpg]

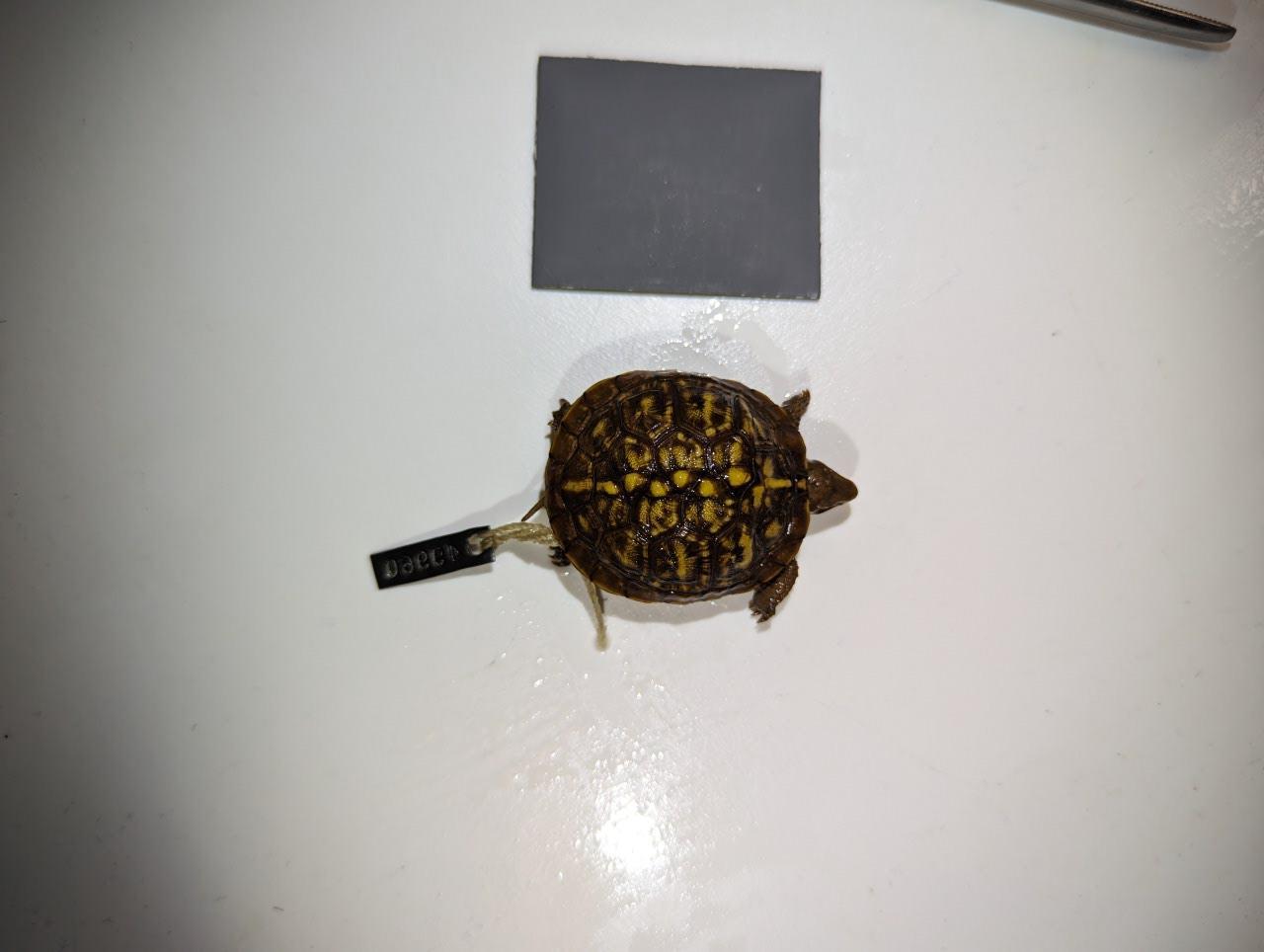

Supplement: Supplemental Information 6 — 98 photos of 98 turtles (single photo, all top view) that were used for the Citizen Science classification analysis. [file peerj-13-19690-s006.zip › TurtleClassification/TURTLE1026.jpg]

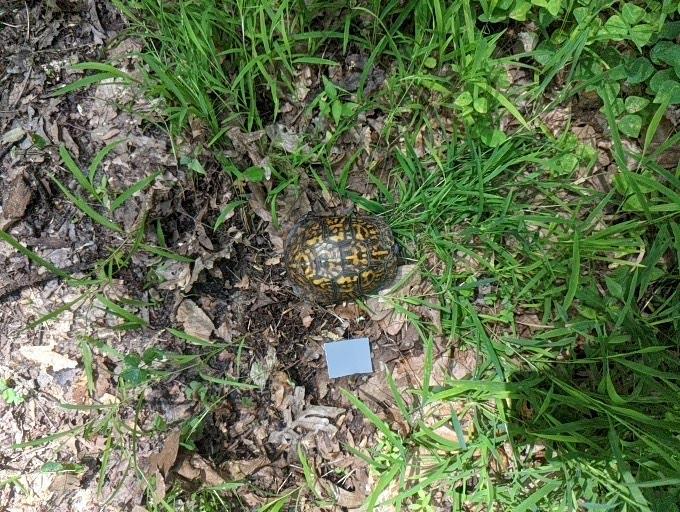

Supplement: Supplemental Information 6 — 98 photos of 98 turtles (single photo, all top view) that were used for the Citizen Science classification analysis. [file peerj-13-19690-s006.zip › TurtleClassification/TURTLE1027.jpg]

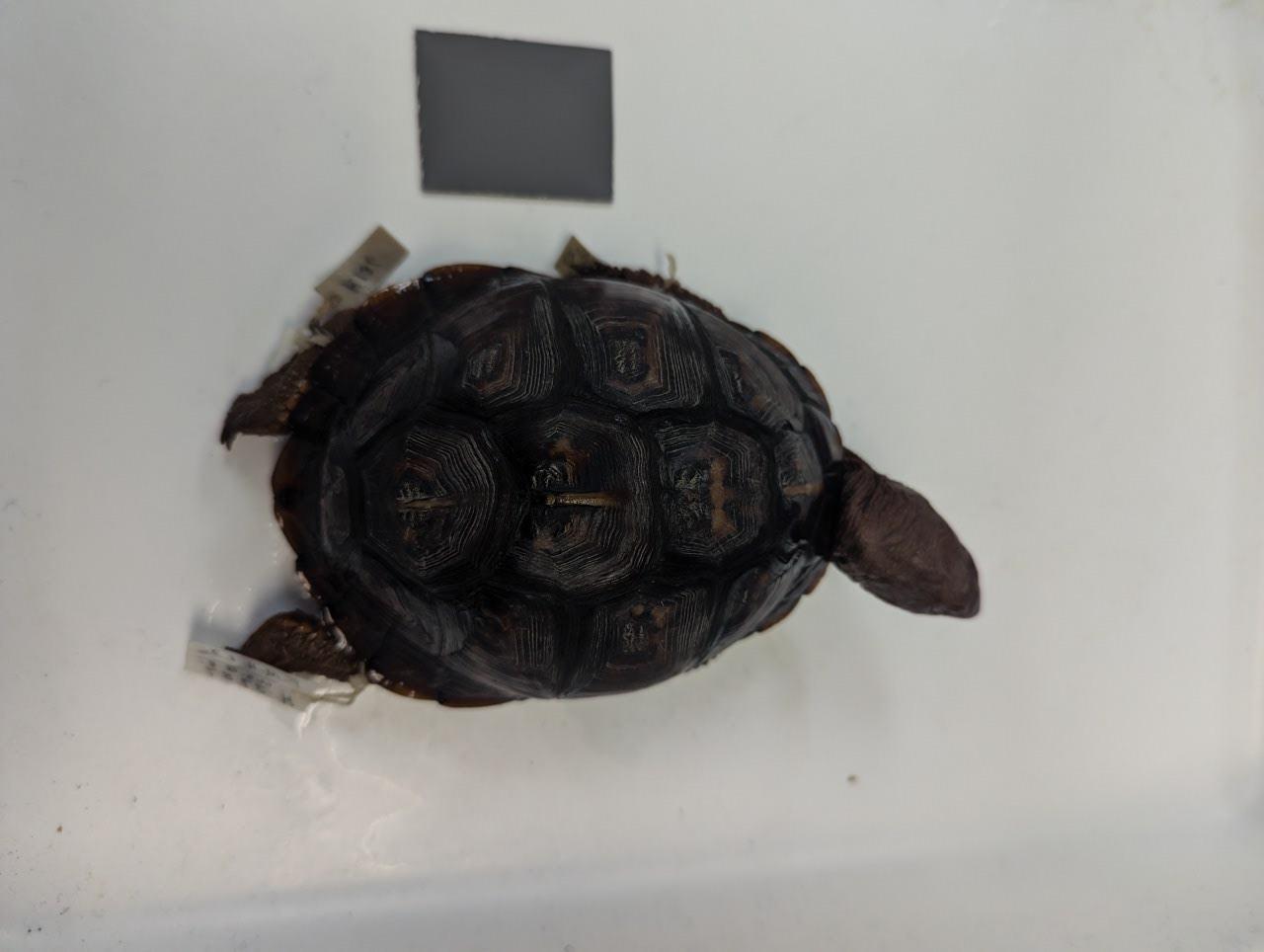

Supplement: Supplemental Information 6 — 98 photos of 98 turtles (single photo, all top view) that were used for the Citizen Science classification analysis. [file peerj-13-19690-s006.zip › TurtleClassification/TURTLE1028.jpg]

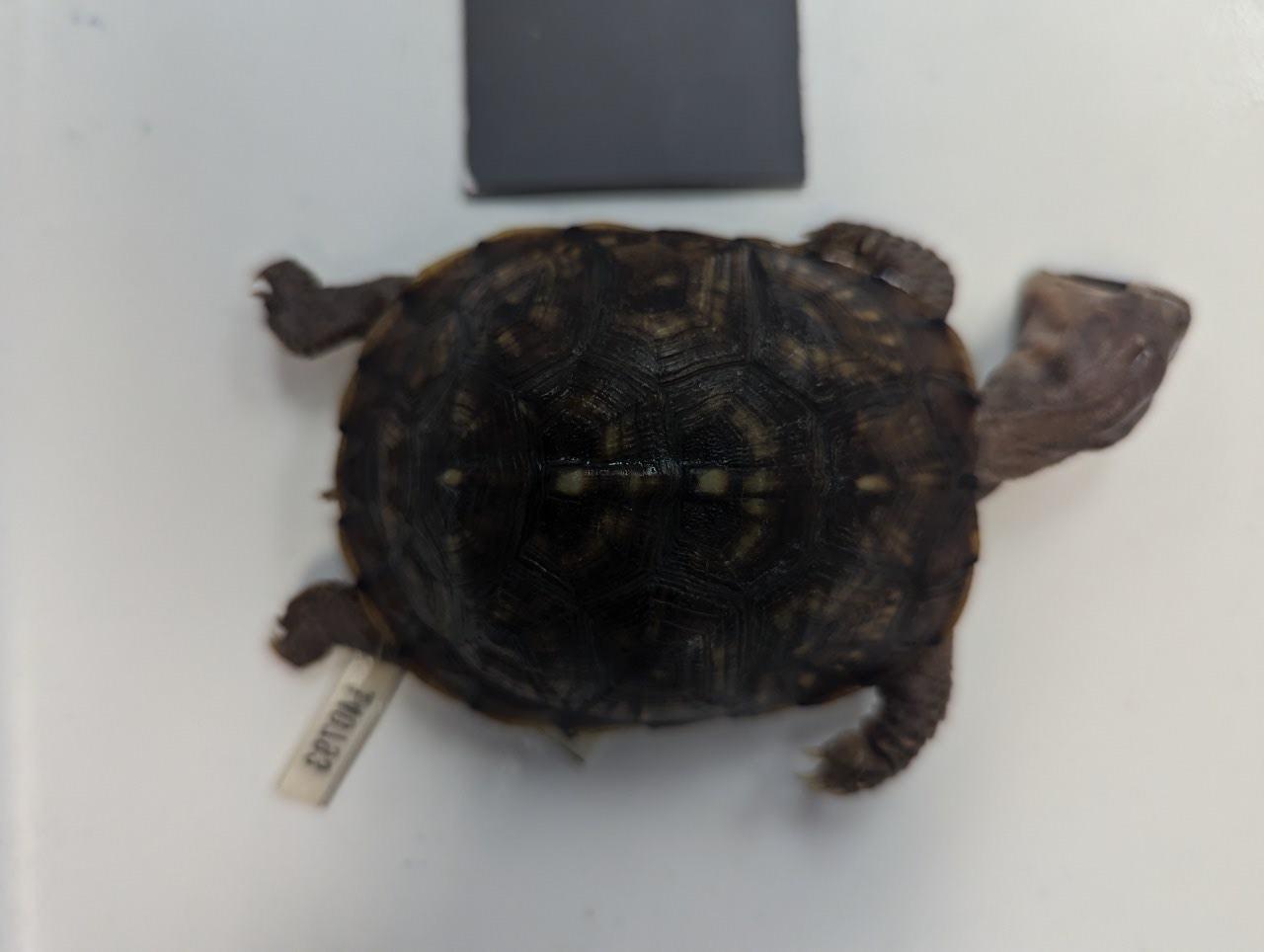

Supplement: Supplemental Information 6 — 98 photos of 98 turtles (single photo, all top view) that were used for the Citizen Science classification analysis. [file peerj-13-19690-s006.zip › TurtleClassification/TURTLE1029.jpg]

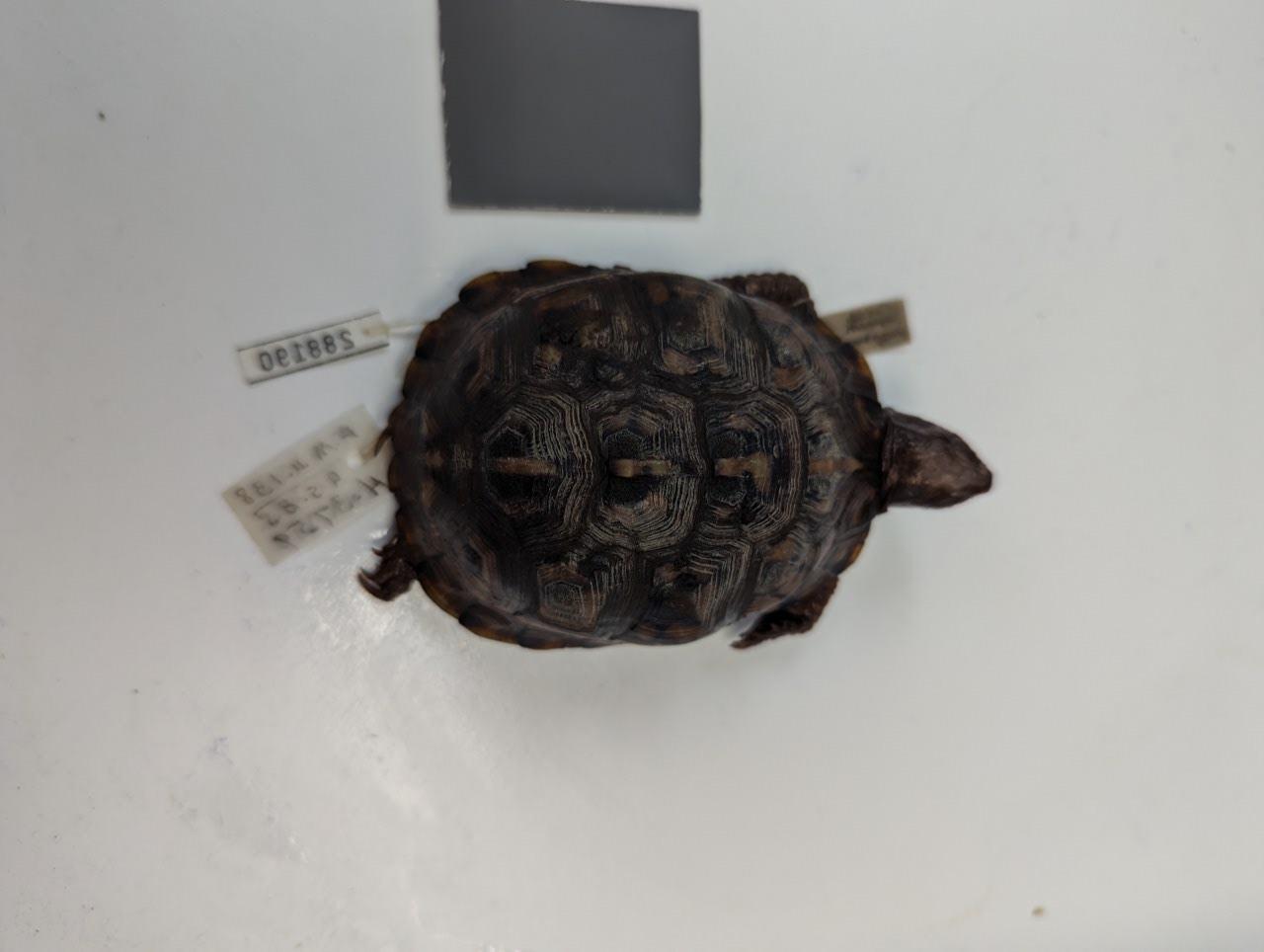

Supplement: Supplemental Information 6 — 98 photos of 98 turtles (single photo, all top view) that were used for the Citizen Science classification analysis. [file peerj-13-19690-s006.zip › TurtleClassification/TURTLE1030.jpg]

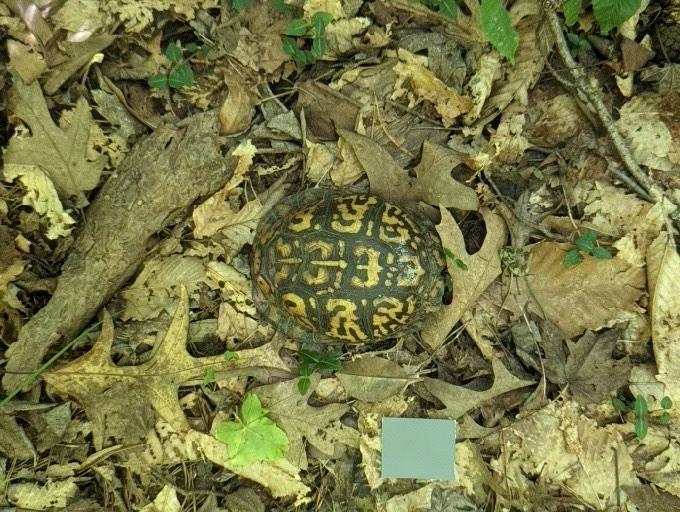

Supplement: Supplemental Information 6 — 98 photos of 98 turtles (single photo, all top view) that were used for the Citizen Science classification analysis. [file peerj-13-19690-s006.zip › TurtleClassification/TURTLE1031.jpg]

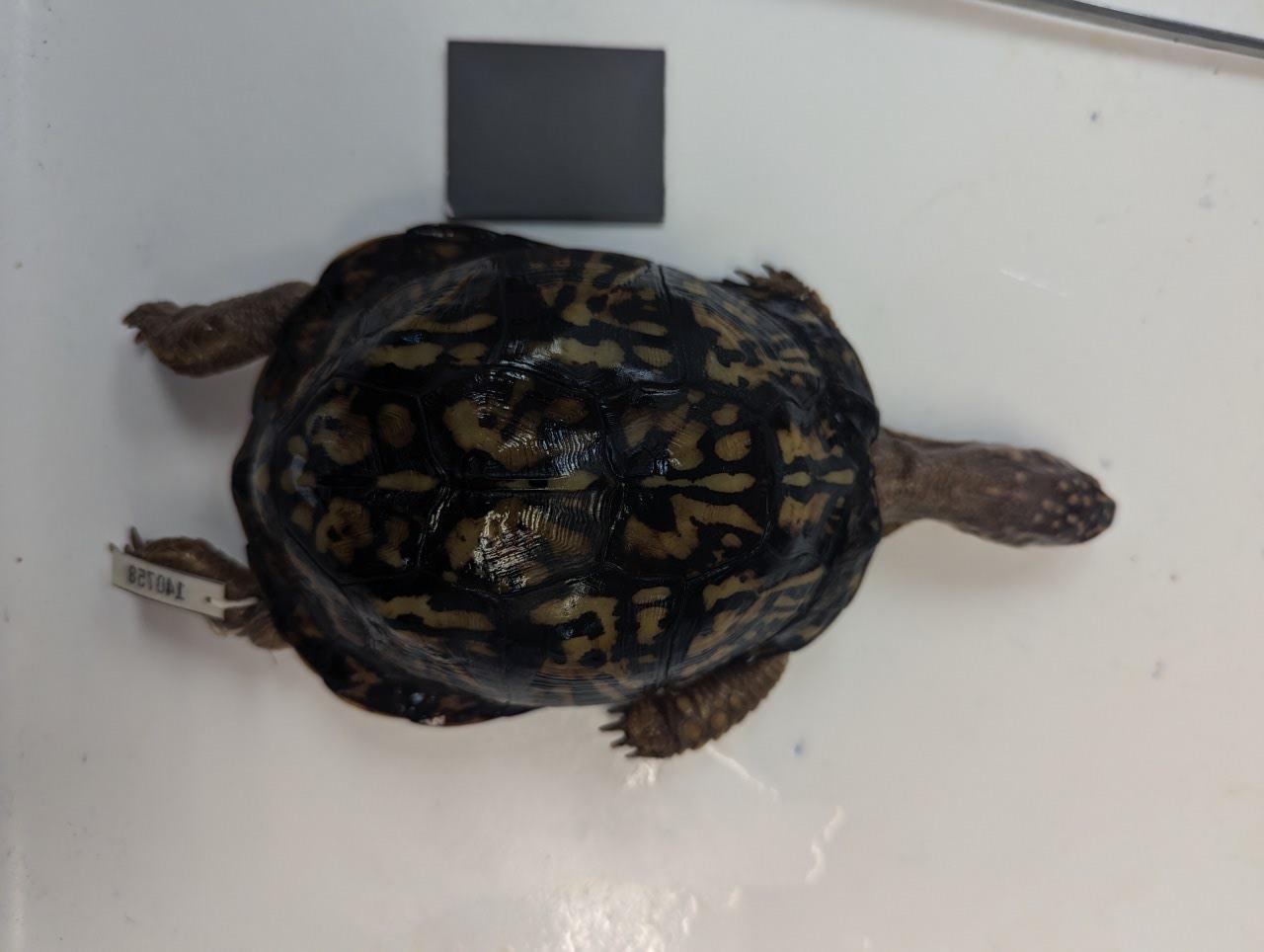

Supplement: Supplemental Information 6 — 98 photos of 98 turtles (single photo, all top view) that were used for the Citizen Science classification analysis. [file peerj-13-19690-s006.zip › TurtleClassification/TURTLE1032.jpg]

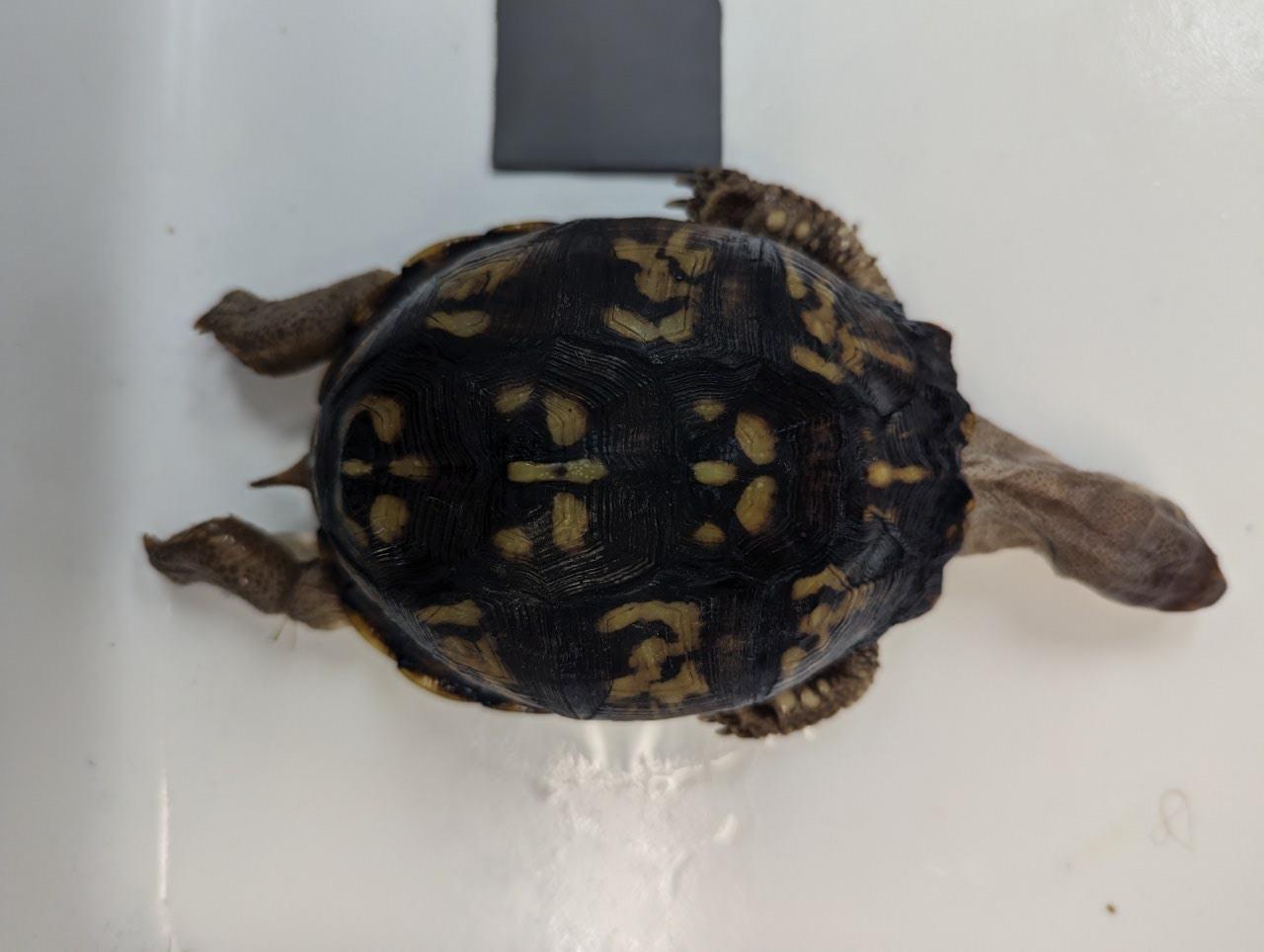

Supplement: Supplemental Information 6 — 98 photos of 98 turtles (single photo, all top view) that were used for the Citizen Science classification analysis. [file peerj-13-19690-s006.zip › TurtleClassification/TURTLE1033.jpg]

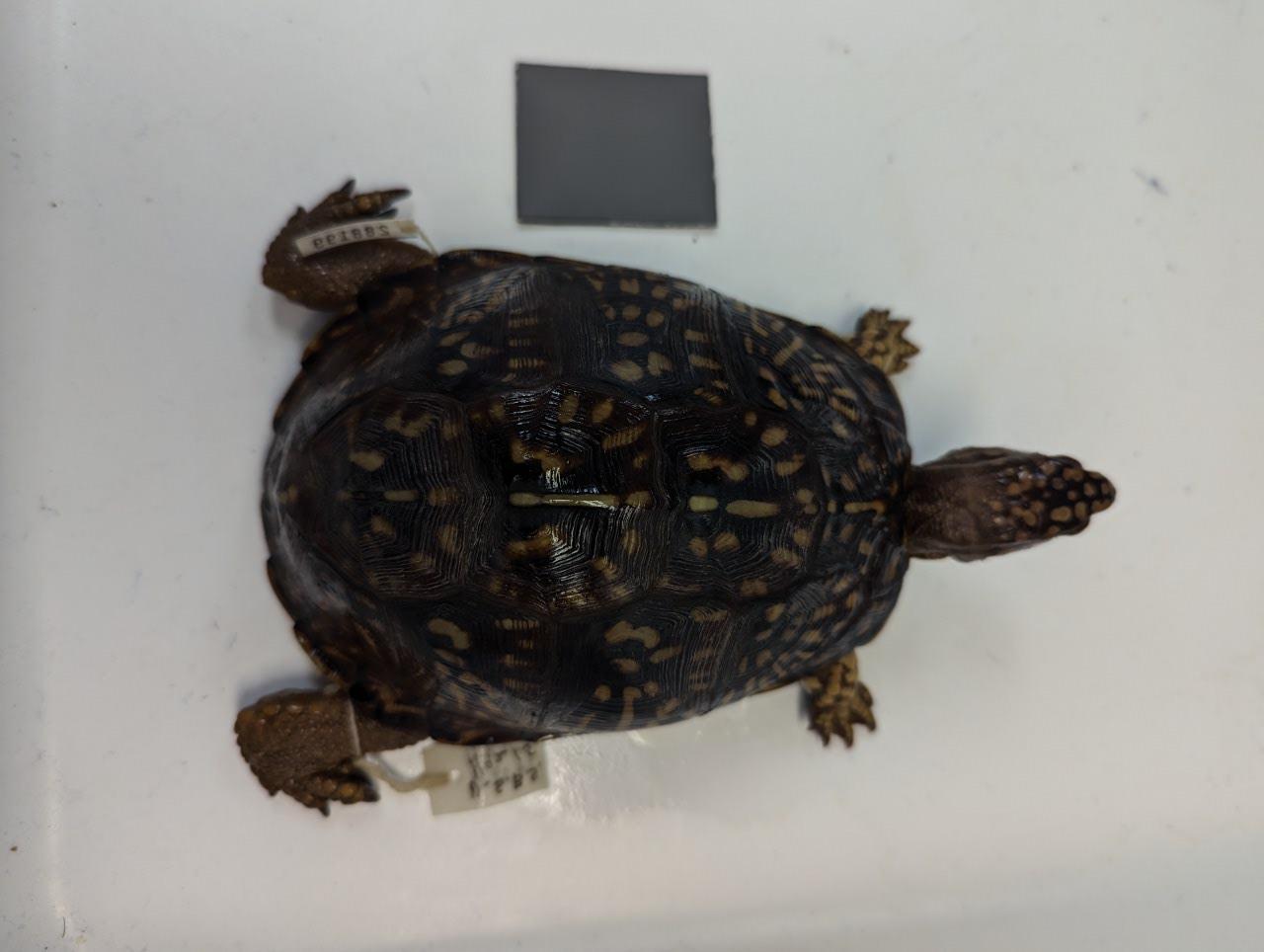

Supplement: Supplemental Information 6 — 98 photos of 98 turtles (single photo, all top view) that were used for the Citizen Science classification analysis. [file peerj-13-19690-s006.zip › TurtleClassification/TURTLE1034.jpg]

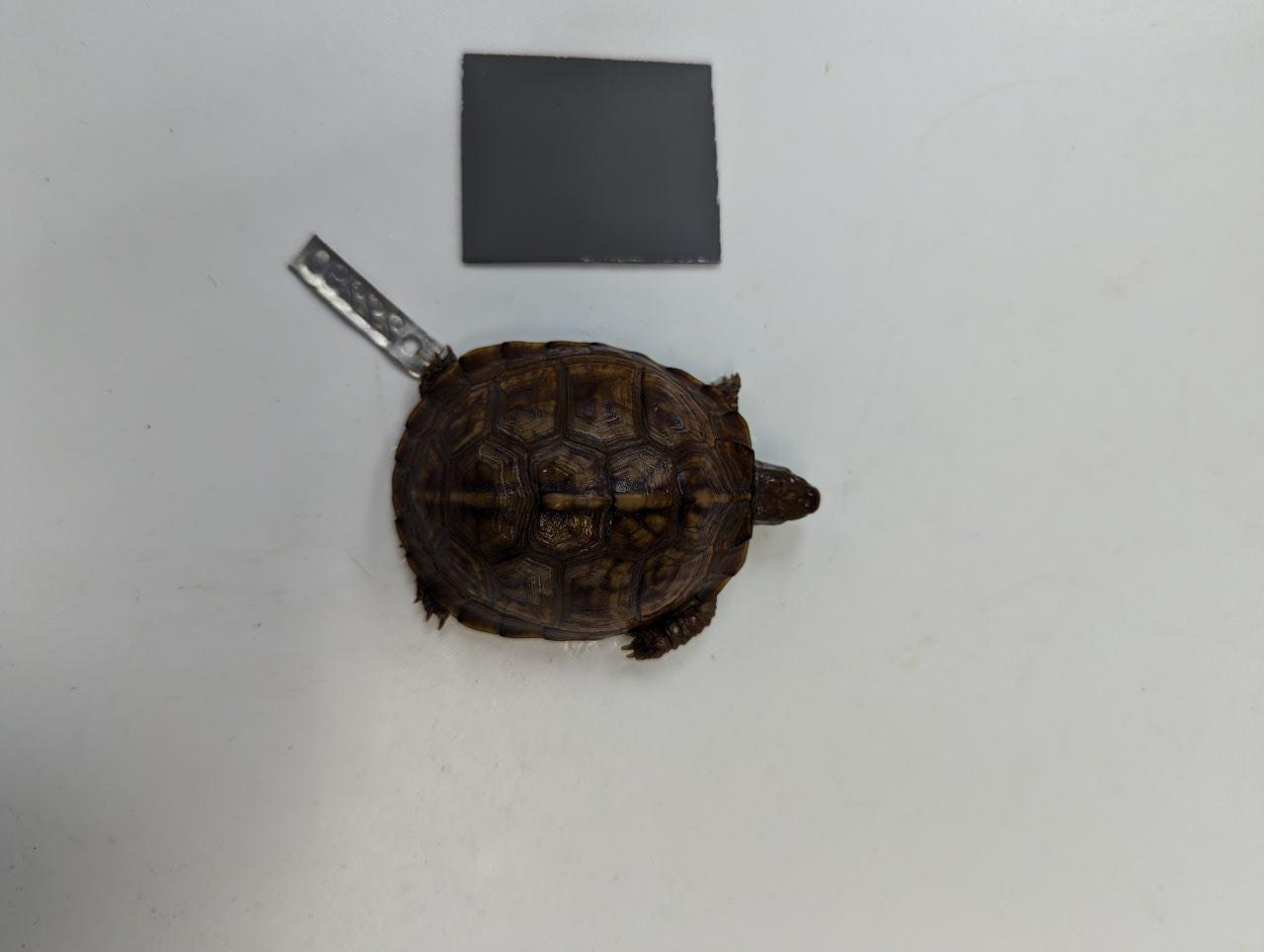

Supplement: Supplemental Information 6 — 98 photos of 98 turtles (single photo, all top view) that were used for the Citizen Science classification analysis. [file peerj-13-19690-s006.zip › TurtleClassification/TURTLE1035.jpg]

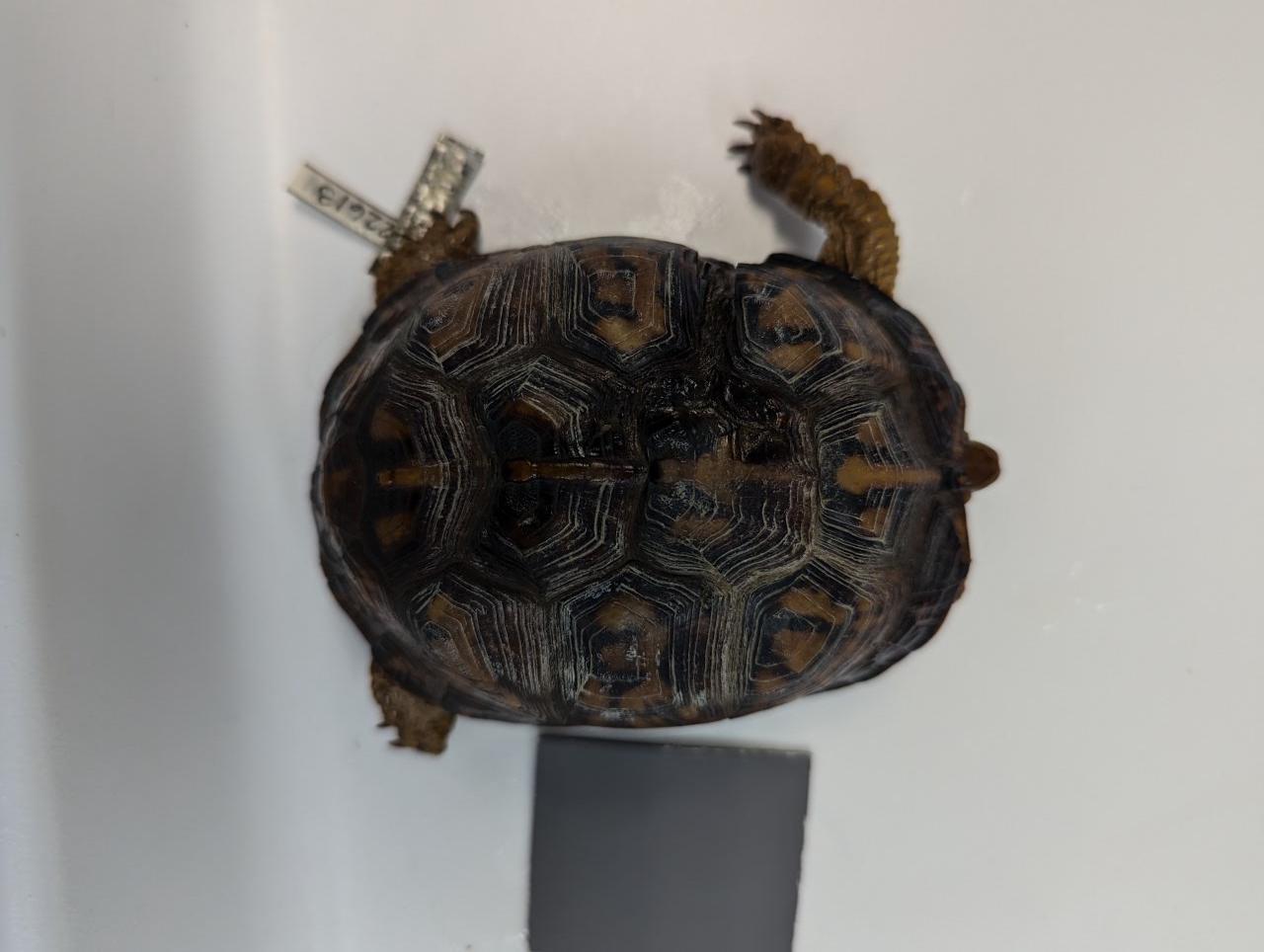

Supplement: Supplemental Information 6 — 98 photos of 98 turtles (single photo, all top view) that were used for the Citizen Science classification analysis. [file peerj-13-19690-s006.zip › TurtleClassification/TURTLE1036.jpg]

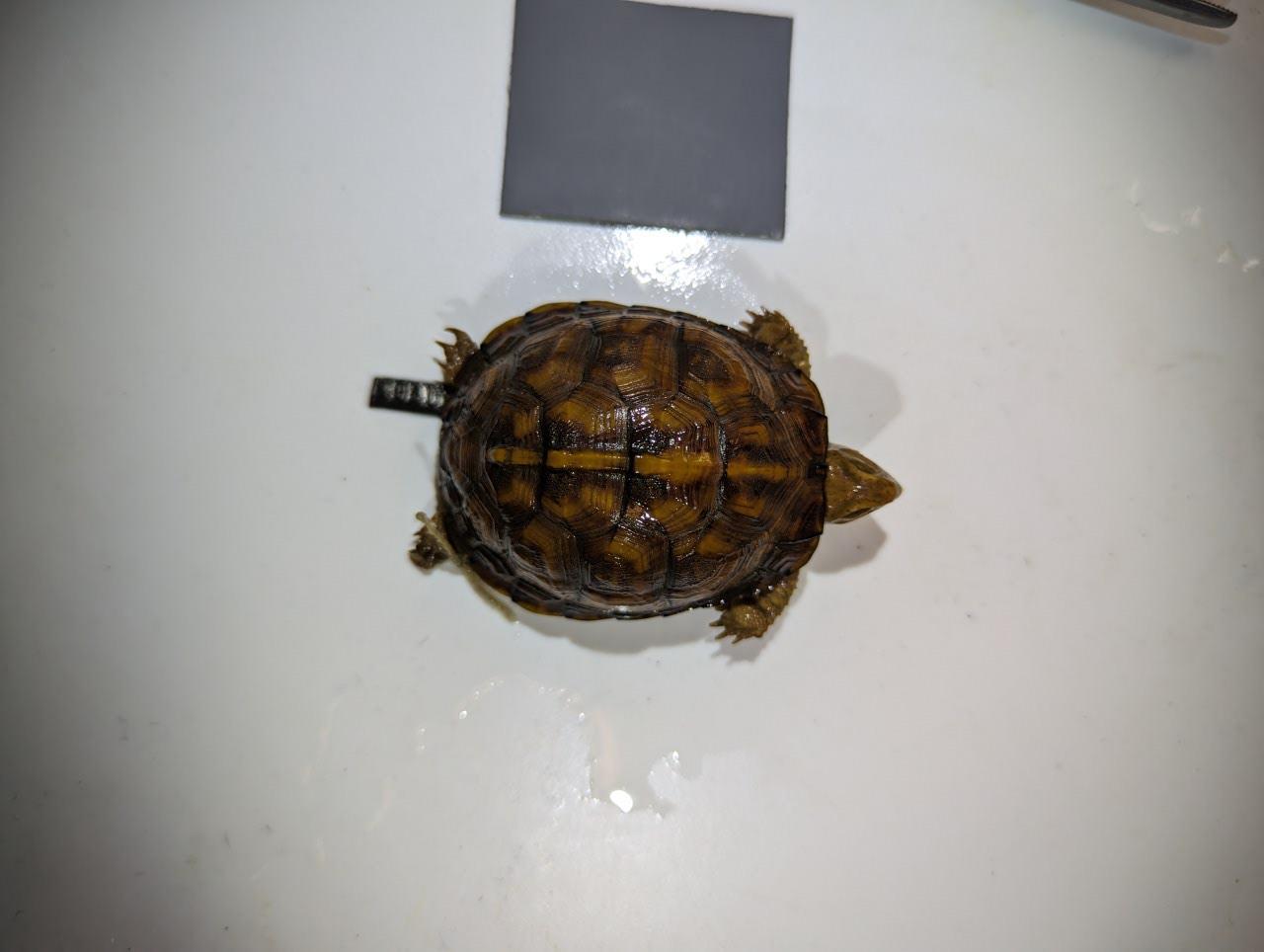

Supplement: Supplemental Information 6 — 98 photos of 98 turtles (single photo, all top view) that were used for the Citizen Science classification analysis. [file peerj-13-19690-s006.zip › TurtleClassification/TURTLE1037.jpg]

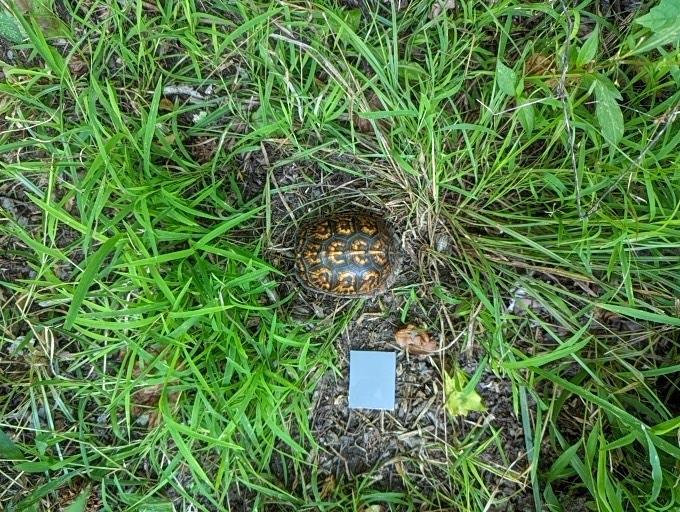

Supplement: Supplemental Information 6 — 98 photos of 98 turtles (single photo, all top view) that were used for the Citizen Science classification analysis. [file peerj-13-19690-s006.zip › TurtleClassification/TURTLE1038.jpg]

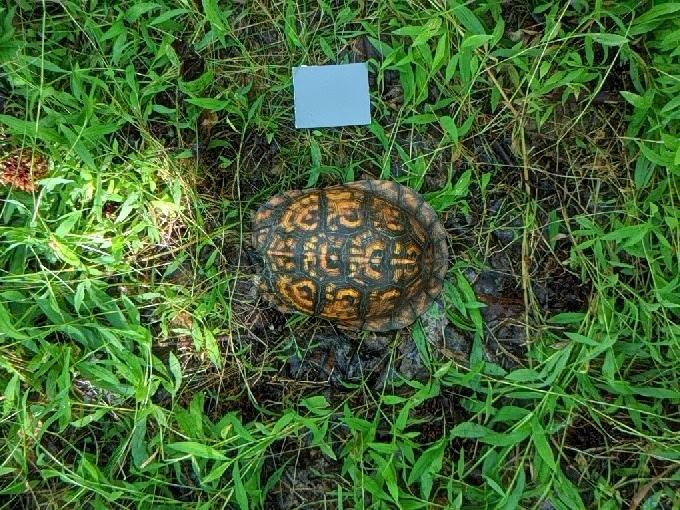

Supplement: Supplemental Information 6 — 98 photos of 98 turtles (single photo, all top view) that were used for the Citizen Science classification analysis. [file peerj-13-19690-s006.zip › TurtleClassification/TURTLE1039.jpg]

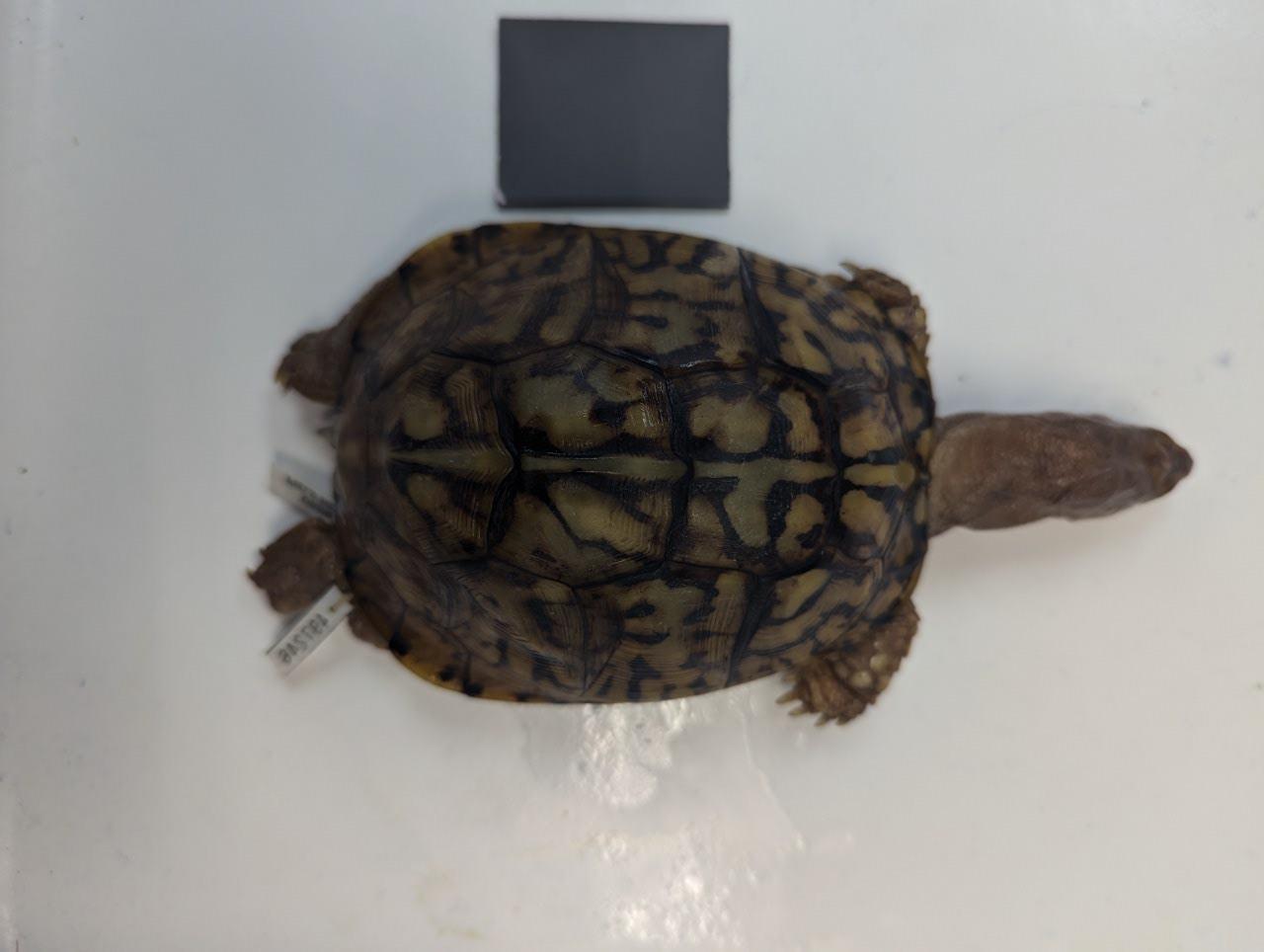

Supplement: Supplemental Information 6 — 98 photos of 98 turtles (single photo, all top view) that were used for the Citizen Science classification analysis. [file peerj-13-19690-s006.zip › TurtleClassification/TURTLE1040.jpg]

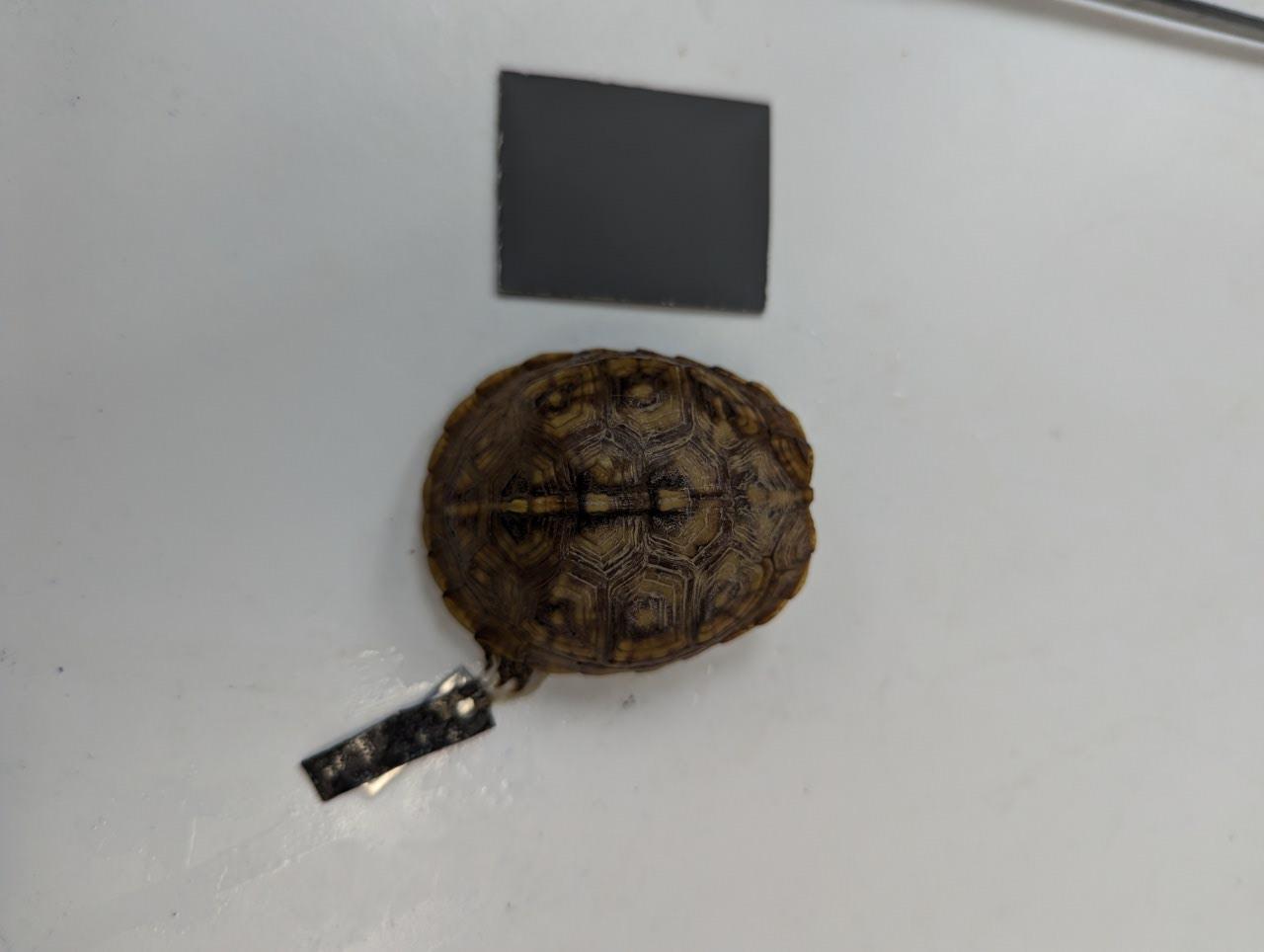

Supplement: Supplemental Information 6 — 98 photos of 98 turtles (single photo, all top view) that were used for the Citizen Science classification analysis. [file peerj-13-19690-s006.zip › TurtleClassification/TURTLE1041.jpg]

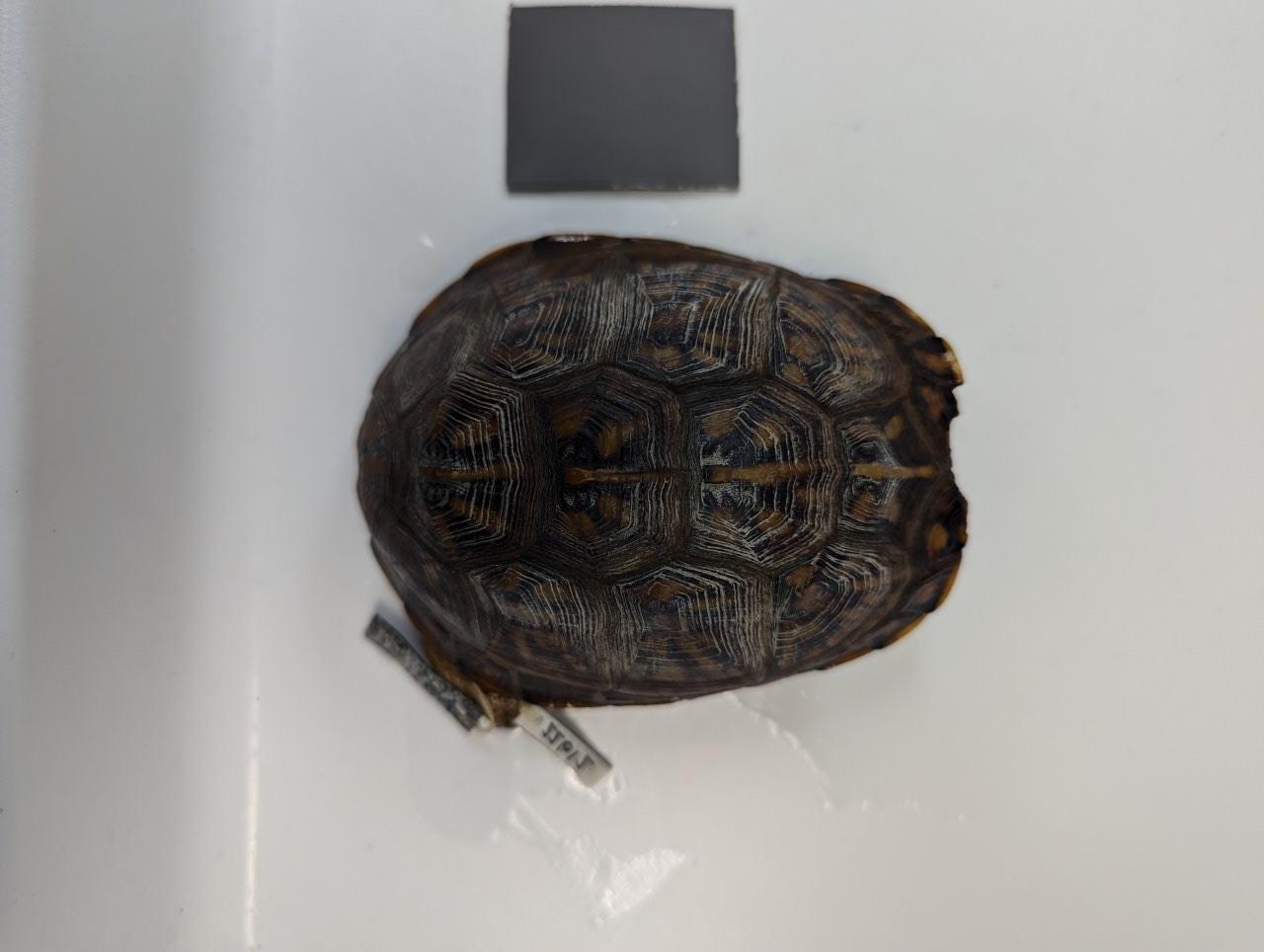

Supplement: Supplemental Information 6 — 98 photos of 98 turtles (single photo, all top view) that were used for the Citizen Science classification analysis. [file peerj-13-19690-s006.zip › TurtleClassification/TURTLE1042.jpg]

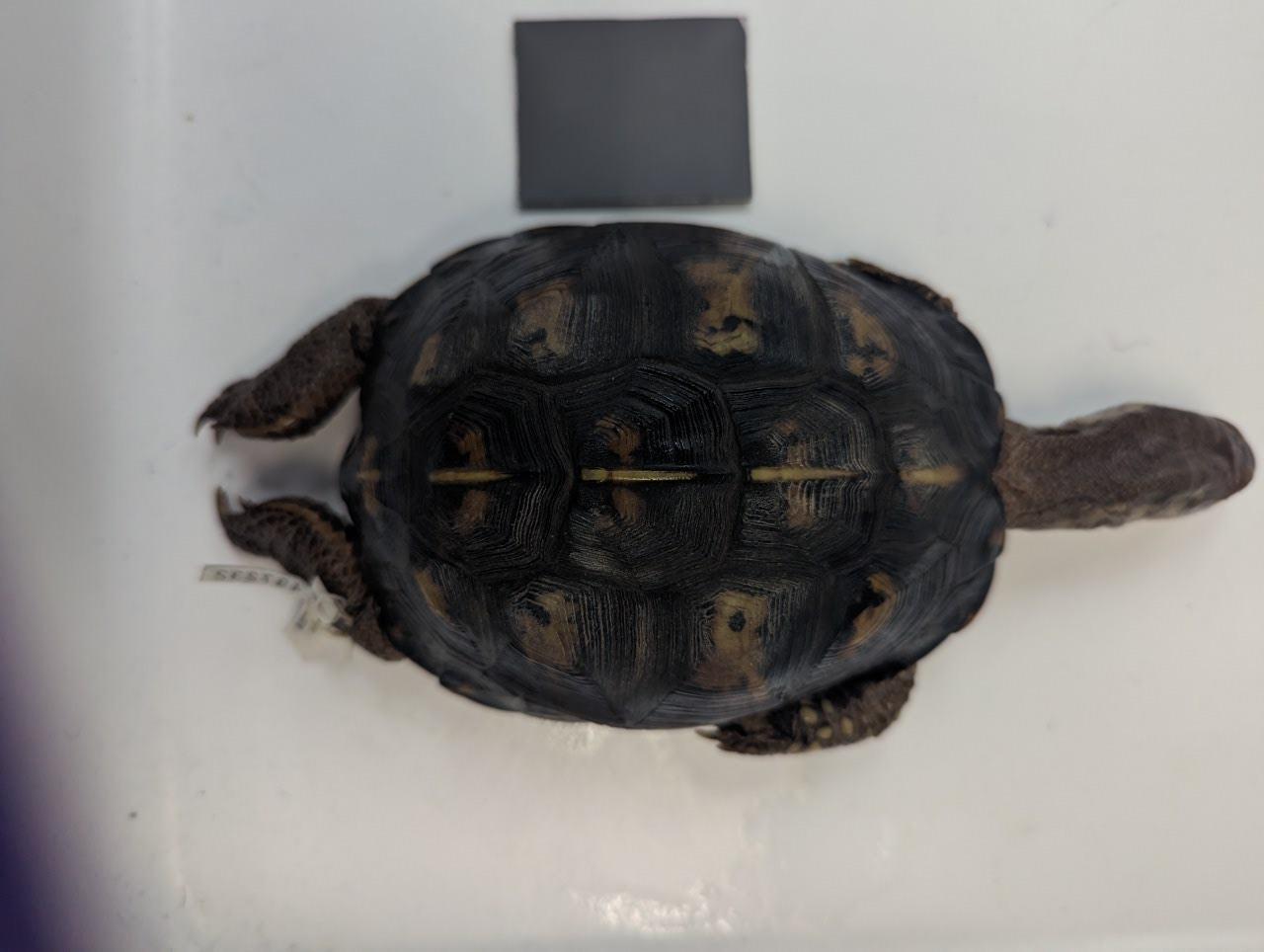

Supplement: Supplemental Information 6 — 98 photos of 98 turtles (single photo, all top view) that were used for the Citizen Science classification analysis. [file peerj-13-19690-s006.zip › TurtleClassification/TURTLE1043.jpg]

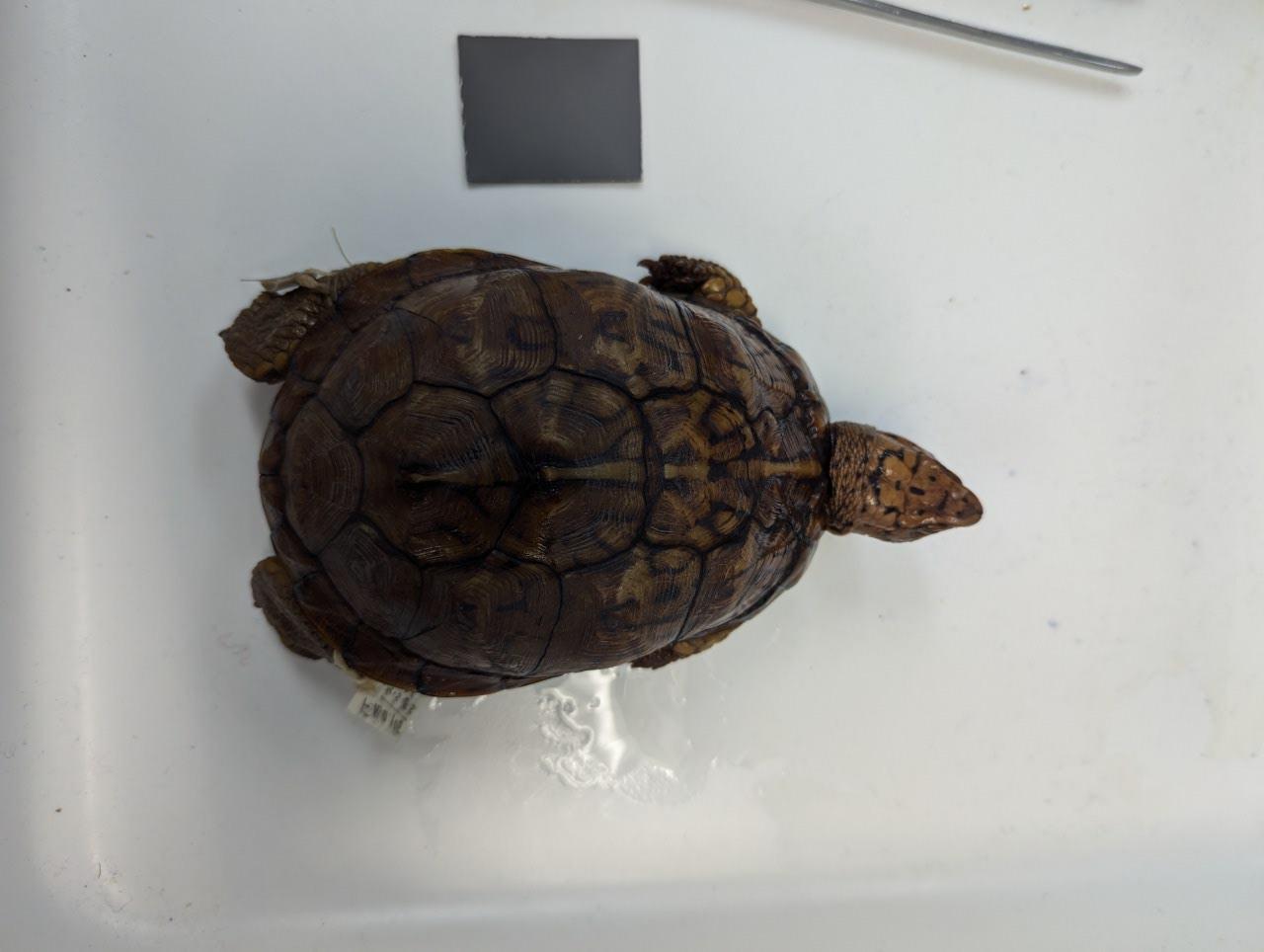

Supplement: Supplemental Information 6 — 98 photos of 98 turtles (single photo, all top view) that were used for the Citizen Science classification analysis. [file peerj-13-19690-s006.zip › TurtleClassification/TURTLE1044.jpg]

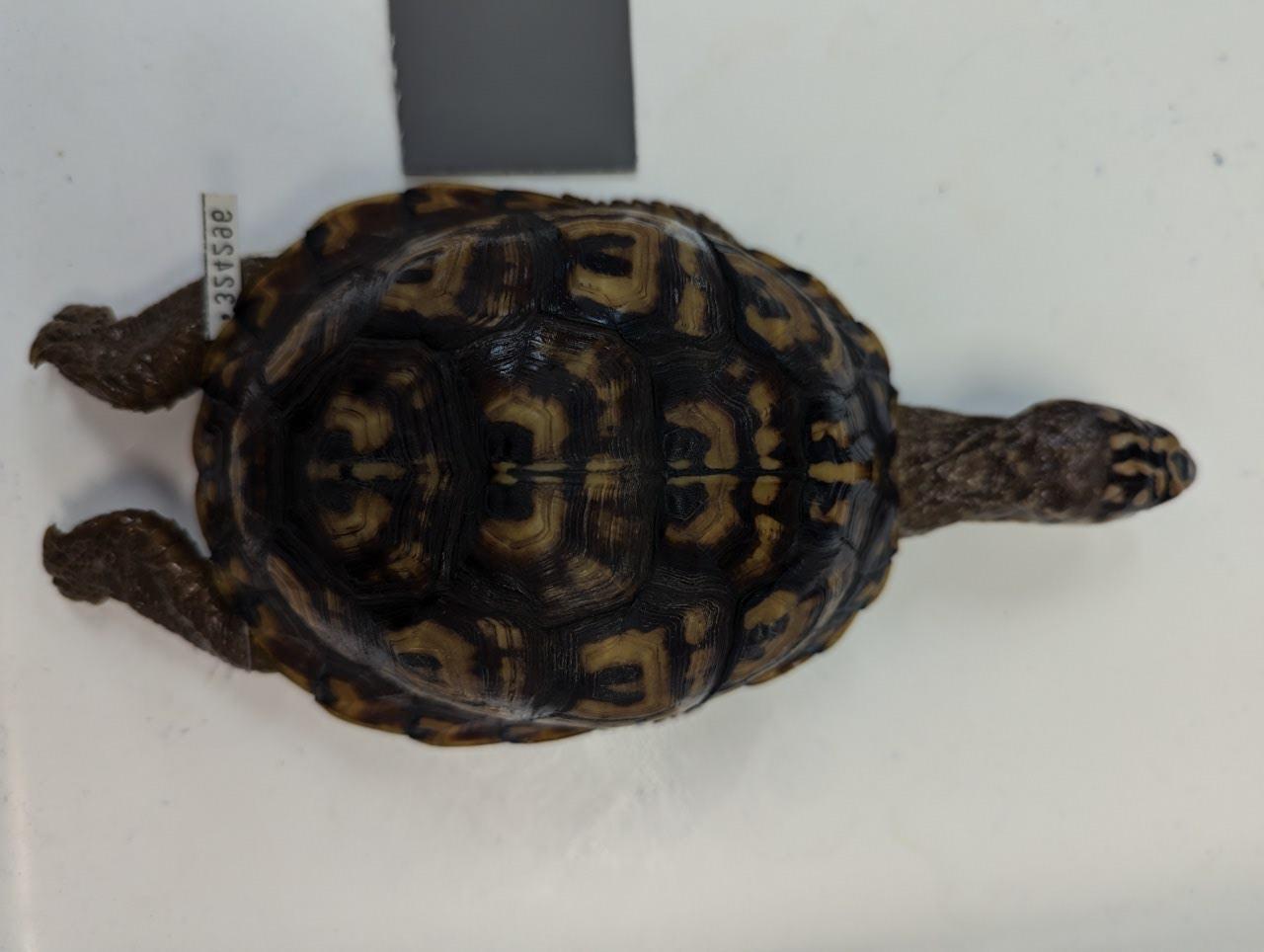

Supplement: Supplemental Information 6 — 98 photos of 98 turtles (single photo, all top view) that were used for the Citizen Science classification analysis. [file peerj-13-19690-s006.zip › TurtleClassification/TURTLE1045.jpg]

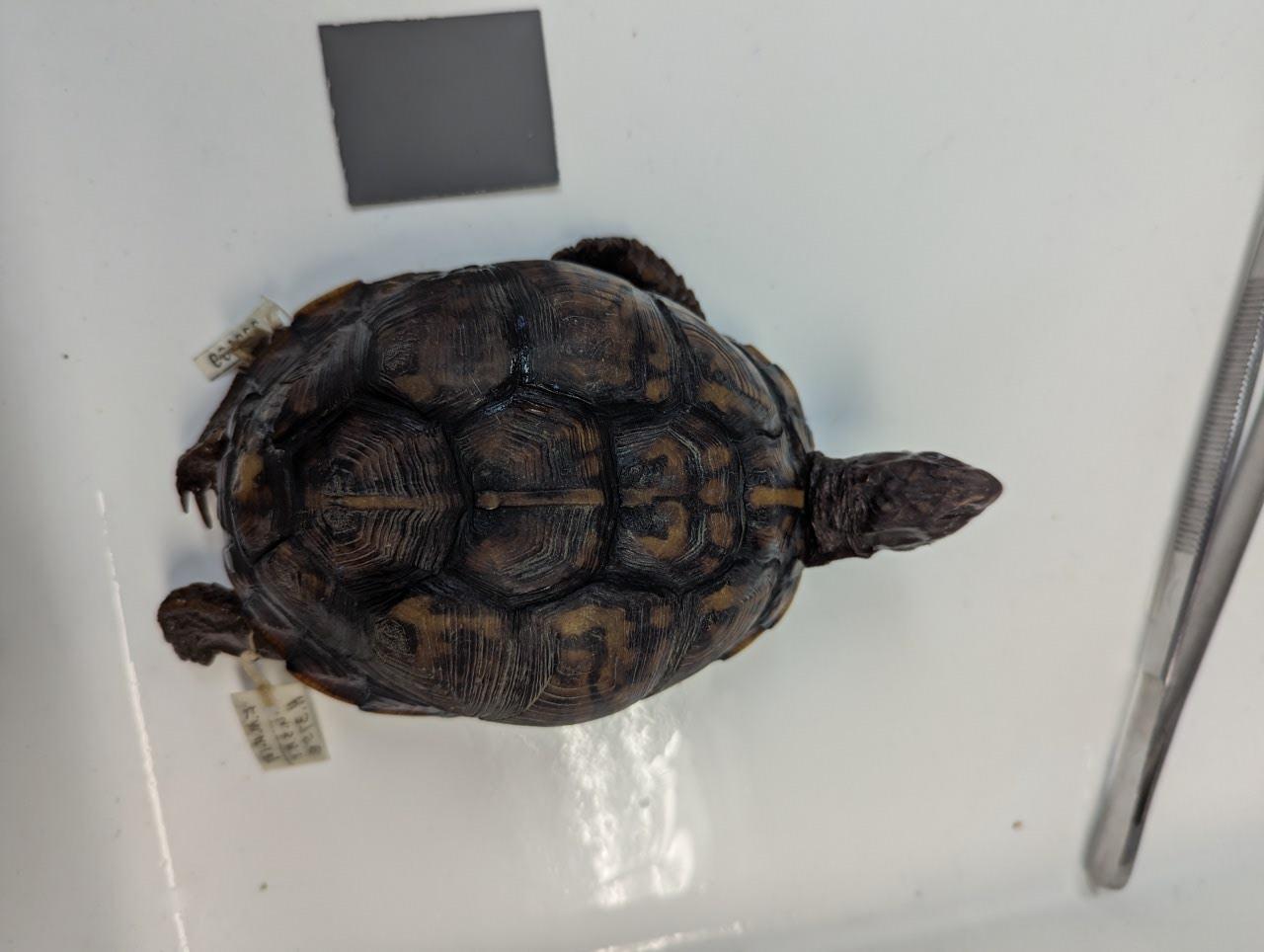

Supplement: Supplemental Information 6 — 98 photos of 98 turtles (single photo, all top view) that were used for the Citizen Science classification analysis. [file peerj-13-19690-s006.zip › TurtleClassification/TURTLE1046.jpg]

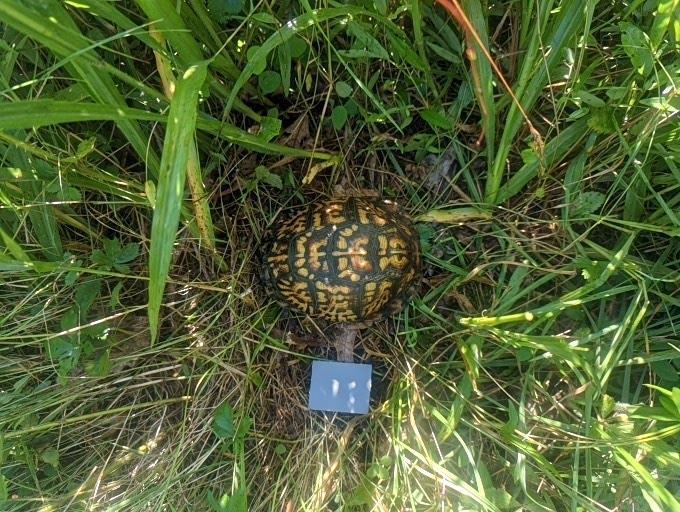

Supplement: Supplemental Information 6 — 98 photos of 98 turtles (single photo, all top view) that were used for the Citizen Science classification analysis. [file peerj-13-19690-s006.zip › TurtleClassification/TURTLE1047.jpg]

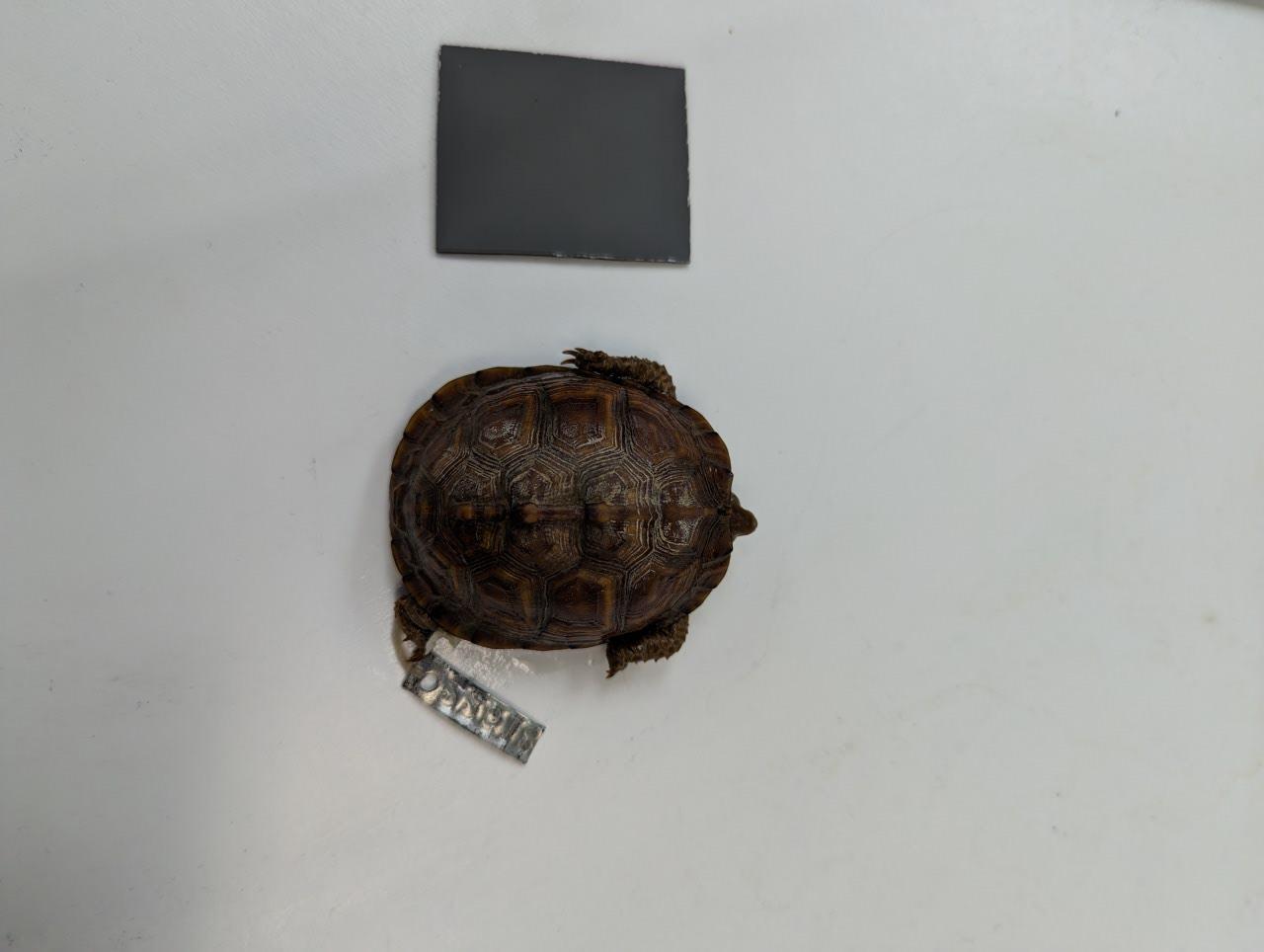

Supplement: Supplemental Information 6 — 98 photos of 98 turtles (single photo, all top view) that were used for the Citizen Science classification analysis. [file peerj-13-19690-s006.zip › TurtleClassification/TURTLE1048.jpg]

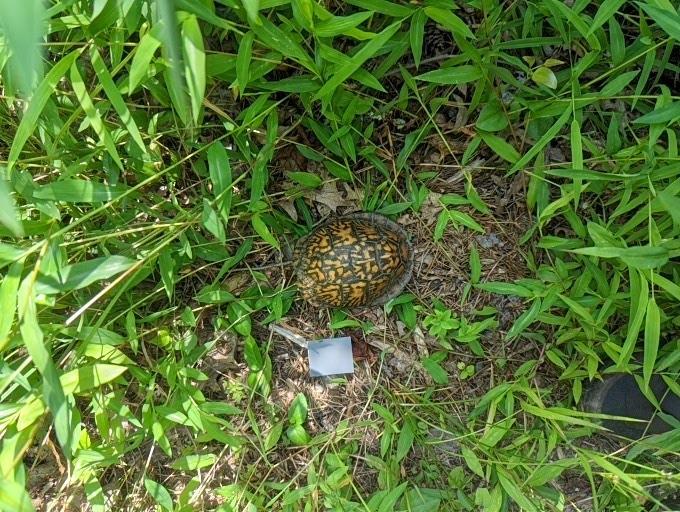

Supplement: Supplemental Information 6 — 98 photos of 98 turtles (single photo, all top view) that were used for the Citizen Science classification analysis. [file peerj-13-19690-s006.zip › TurtleClassification/TURTLE1049.jpg]

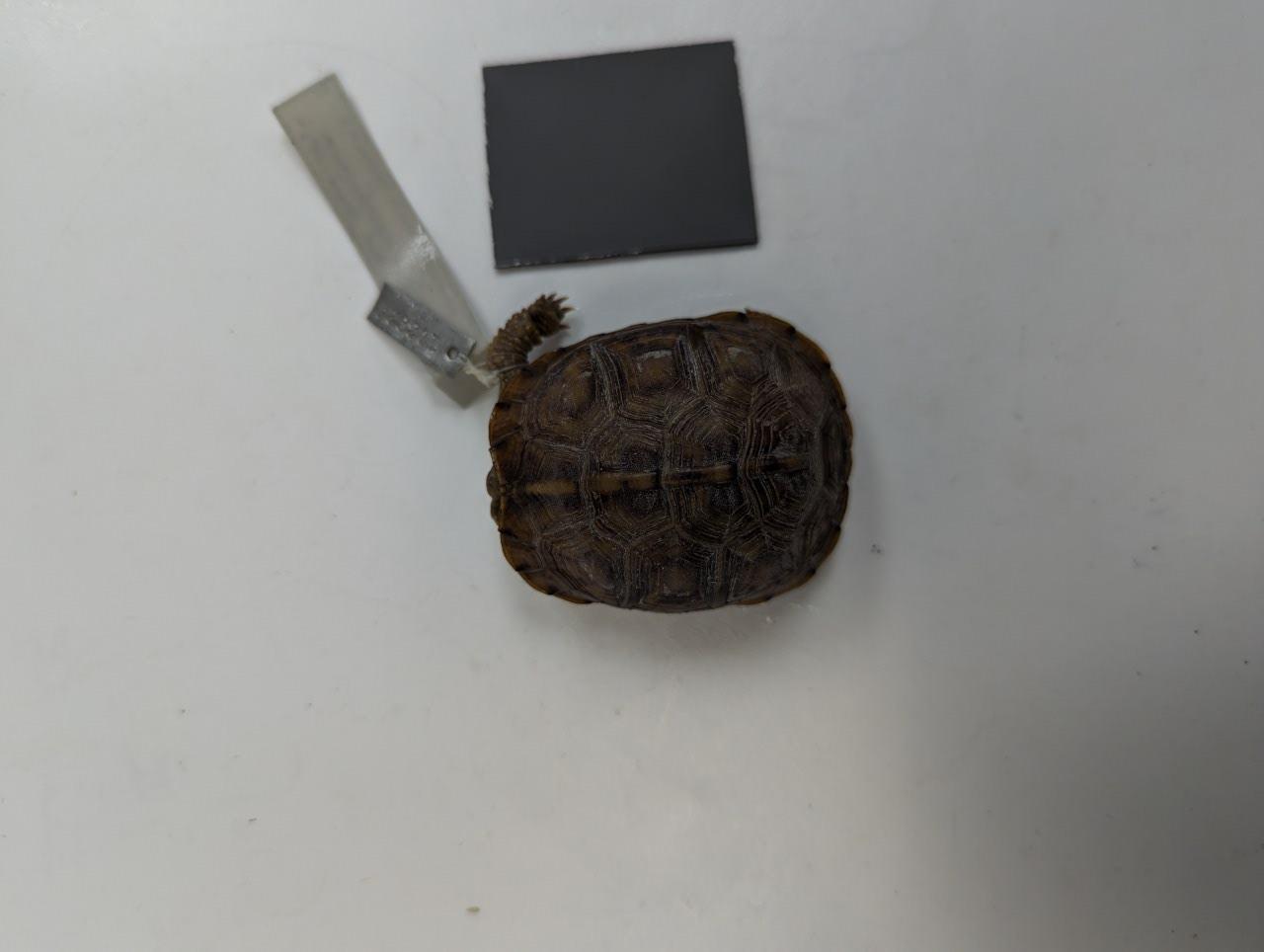

Supplement: Supplemental Information 6 — 98 photos of 98 turtles (single photo, all top view) that were used for the Citizen Science classification analysis. [file peerj-13-19690-s006.zip › TurtleClassification/TURTLE1050.jpg]

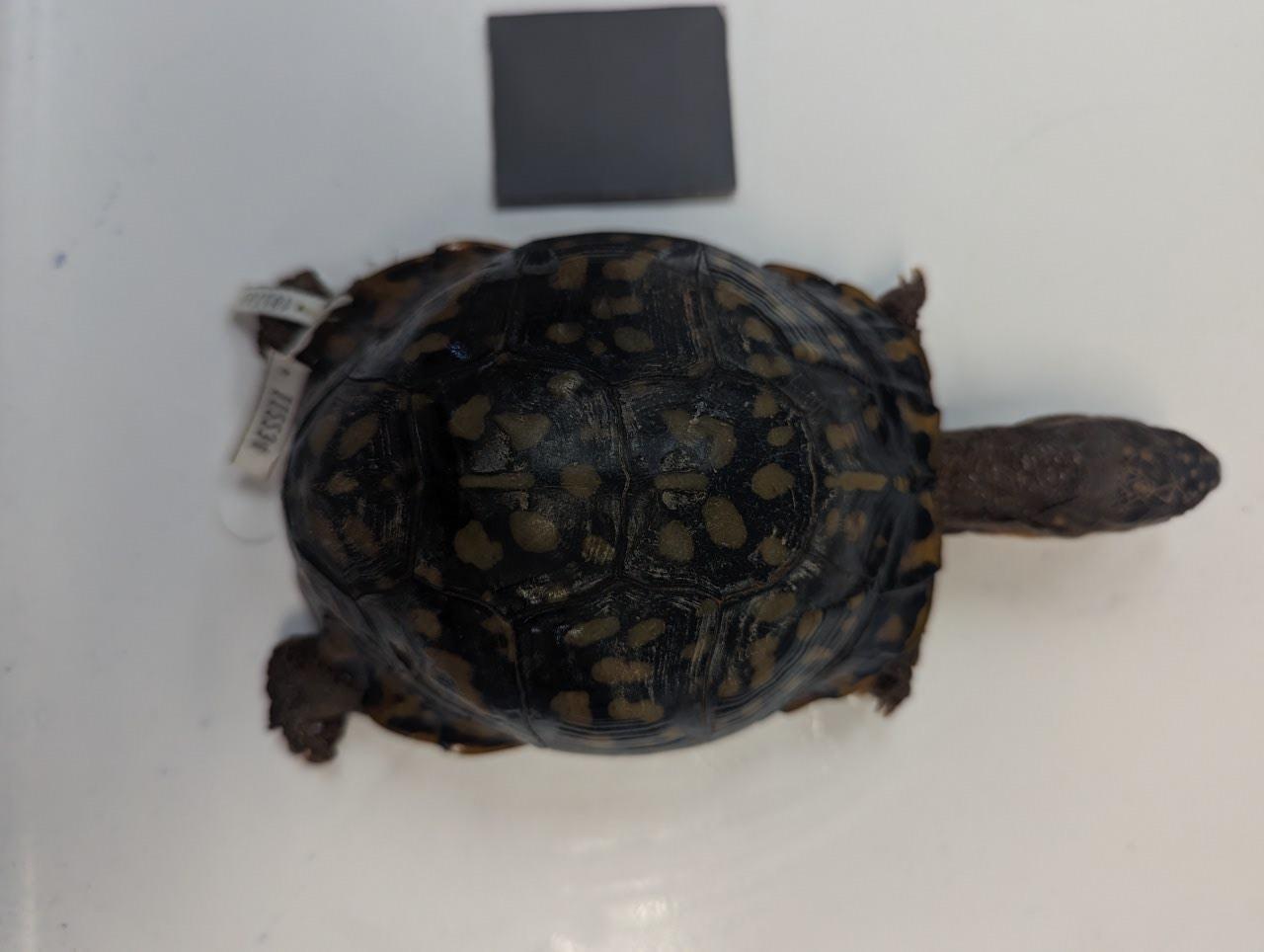

Supplement: Supplemental Information 6 — 98 photos of 98 turtles (single photo, all top view) that were used for the Citizen Science classification analysis. [file peerj-13-19690-s006.zip › TurtleClassification/TURTLE1051.jpg]

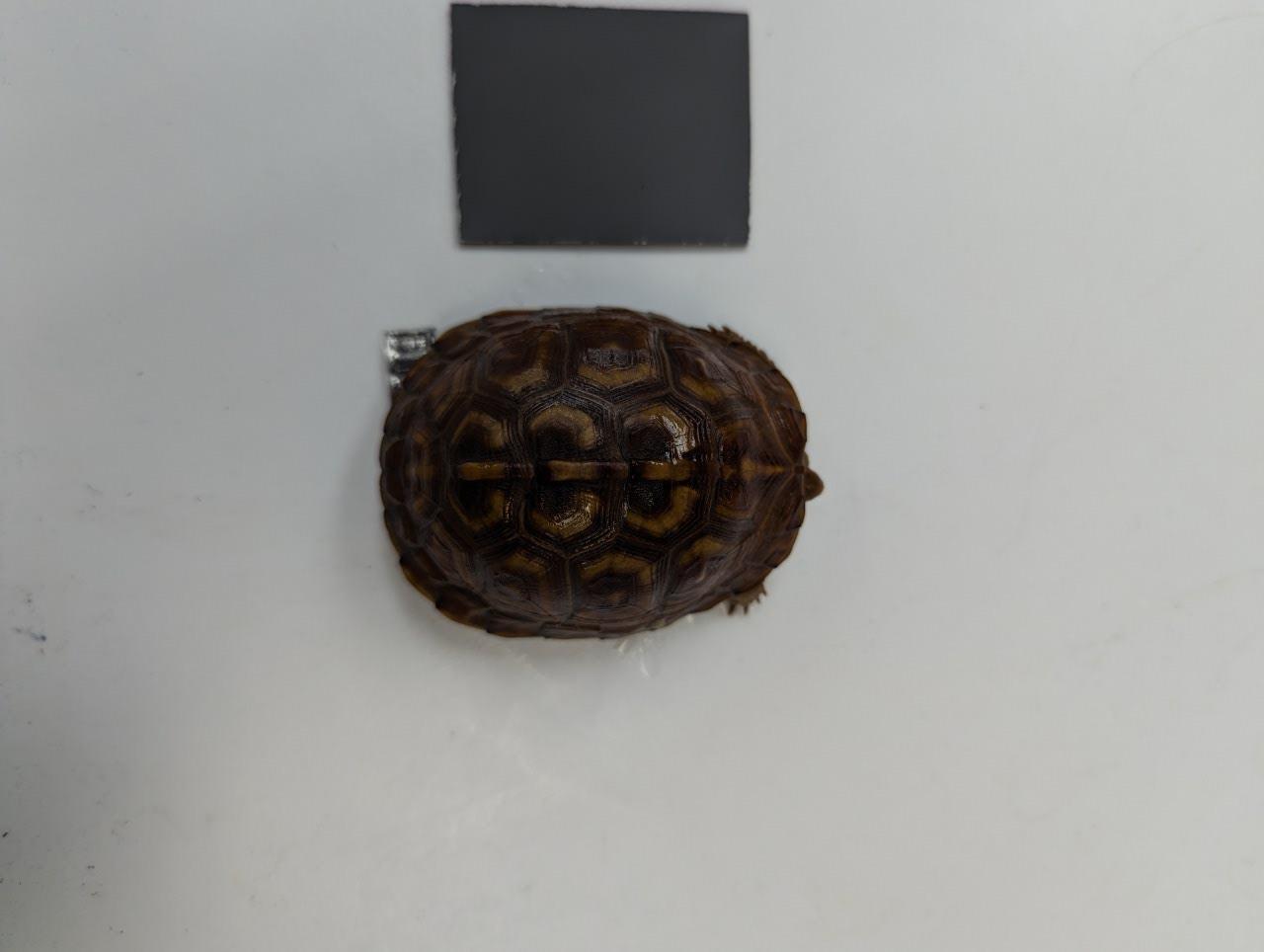

Supplement: Supplemental Information 6 — 98 photos of 98 turtles (single photo, all top view) that were used for the Citizen Science classification analysis. [file peerj-13-19690-s006.zip › TurtleClassification/TURTLE1052.jpg]

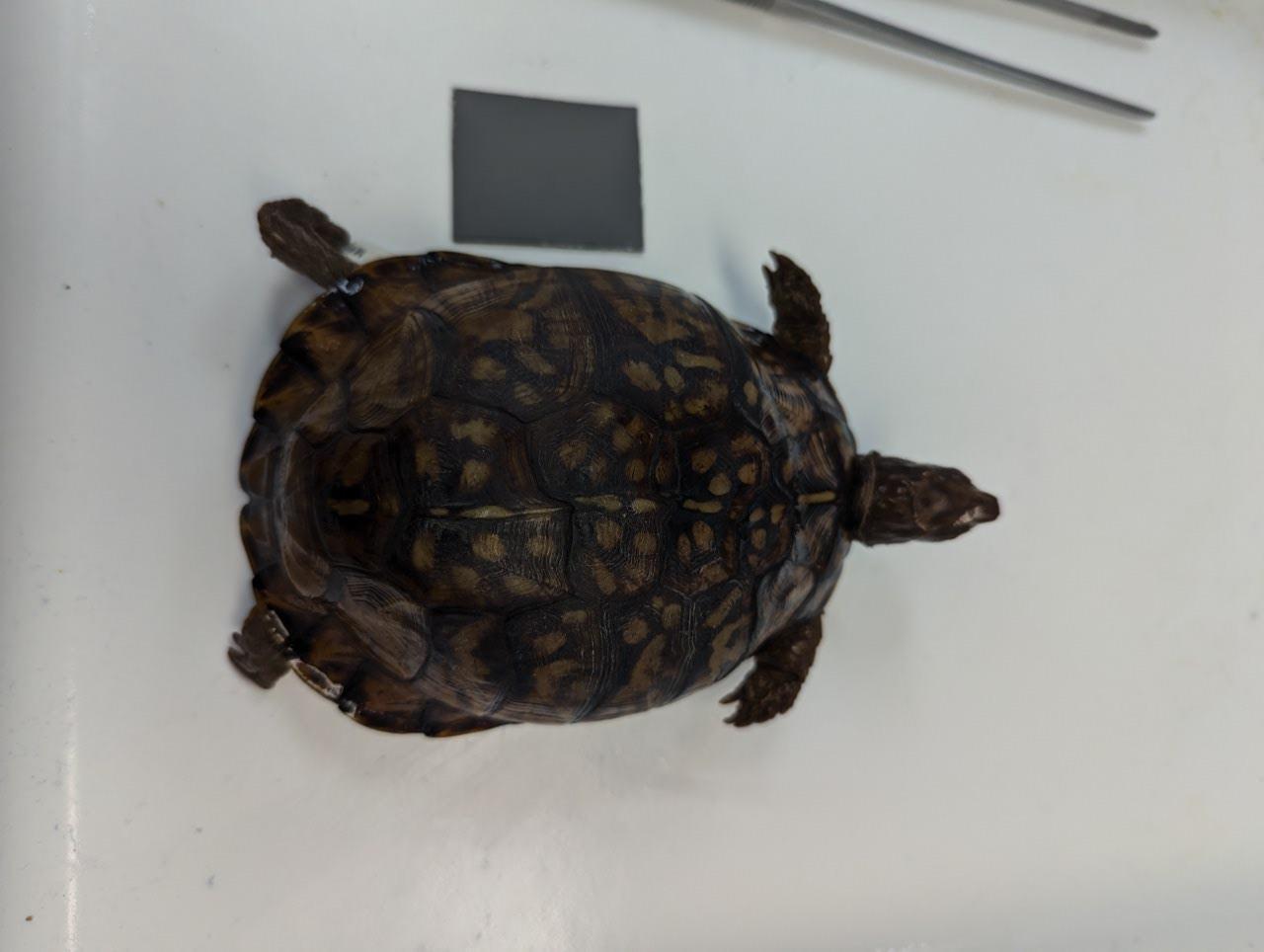

Supplement: Supplemental Information 6 — 98 photos of 98 turtles (single photo, all top view) that were used for the Citizen Science classification analysis. [file peerj-13-19690-s006.zip › TurtleClassification/TURTLE1053.jpg]

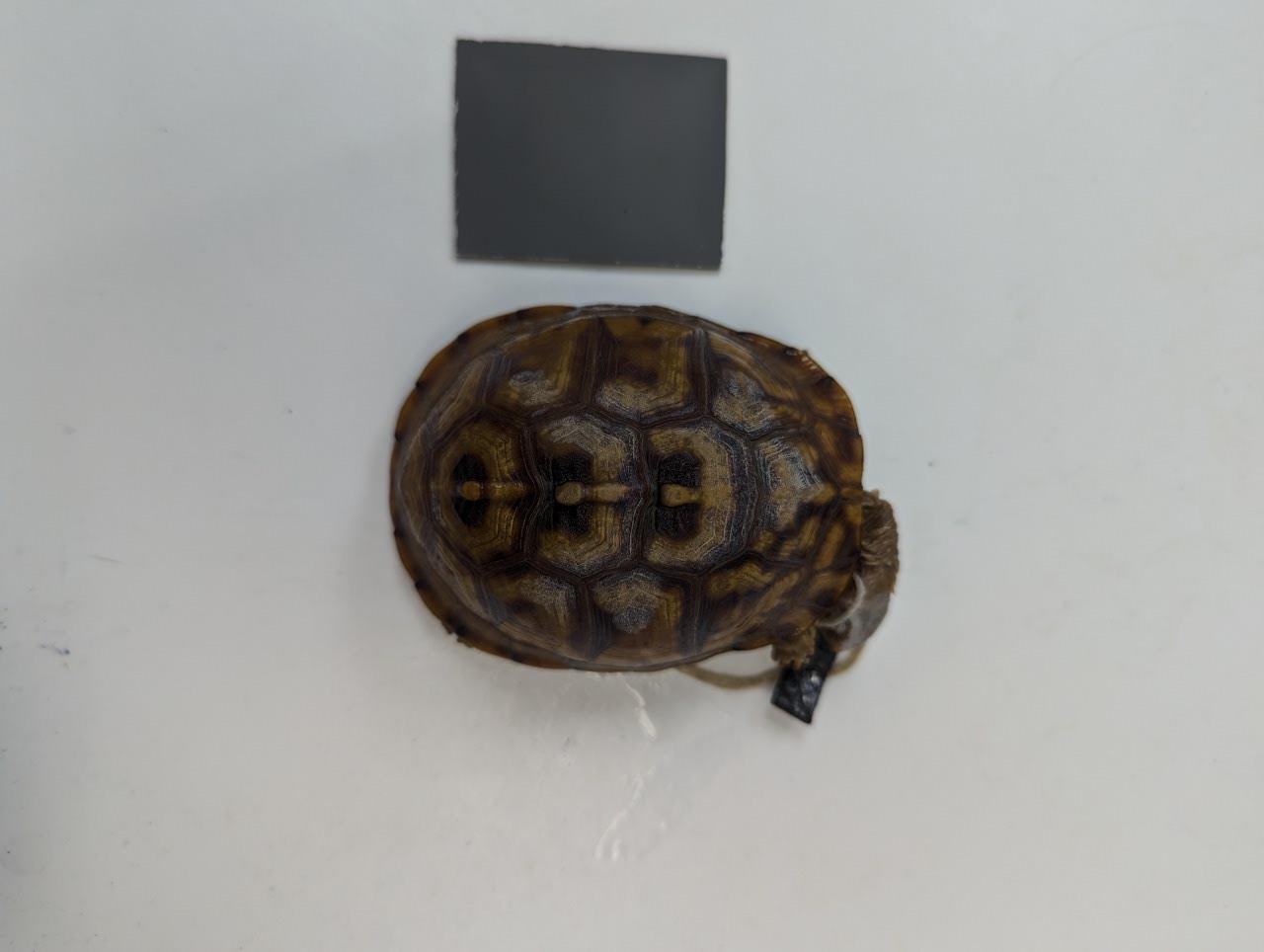

Supplement: Supplemental Information 6 — 98 photos of 98 turtles (single photo, all top view) that were used for the Citizen Science classification analysis. [file peerj-13-19690-s006.zip › TurtleClassification/TURTLE1054.jpg]

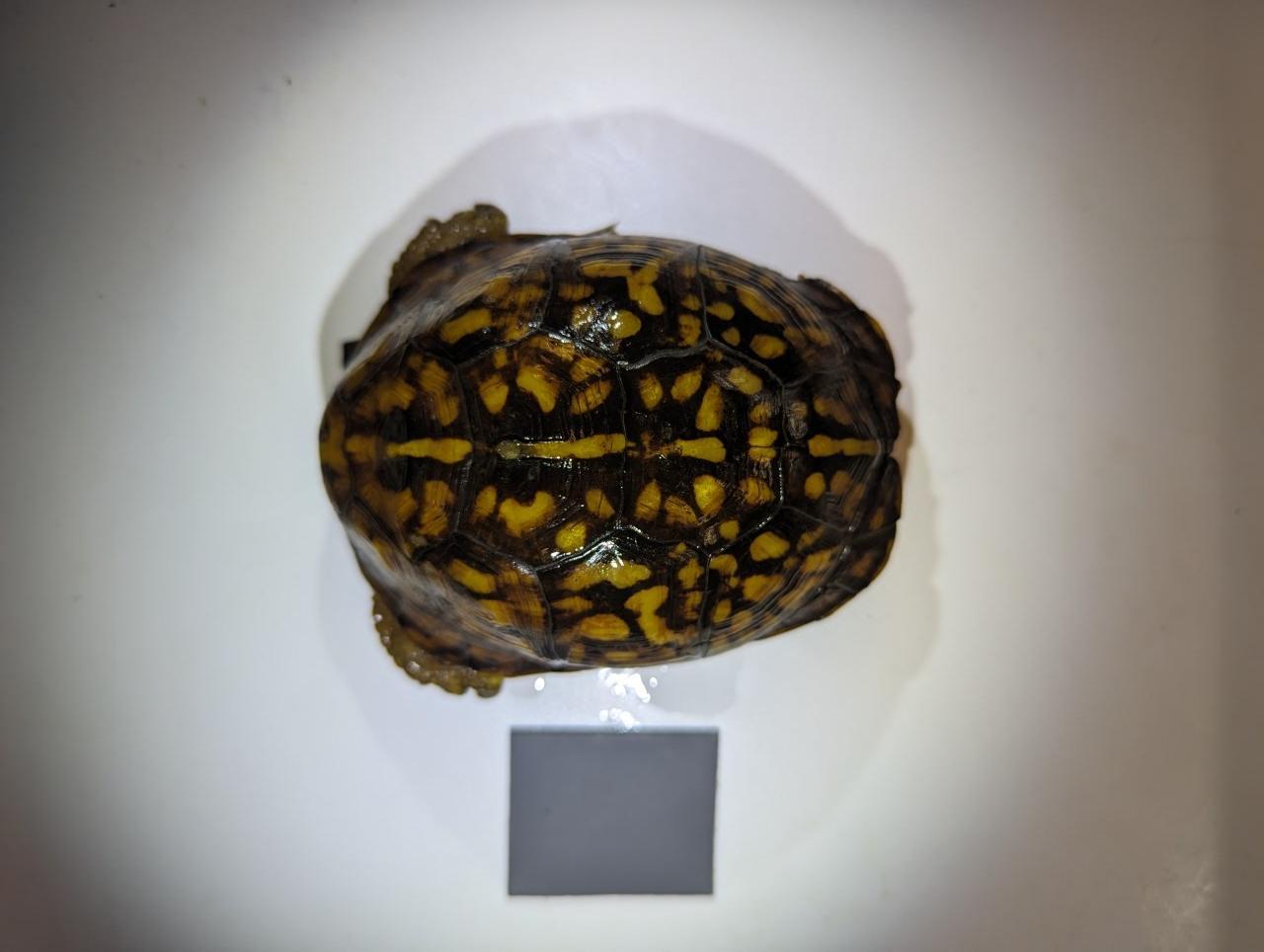

Supplement: Supplemental Information 6 — 98 photos of 98 turtles (single photo, all top view) that were used for the Citizen Science classification analysis. [file peerj-13-19690-s006.zip › TurtleClassification/TURTLE1055.jpg]

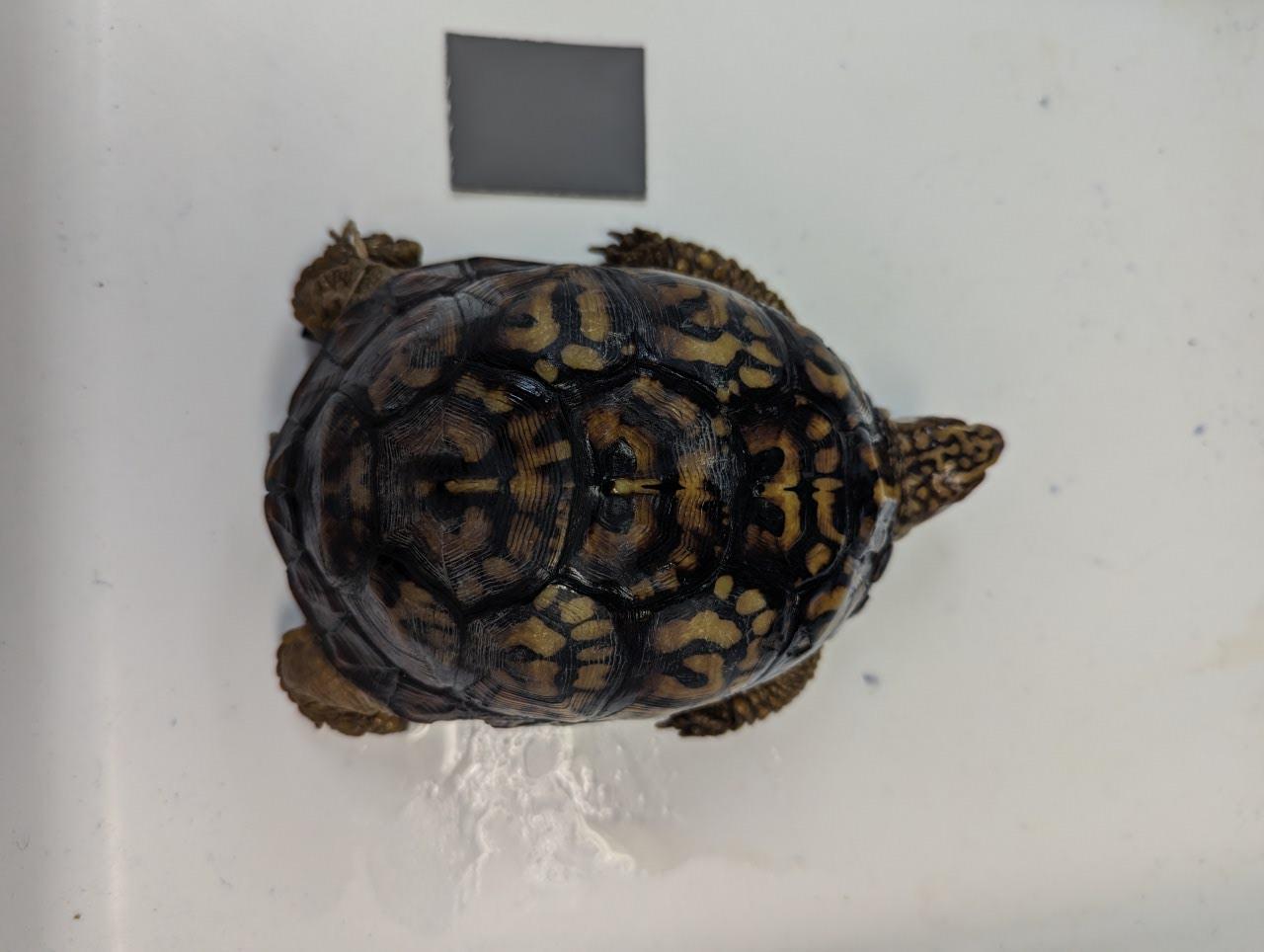

Supplement: Supplemental Information 6 — 98 photos of 98 turtles (single photo, all top view) that were used for the Citizen Science classification analysis. [file peerj-13-19690-s006.zip › TurtleClassification/TURTLE1056.jpg]

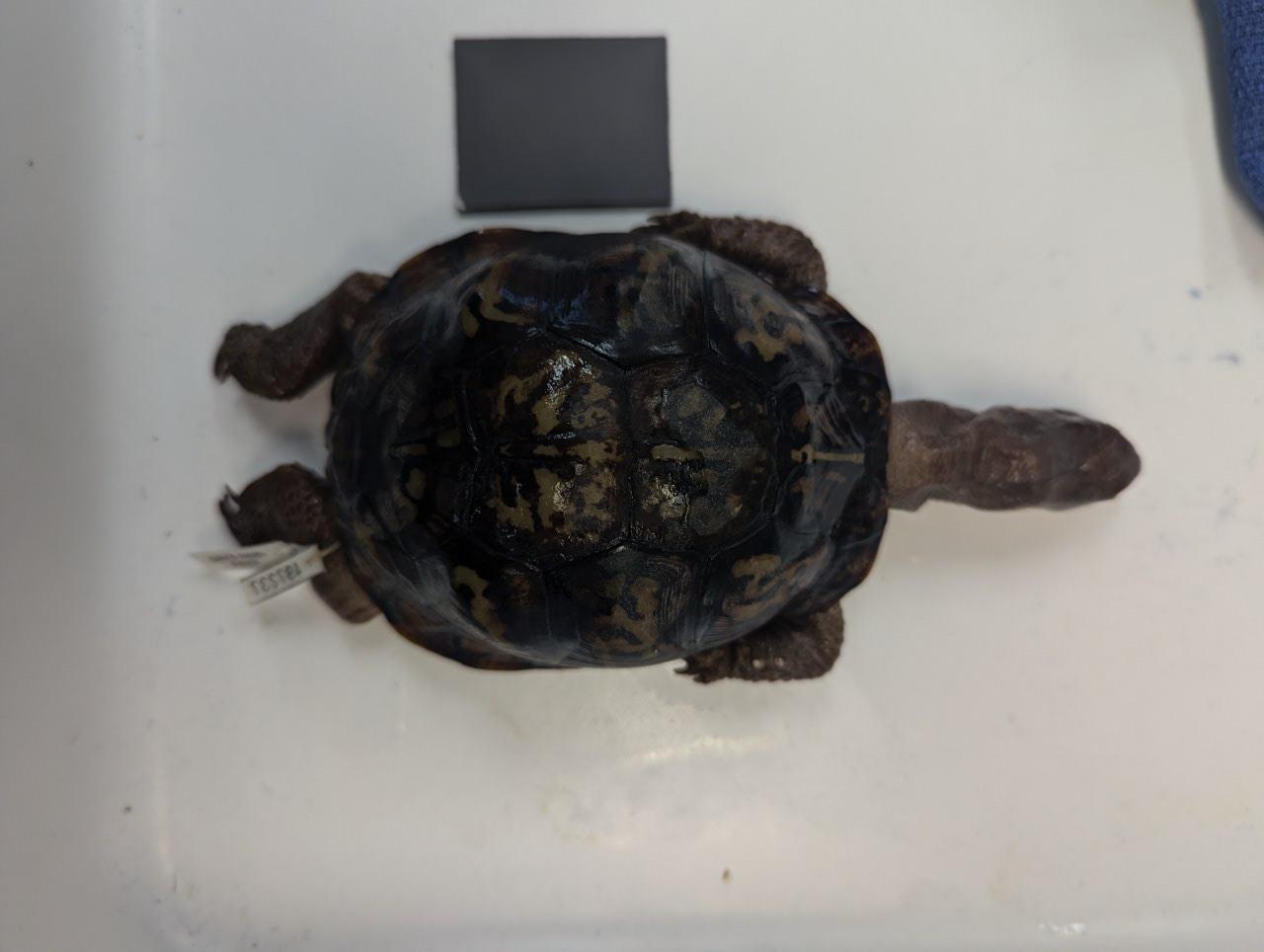

Supplement: Supplemental Information 6 — 98 photos of 98 turtles (single photo, all top view) that were used for the Citizen Science classification analysis. [file peerj-13-19690-s006.zip › TurtleClassification/TURTLE1057.jpg]

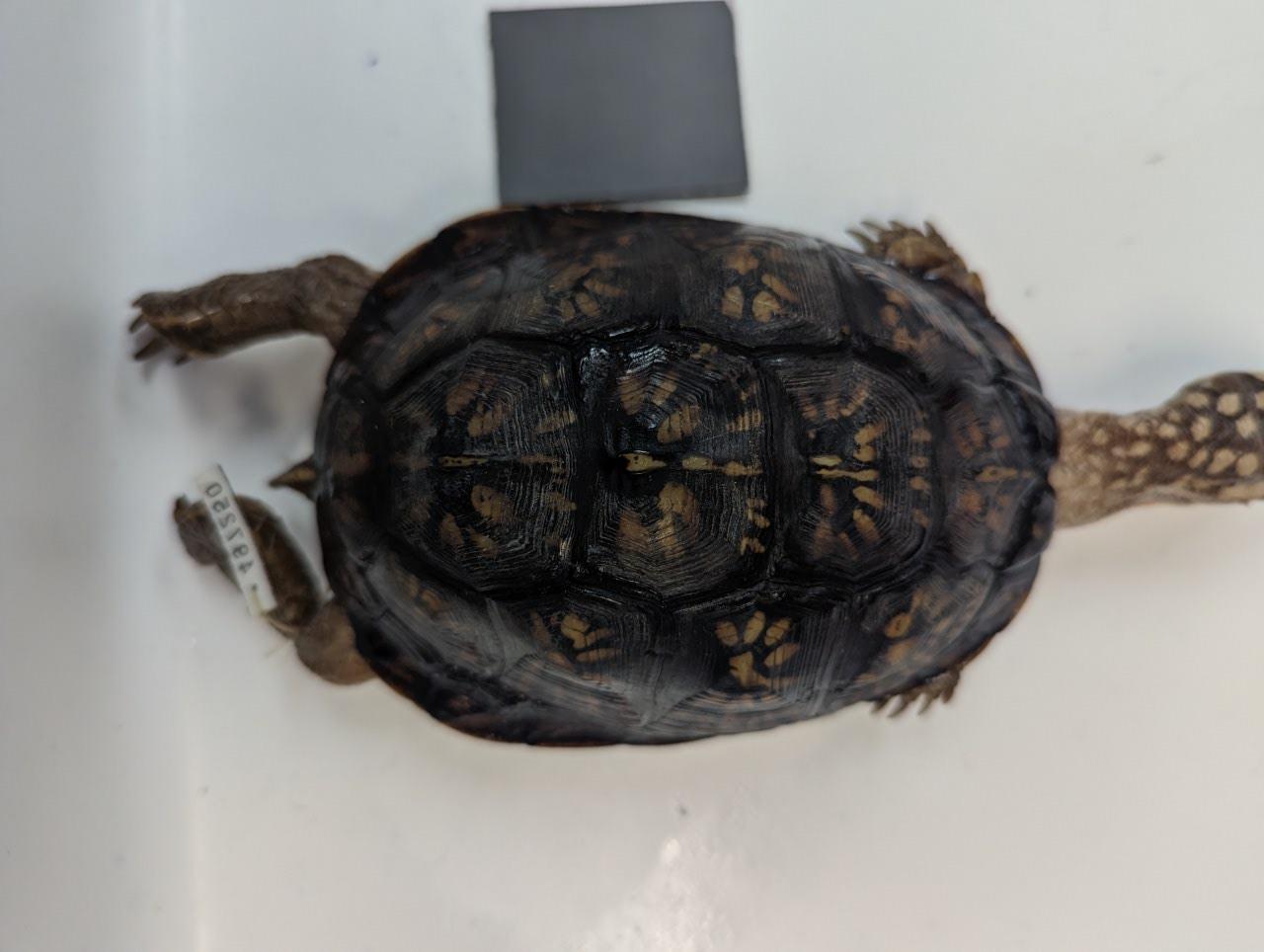

Supplement: Supplemental Information 6 — 98 photos of 98 turtles (single photo, all top view) that were used for the Citizen Science classification analysis. [file peerj-13-19690-s006.zip › TurtleClassification/TURTLE1059.jpg]

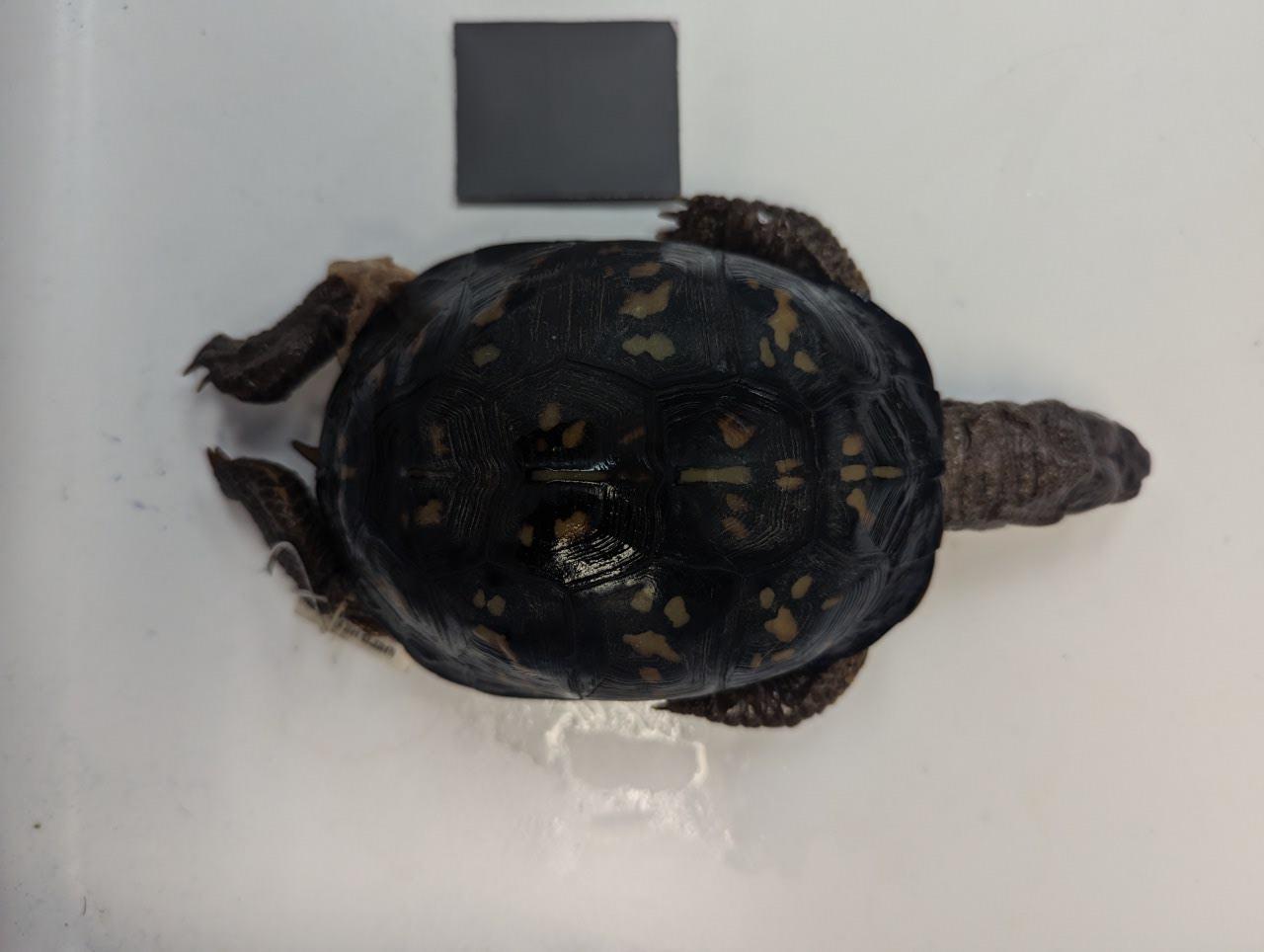

Supplement: Supplemental Information 6 — 98 photos of 98 turtles (single photo, all top view) that were used for the Citizen Science classification analysis. [file peerj-13-19690-s006.zip › TurtleClassification/TURTLE1060.jpg]

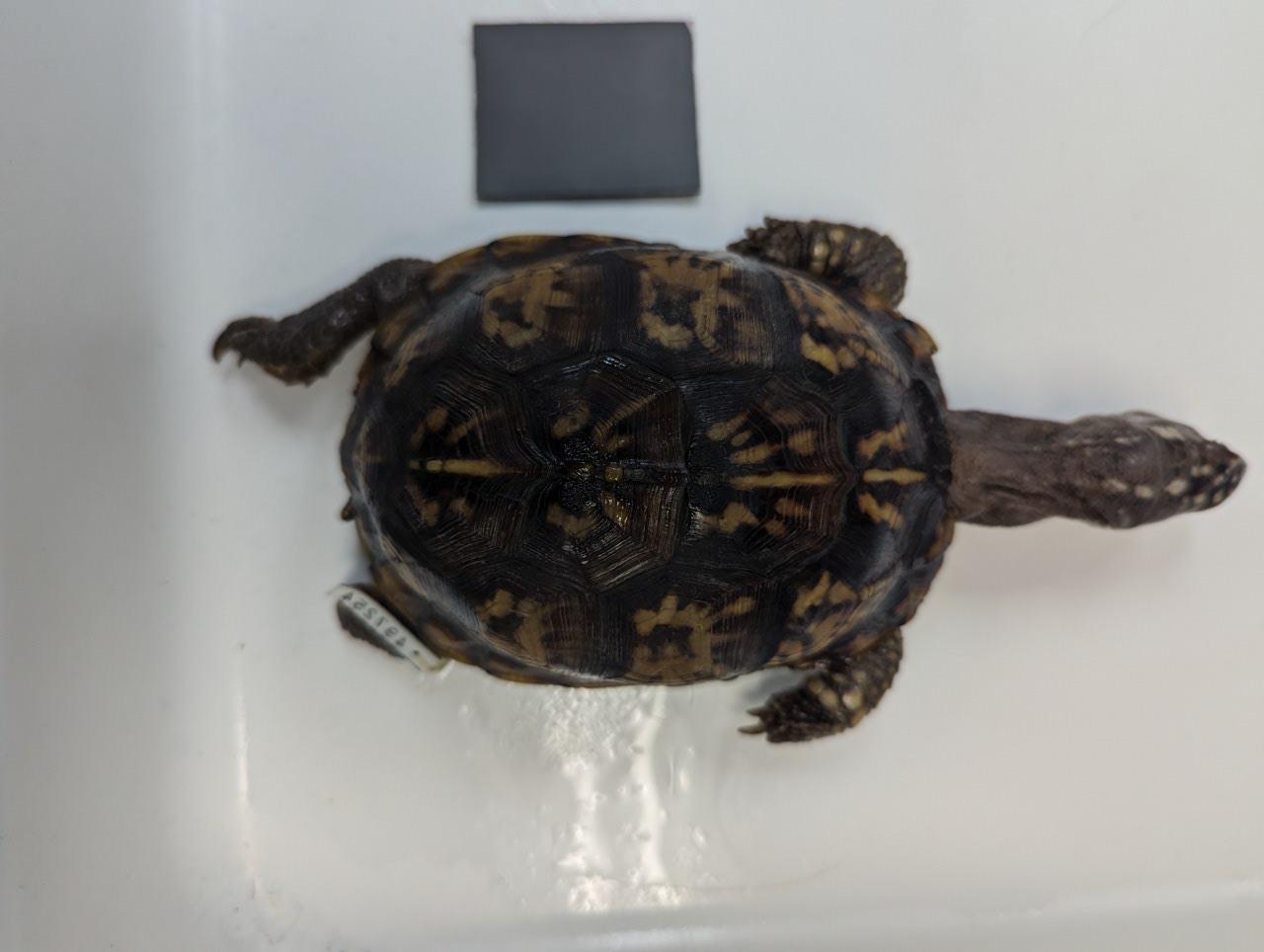

Supplement: Supplemental Information 6 — 98 photos of 98 turtles (single photo, all top view) that were used for the Citizen Science classification analysis. [file peerj-13-19690-s006.zip › TurtleClassification/TURTLE1061.jpg]

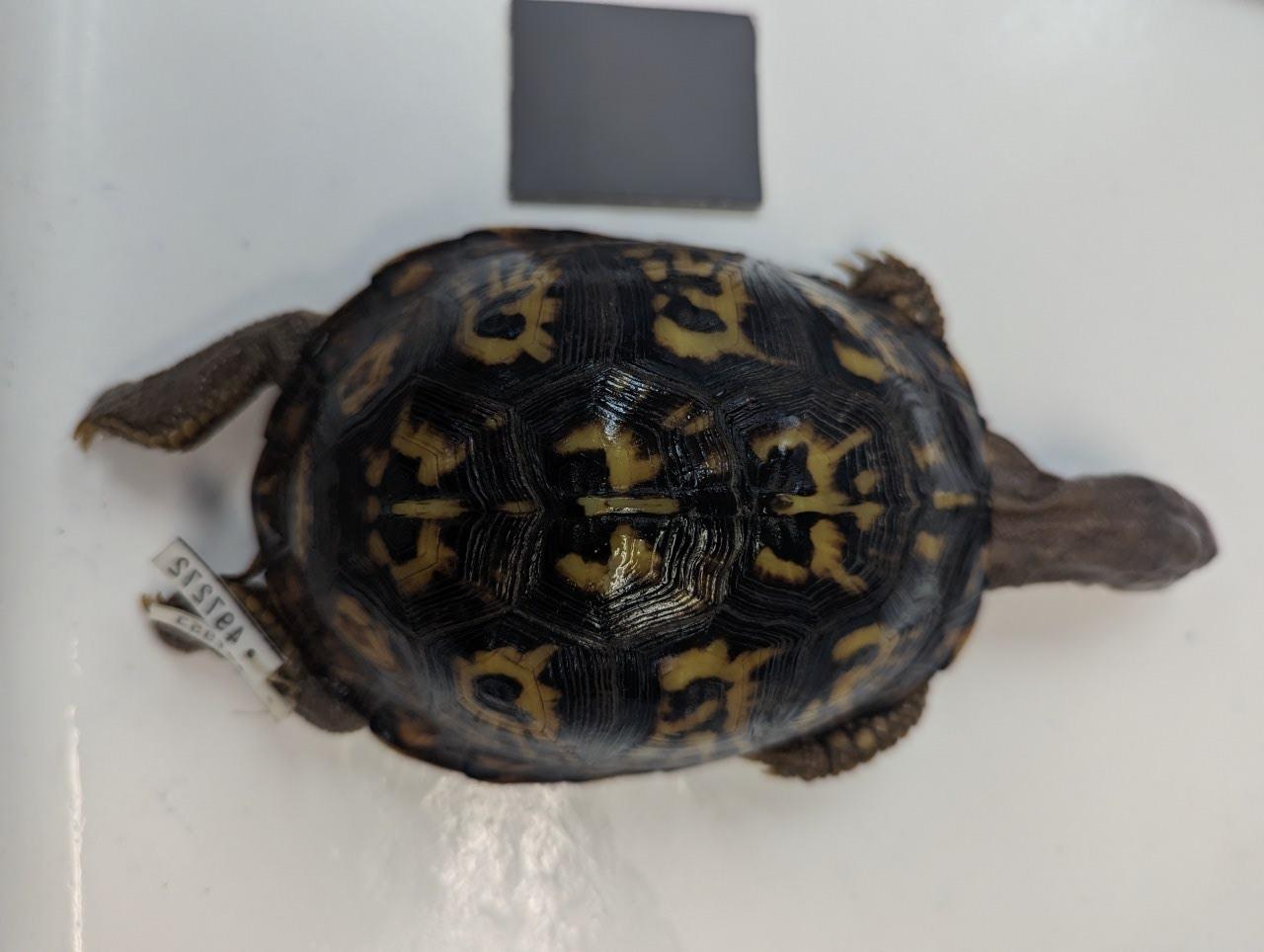

Supplement: Supplemental Information 6 — 98 photos of 98 turtles (single photo, all top view) that were used for the Citizen Science classification analysis. [file peerj-13-19690-s006.zip › TurtleClassification/TURTLE1062.jpg]

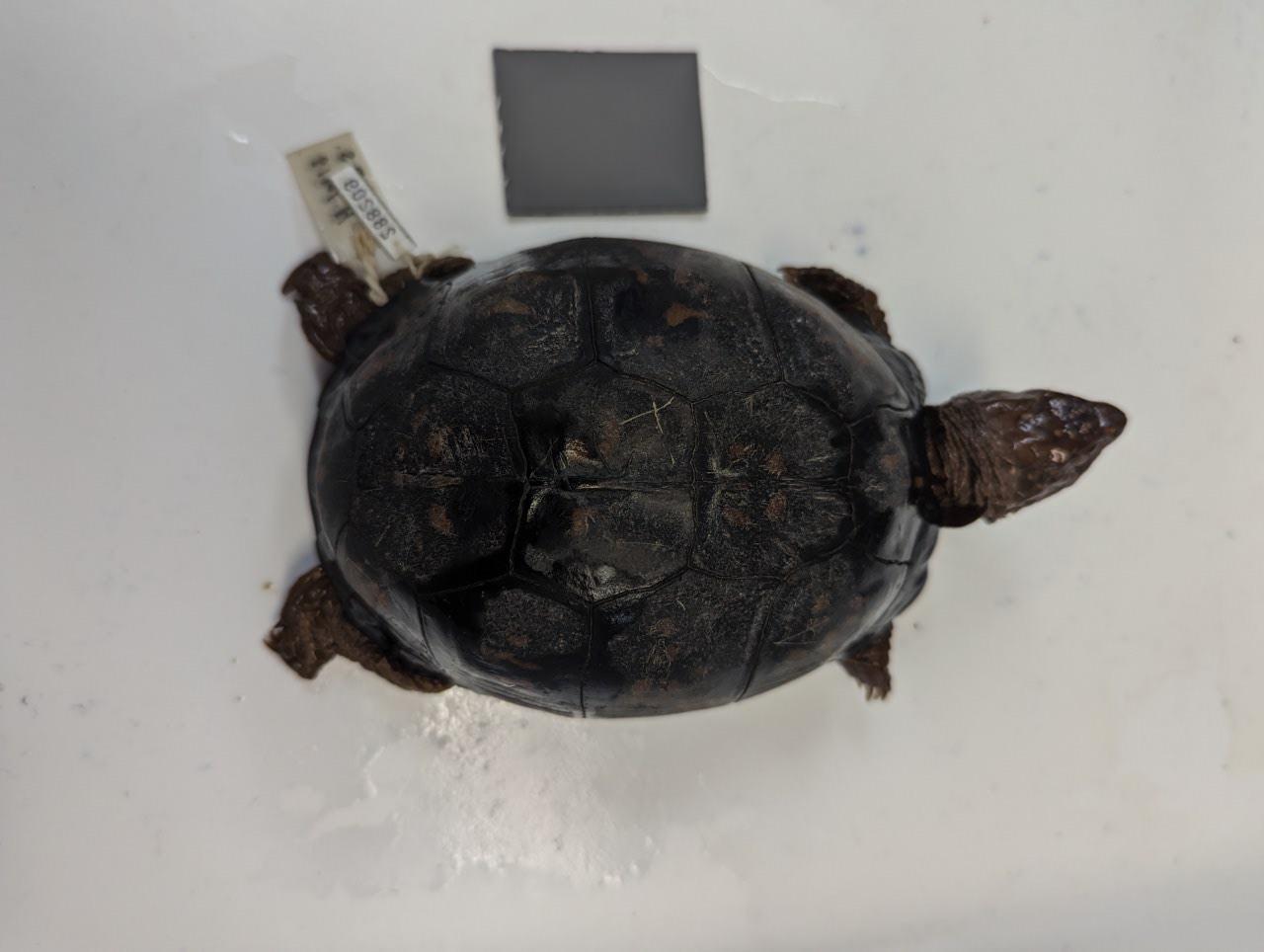

Supplement: Supplemental Information 6 — 98 photos of 98 turtles (single photo, all top view) that were used for the Citizen Science classification analysis. [file peerj-13-19690-s006.zip › TurtleClassification/TURTLE1063.jpg]

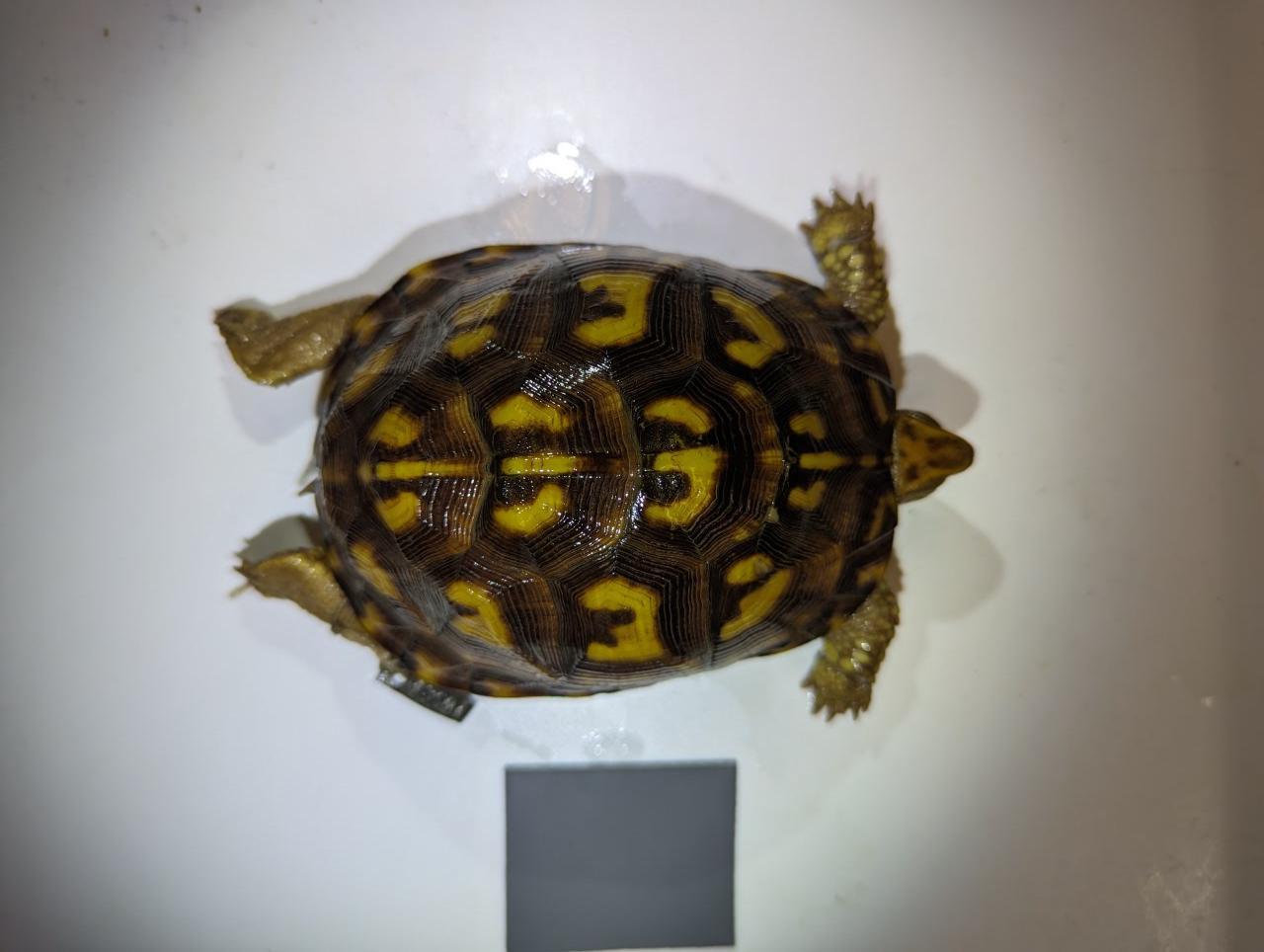

Supplement: Supplemental Information 6 — 98 photos of 98 turtles (single photo, all top view) that were used for the Citizen Science classification analysis. [file peerj-13-19690-s006.zip › TurtleClassification/TURTLE1064.jpg]

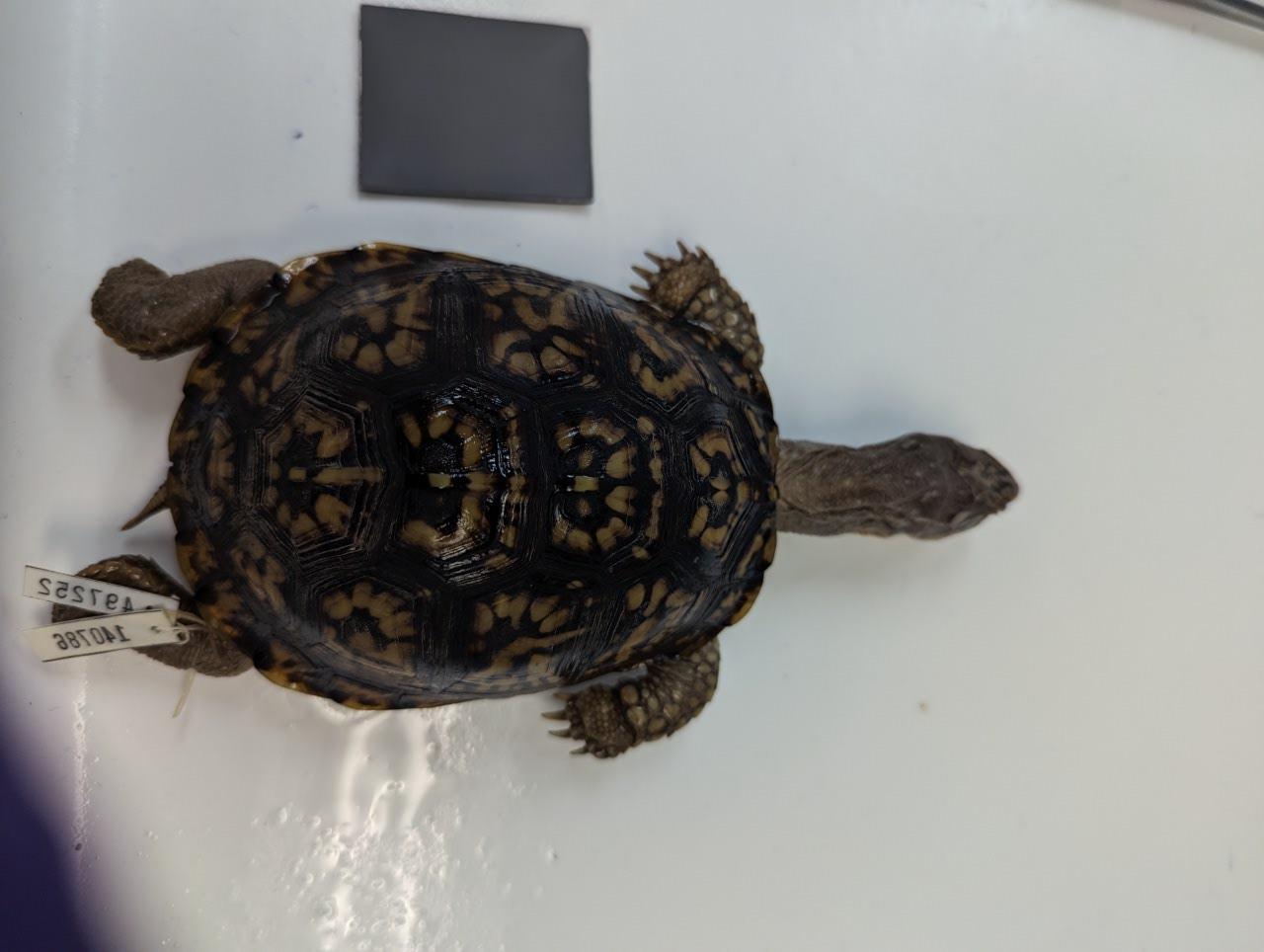

Supplement: Supplemental Information 6 — 98 photos of 98 turtles (single photo, all top view) that were used for the Citizen Science classification analysis. [file peerj-13-19690-s006.zip › TurtleClassification/TURTLE1065.jpg]

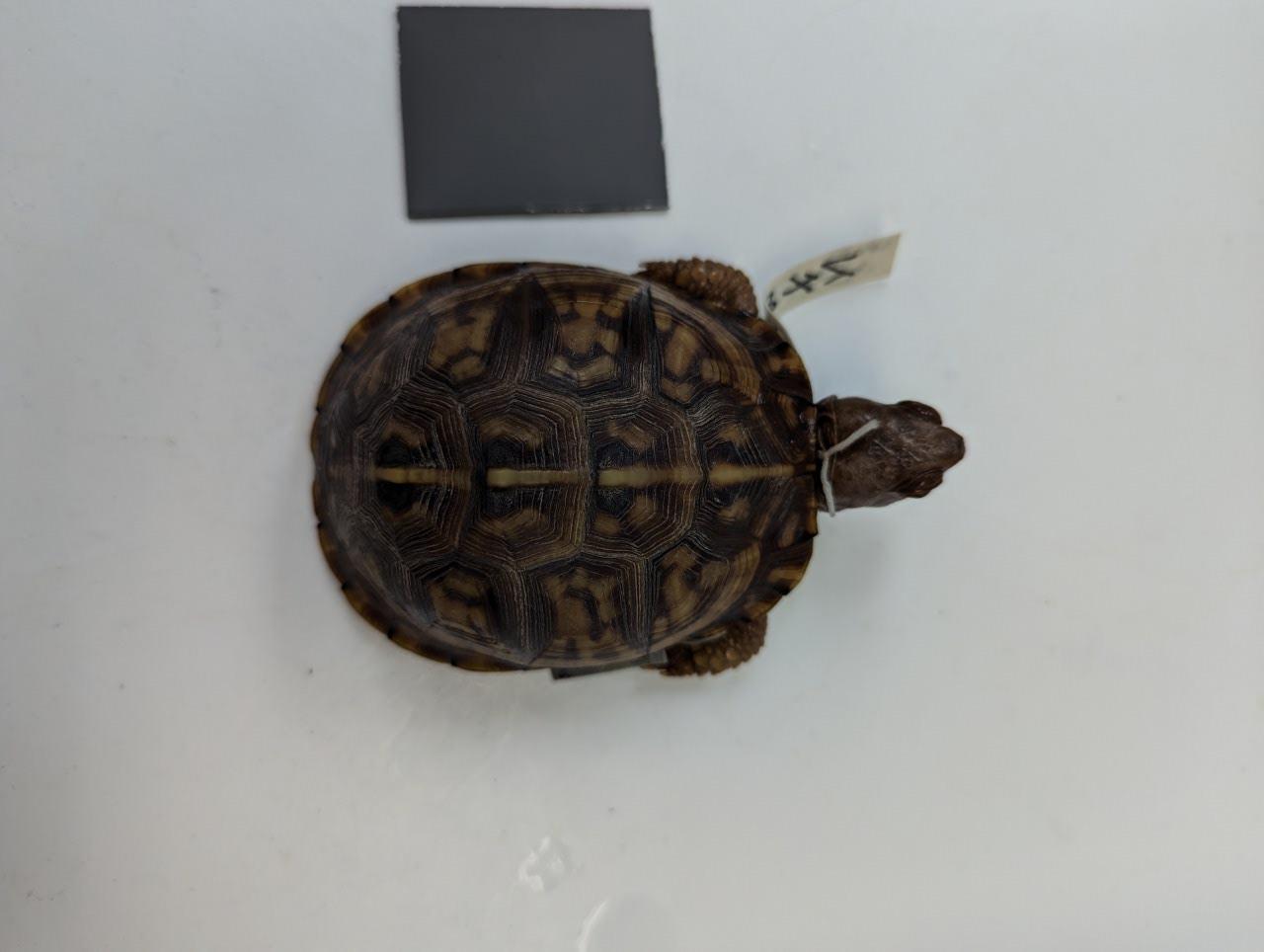

Supplement: Supplemental Information 6 — 98 photos of 98 turtles (single photo, all top view) that were used for the Citizen Science classification analysis. [file peerj-13-19690-s006.zip › TurtleClassification/TURTLE1066.jpg]

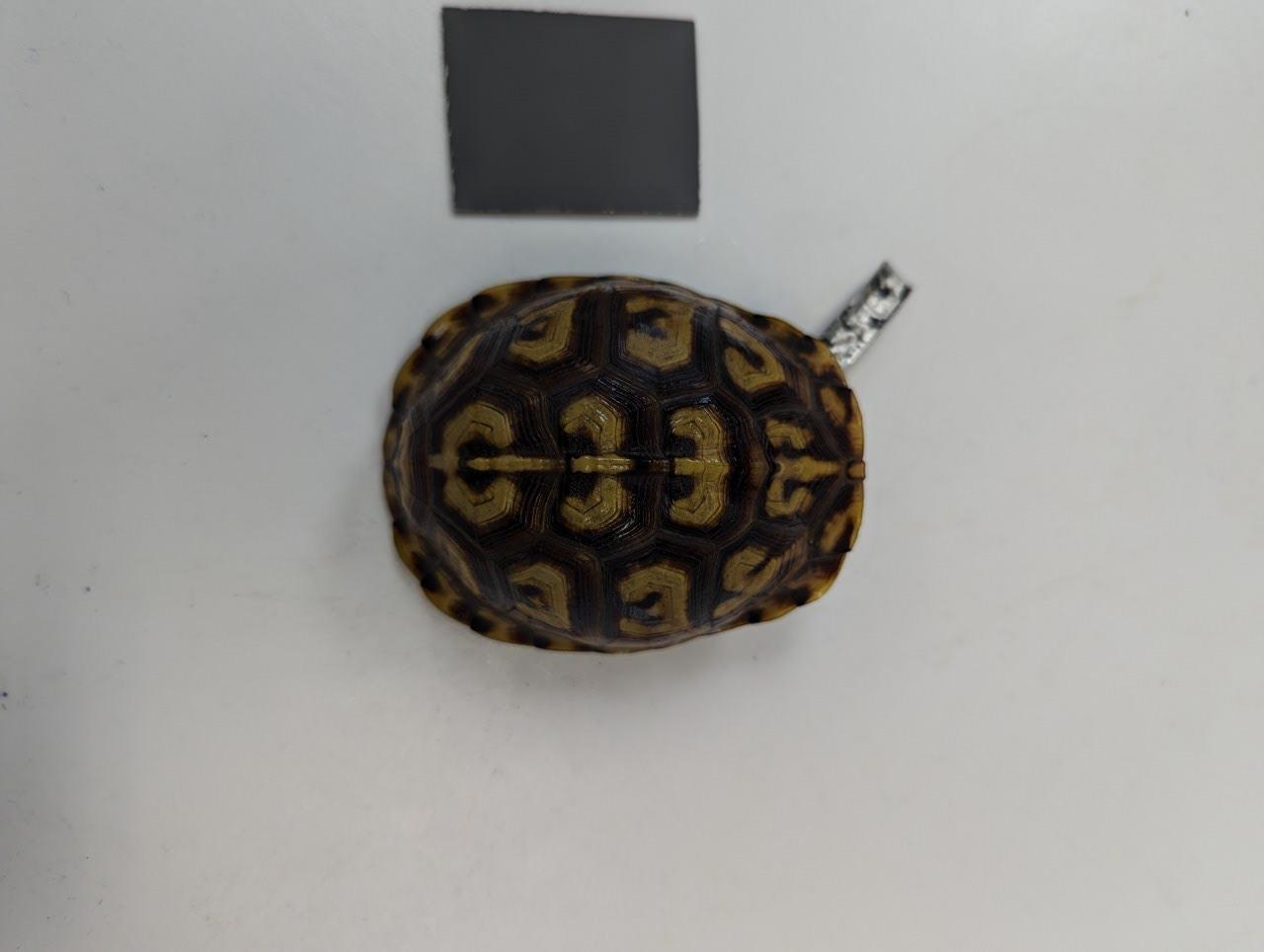

Supplement: Supplemental Information 6 — 98 photos of 98 turtles (single photo, all top view) that were used for the Citizen Science classification analysis. [file peerj-13-19690-s006.zip › TurtleClassification/TURTLE1067.jpg]

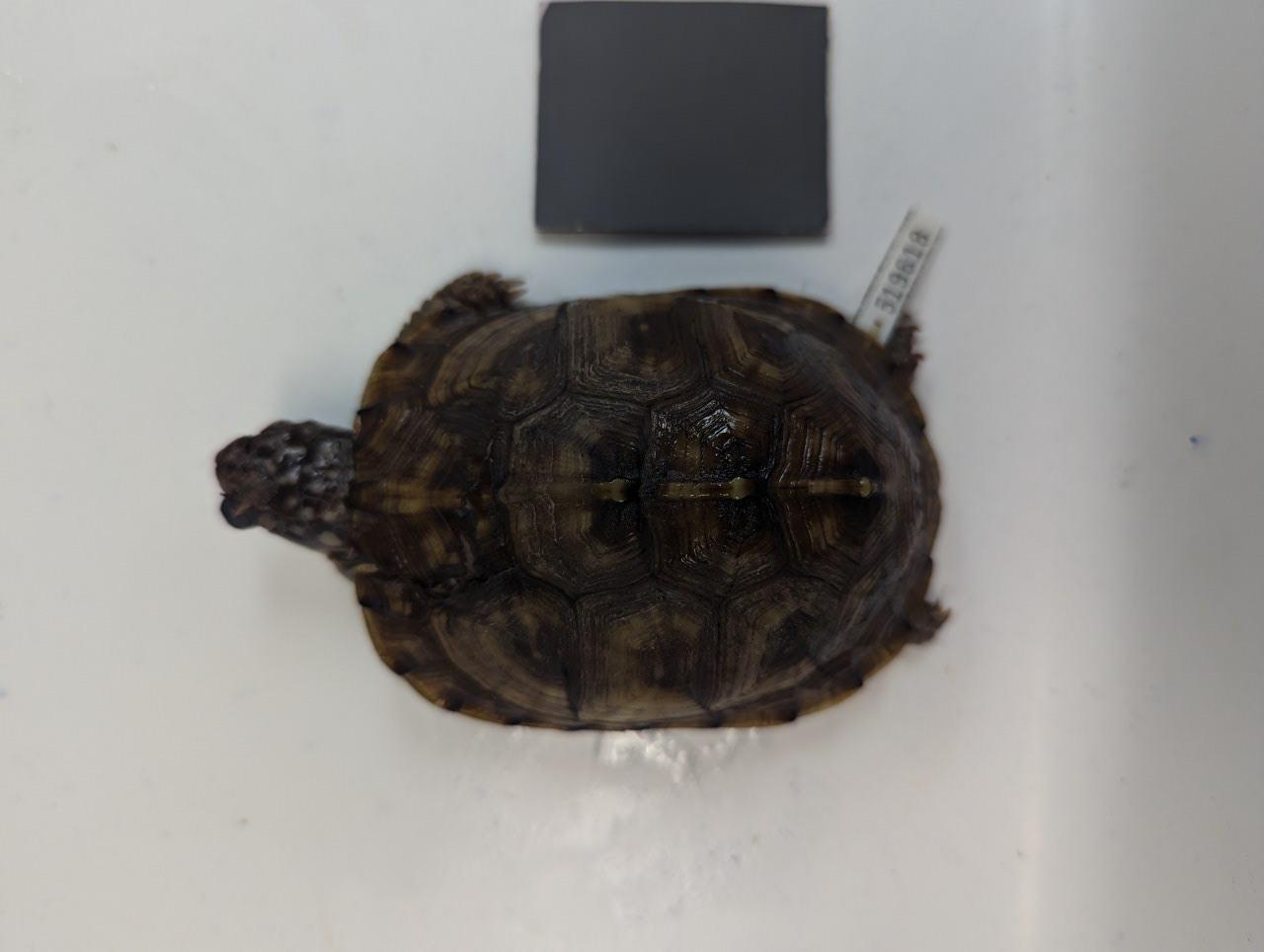

Supplement: Supplemental Information 6 — 98 photos of 98 turtles (single photo, all top view) that were used for the Citizen Science classification analysis. [file peerj-13-19690-s006.zip › TurtleClassification/TURTLE1068.jpg]

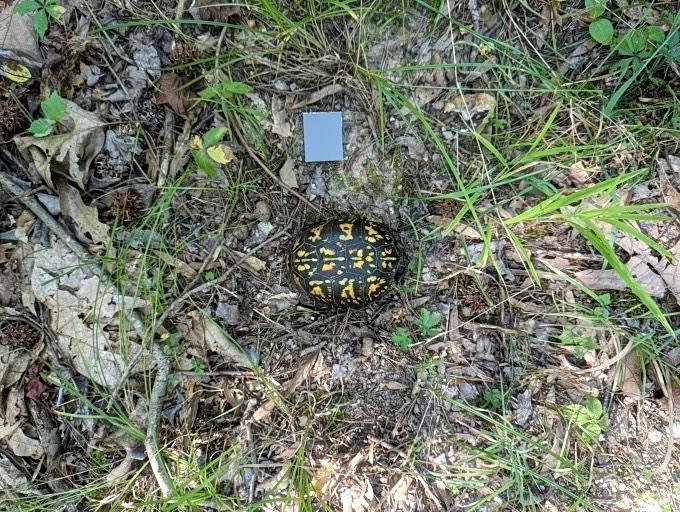

Supplement: Supplemental Information 6 — 98 photos of 98 turtles (single photo, all top view) that were used for the Citizen Science classification analysis. [file peerj-13-19690-s006.zip › TurtleClassification/TURTLE1069.jpg]

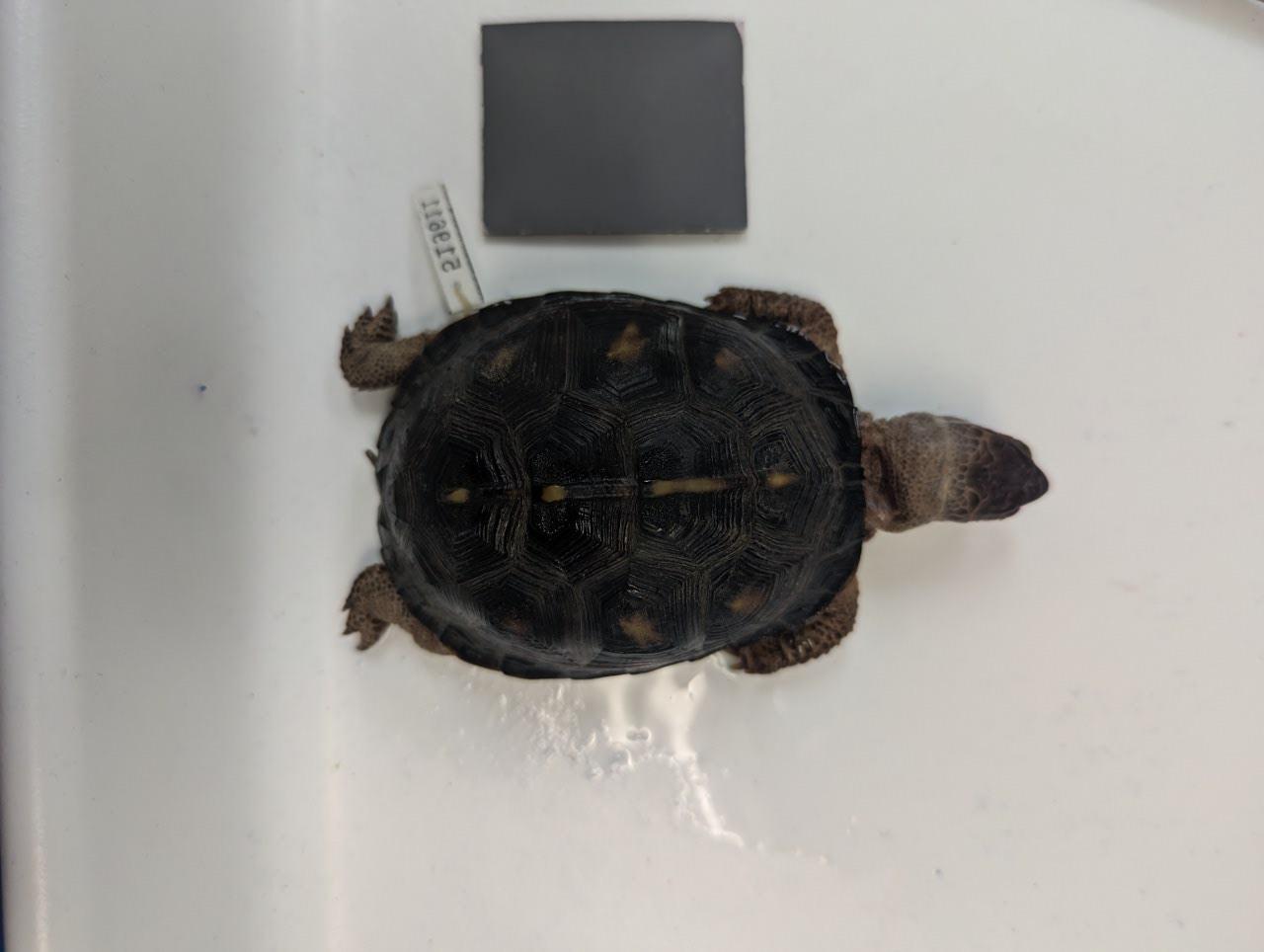

Supplement: Supplemental Information 6 — 98 photos of 98 turtles (single photo, all top view) that were used for the Citizen Science classification analysis. [file peerj-13-19690-s006.zip › TurtleClassification/TURTLE1070.jpg]

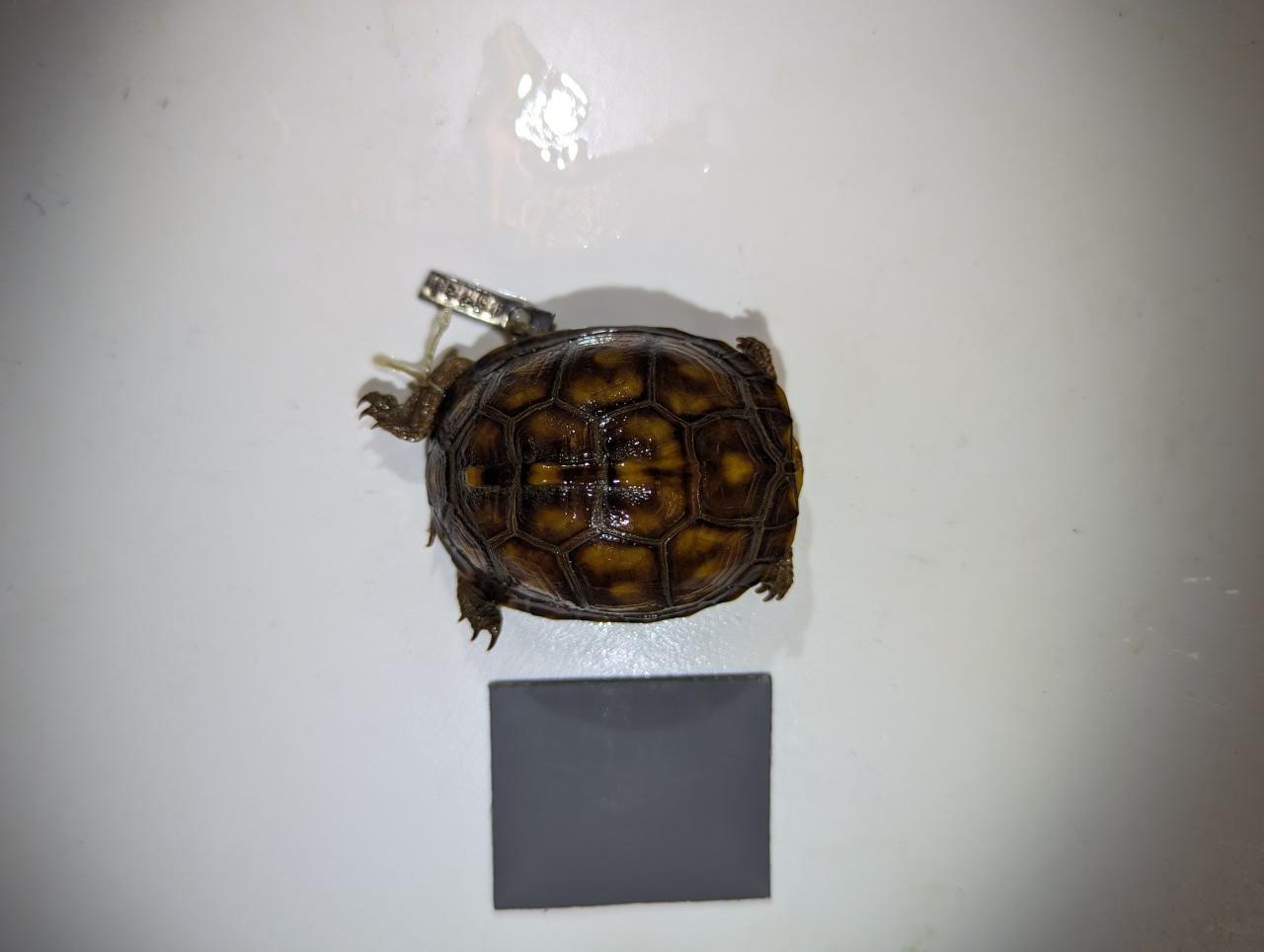

Supplement: Supplemental Information 6 — 98 photos of 98 turtles (single photo, all top view) that were used for the Citizen Science classification analysis. [file peerj-13-19690-s006.zip › TurtleClassification/TURTLE1071.jpg]

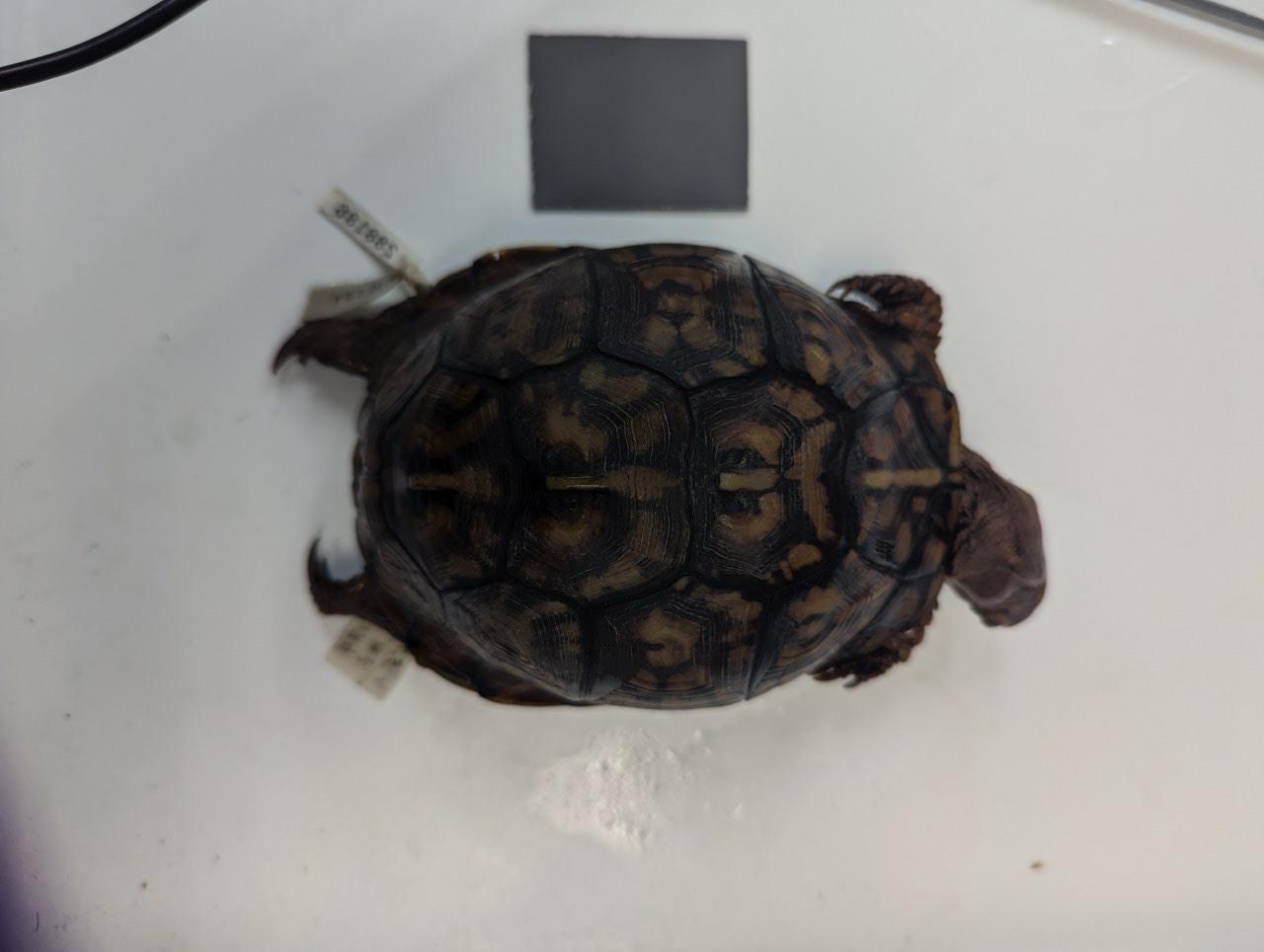

Supplement: Supplemental Information 6 — 98 photos of 98 turtles (single photo, all top view) that were used for the Citizen Science classification analysis. [file peerj-13-19690-s006.zip › TurtleClassification/TURTLE1072.jpg]

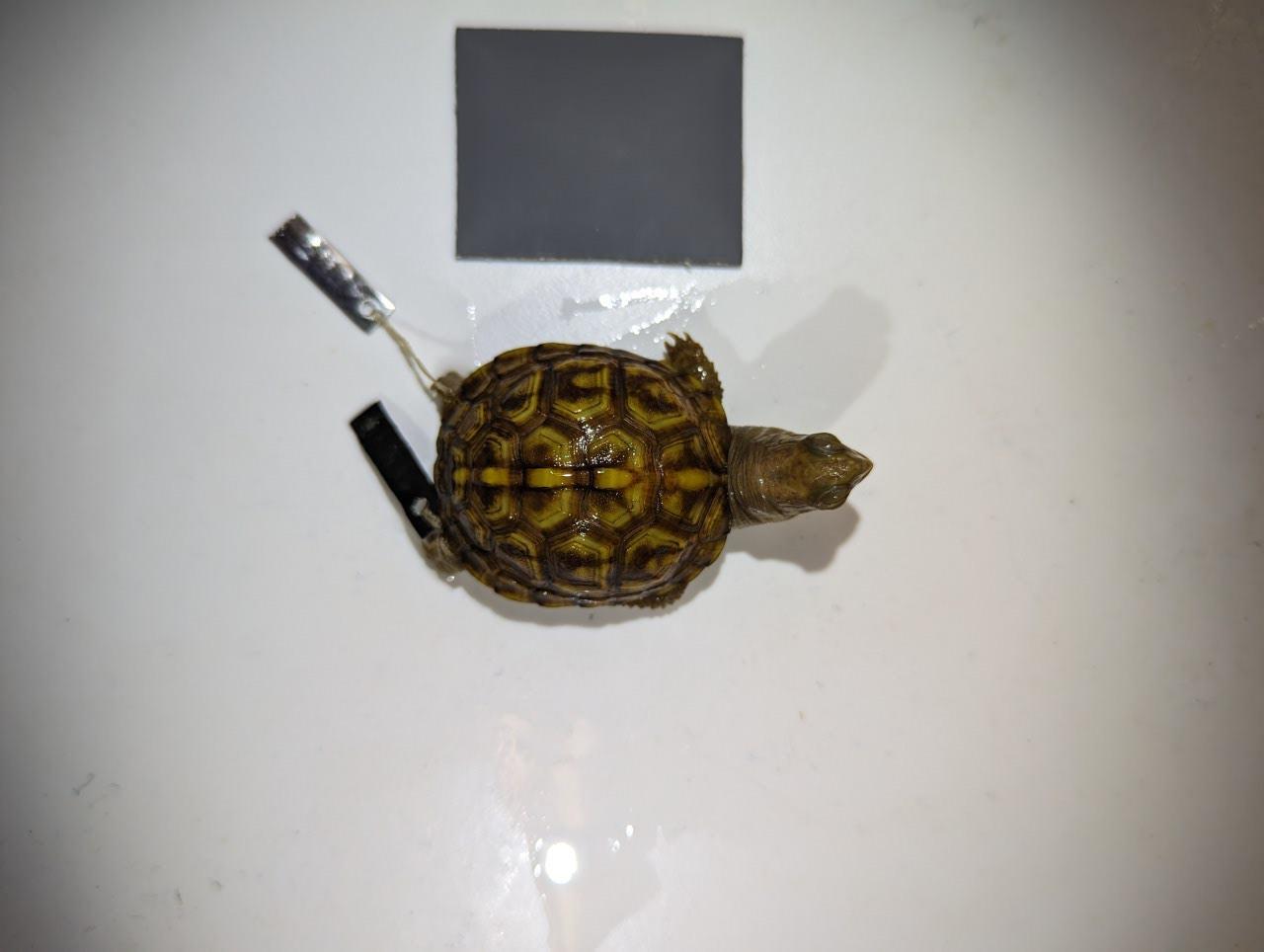

Supplement: Supplemental Information 6 — 98 photos of 98 turtles (single photo, all top view) that were used for the Citizen Science classification analysis. [file peerj-13-19690-s006.zip › TurtleClassification/TURTLE1073.jpg]

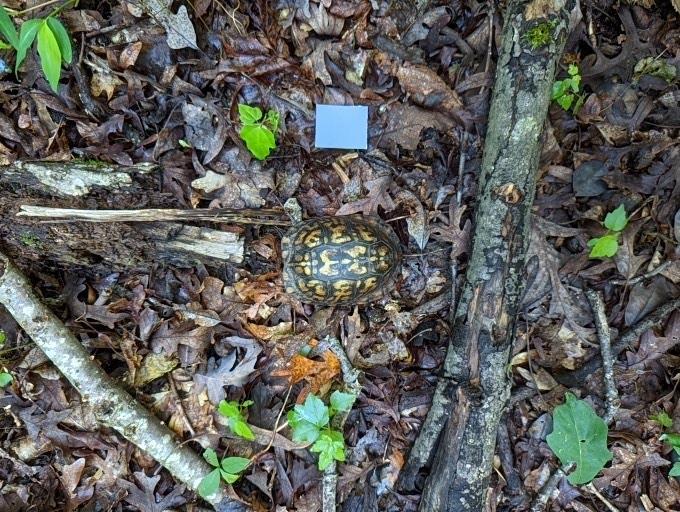

Supplement: Supplemental Information 6 — 98 photos of 98 turtles (single photo, all top view) that were used for the Citizen Science classification analysis. [file peerj-13-19690-s006.zip › TurtleClassification/TURTLE1074.jpg]

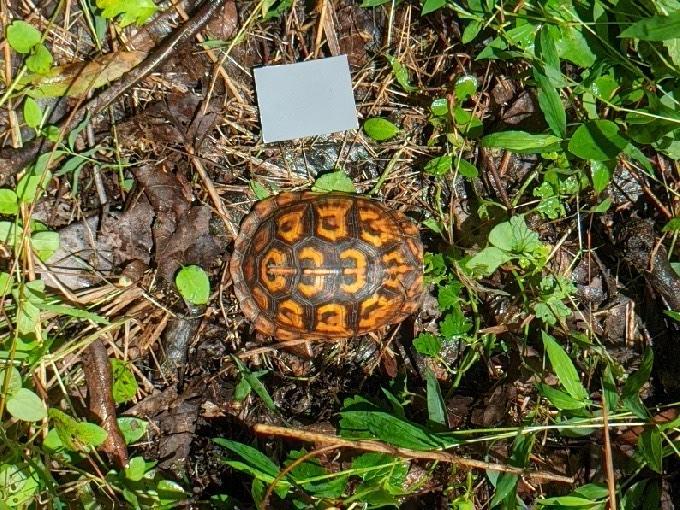

Supplement: Supplemental Information 6 — 98 photos of 98 turtles (single photo, all top view) that were used for the Citizen Science classification analysis. [file peerj-13-19690-s006.zip › TurtleClassification/TURTLE1075.jpg]

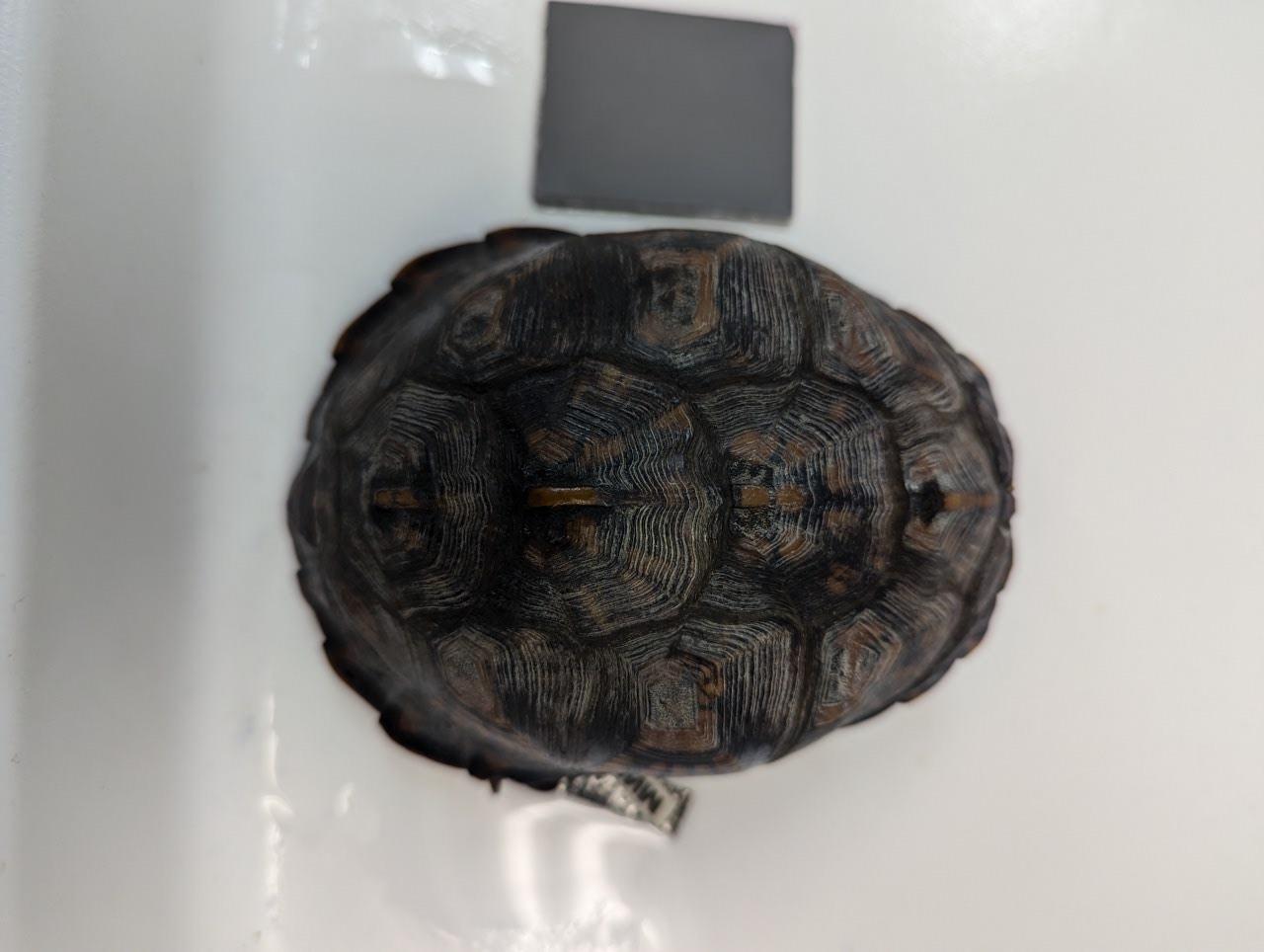

Supplement: Supplemental Information 6 — 98 photos of 98 turtles (single photo, all top view) that were used for the Citizen Science classification analysis. [file peerj-13-19690-s006.zip › TurtleClassification/TURTLE1076.jpg]

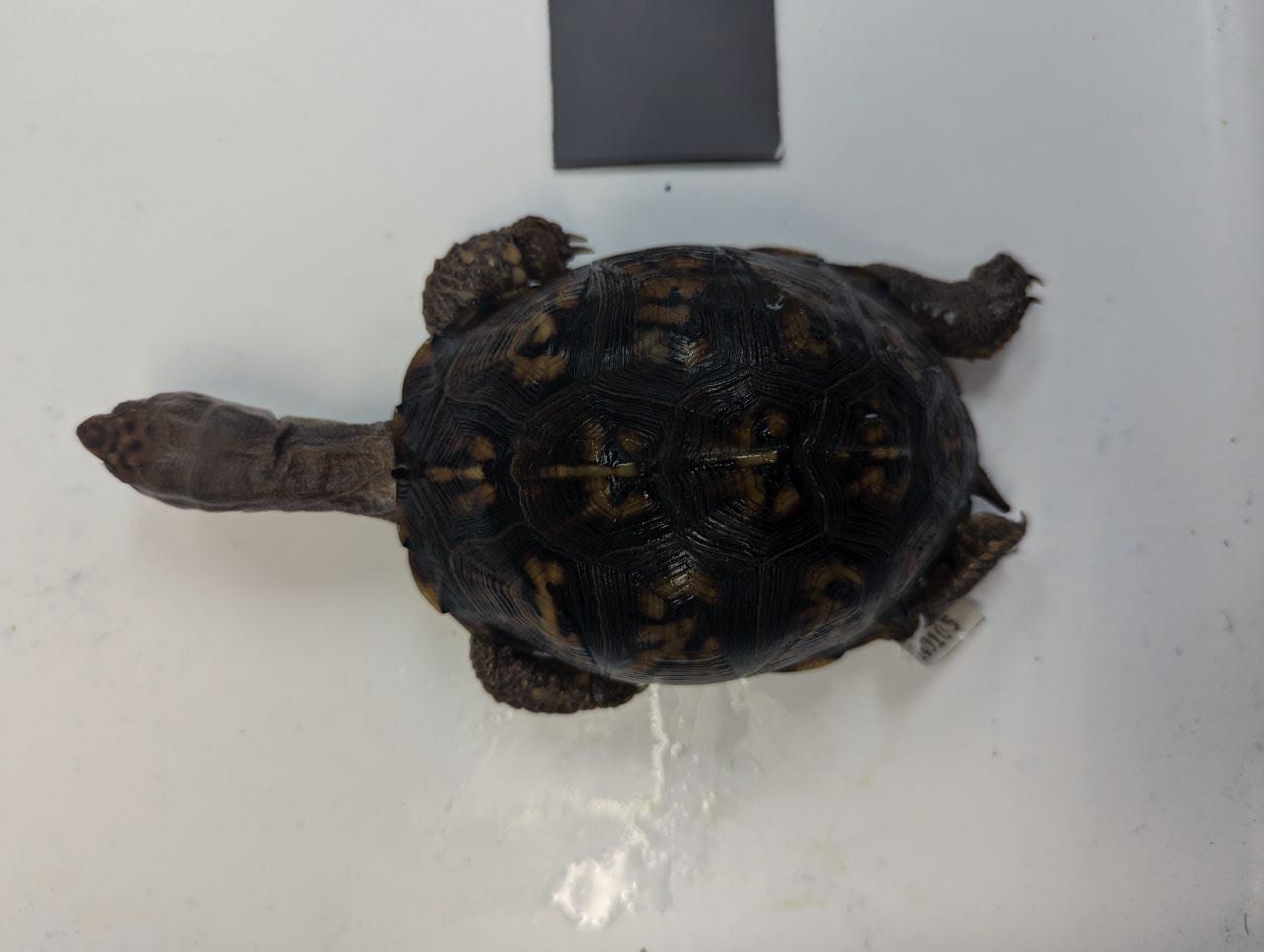

Supplement: Supplemental Information 6 — 98 photos of 98 turtles (single photo, all top view) that were used for the Citizen Science classification analysis. [file peerj-13-19690-s006.zip › TurtleClassification/TURTLE1077.jpg]

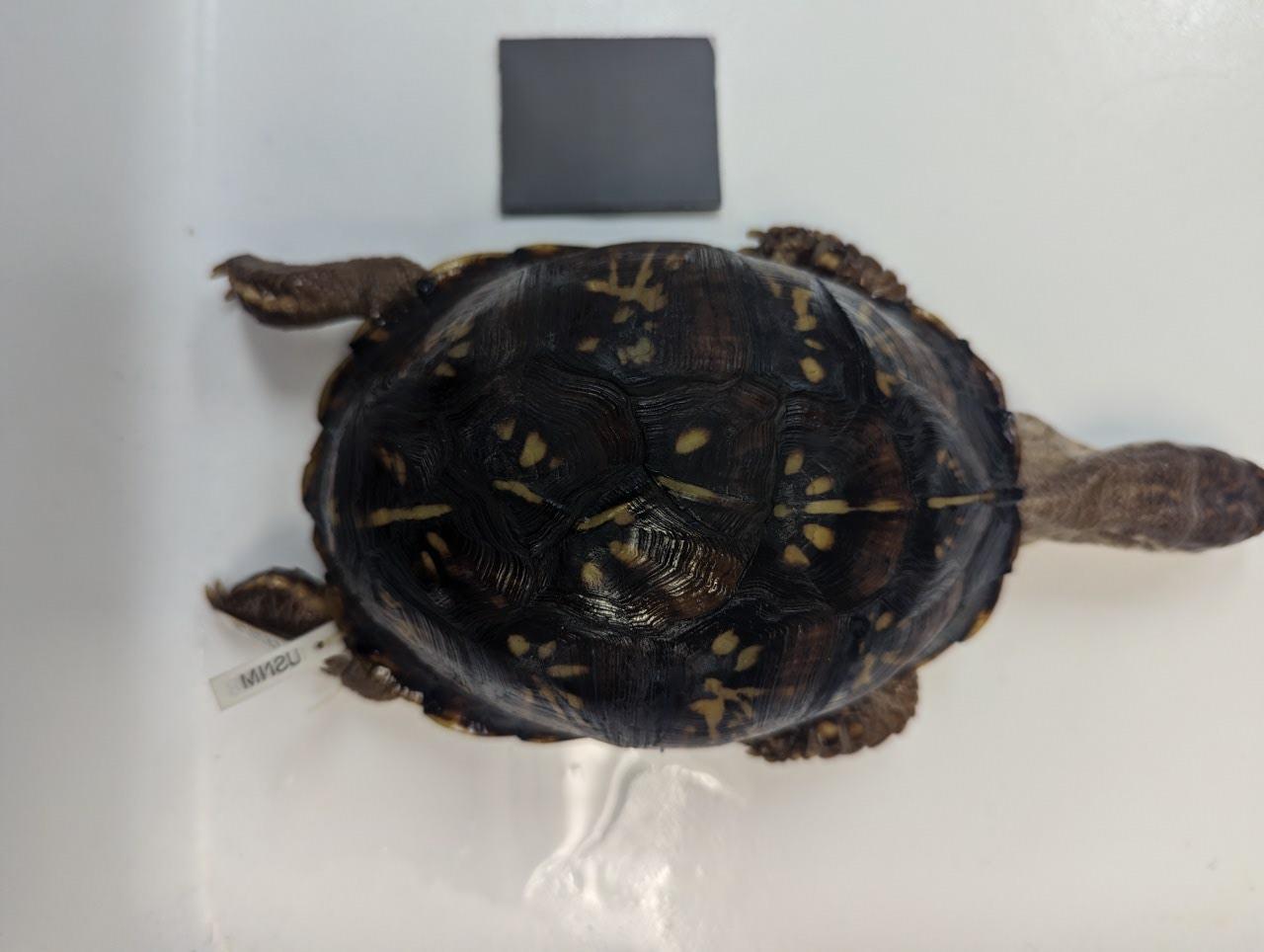

Supplement: Supplemental Information 6 — 98 photos of 98 turtles (single photo, all top view) that were used for the Citizen Science classification analysis. [file peerj-13-19690-s006.zip › TurtleClassification/TURTLE1078.jpg]

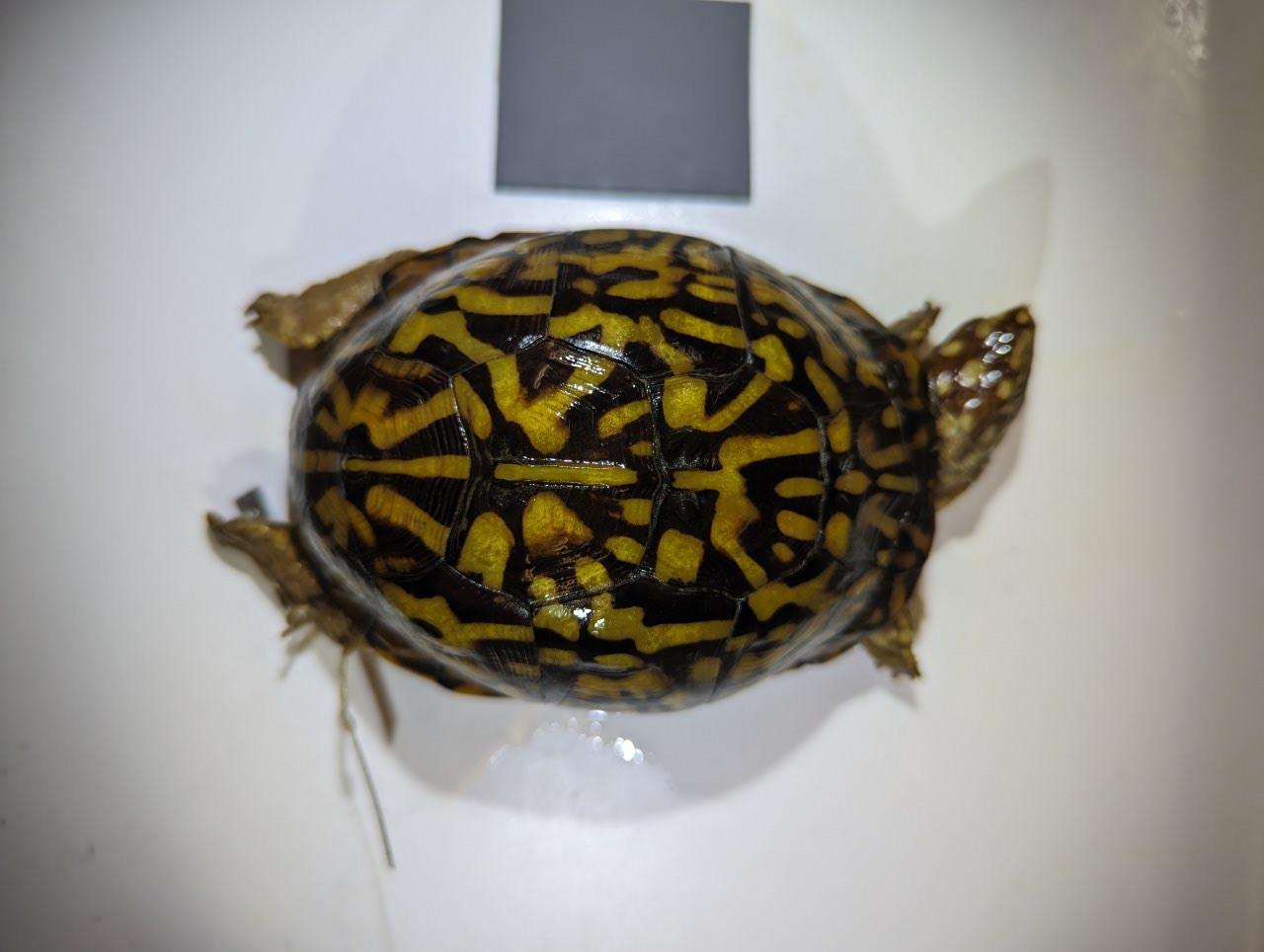

Supplement: Supplemental Information 6 — 98 photos of 98 turtles (single photo, all top view) that were used for the Citizen Science classification analysis. [file peerj-13-19690-s006.zip › TurtleClassification/TURTLE1079.jpg]

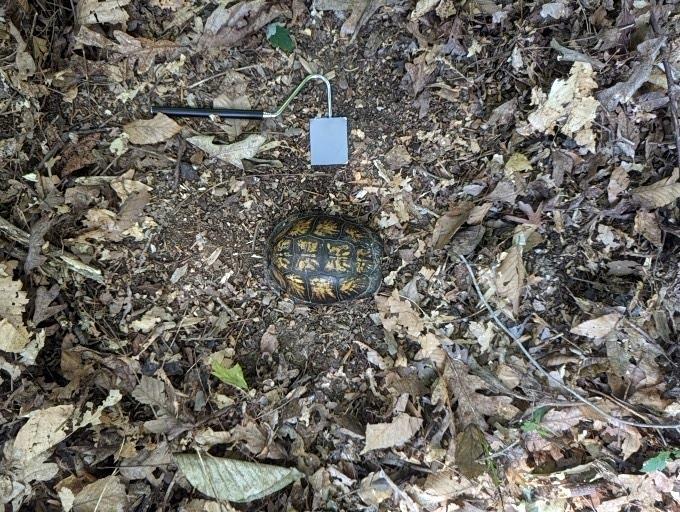

Supplement: Supplemental Information 6 — 98 photos of 98 turtles (single photo, all top view) that were used for the Citizen Science classification analysis. [file peerj-13-19690-s006.zip › TurtleClassification/TURTLE1080.jpg]

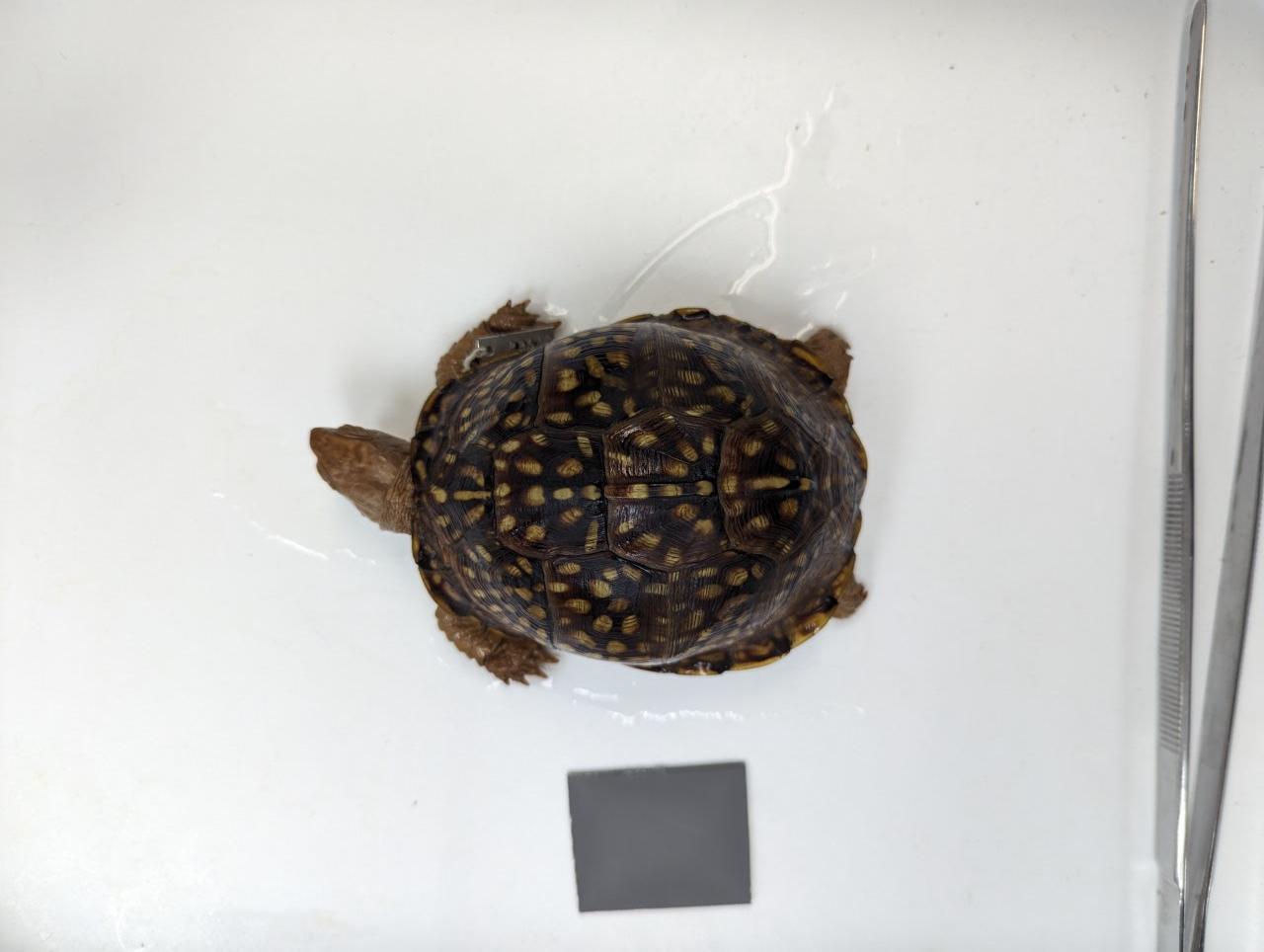

Supplement: Supplemental Information 6 — 98 photos of 98 turtles (single photo, all top view) that were used for the Citizen Science classification analysis. [file peerj-13-19690-s006.zip › TurtleClassification/TURTLE1081.jpg]

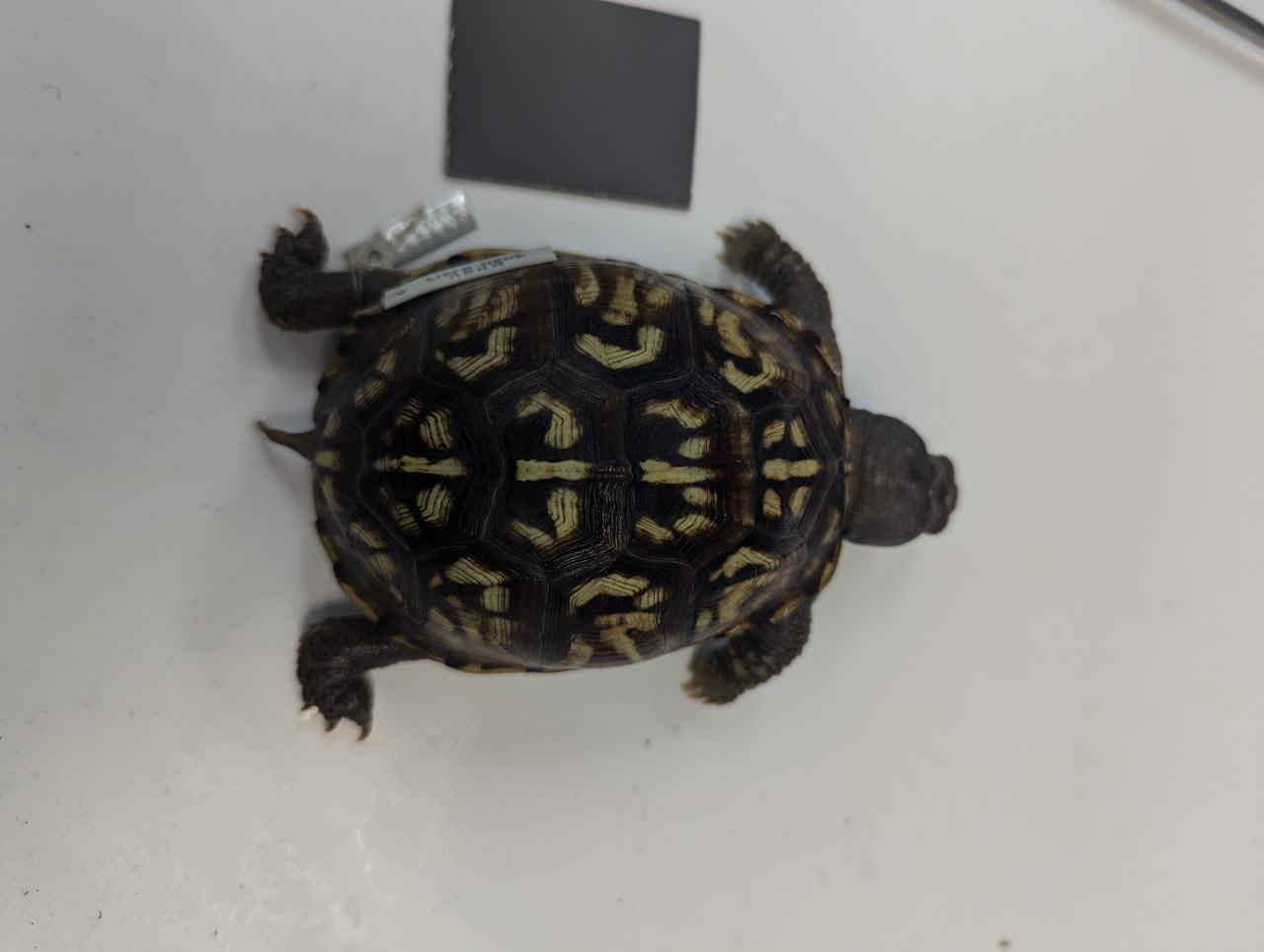

Supplement: Supplemental Information 6 — 98 photos of 98 turtles (single photo, all top view) that were used for the Citizen Science classification analysis. [file peerj-13-19690-s006.zip › TurtleClassification/TURTLE1082.jpg]

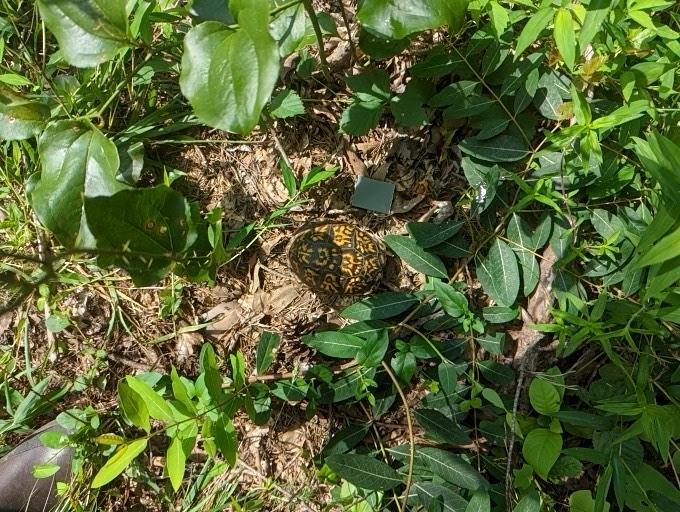

Supplement: Supplemental Information 6 — 98 photos of 98 turtles (single photo, all top view) that were used for the Citizen Science classification analysis. [file peerj-13-19690-s006.zip › TurtleClassification/TURTLE1083.jpg]

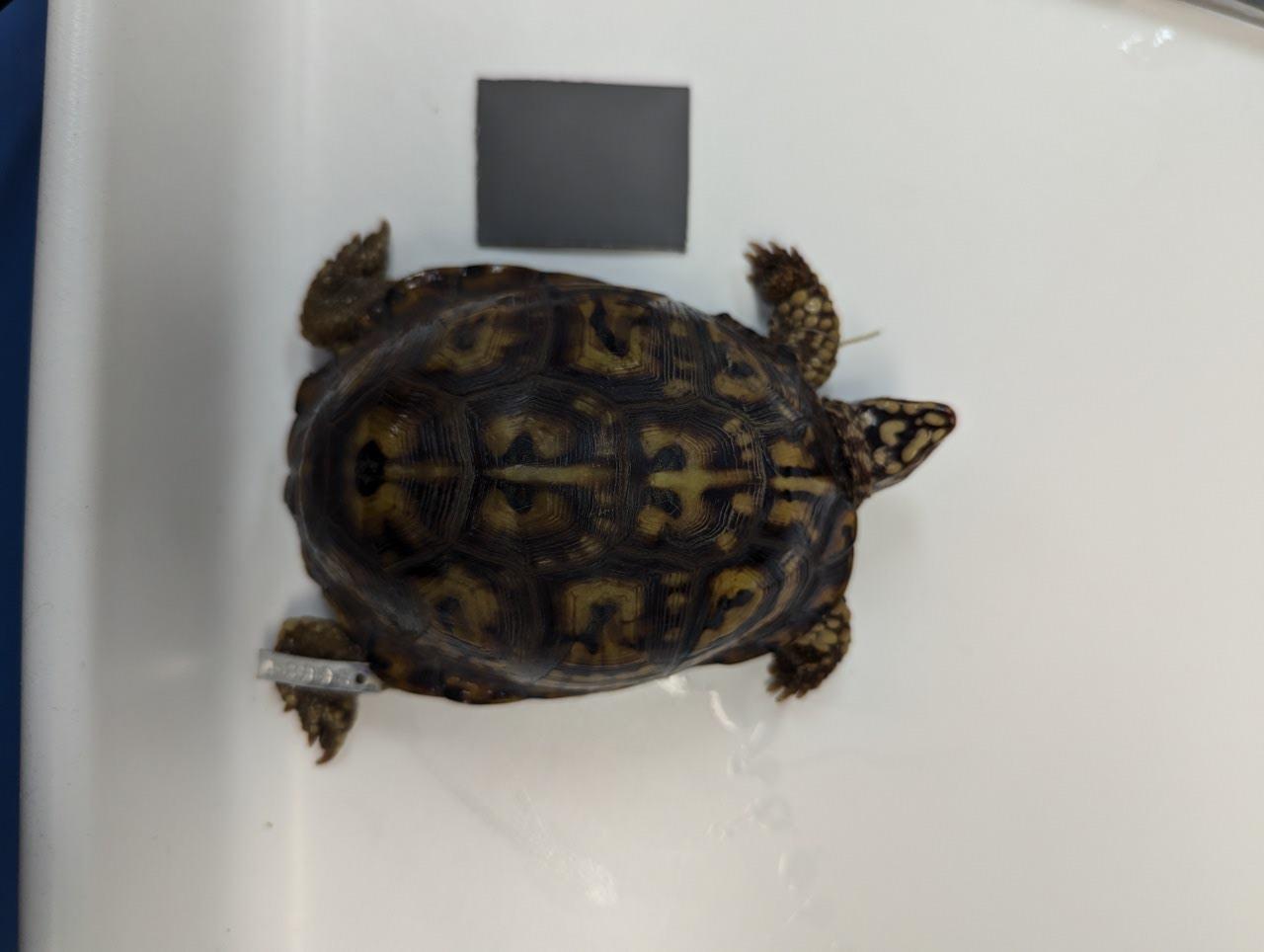

Supplement: Supplemental Information 6 — 98 photos of 98 turtles (single photo, all top view) that were used for the Citizen Science classification analysis. [file peerj-13-19690-s006.zip › TurtleClassification/TURTLE1084.jpg]

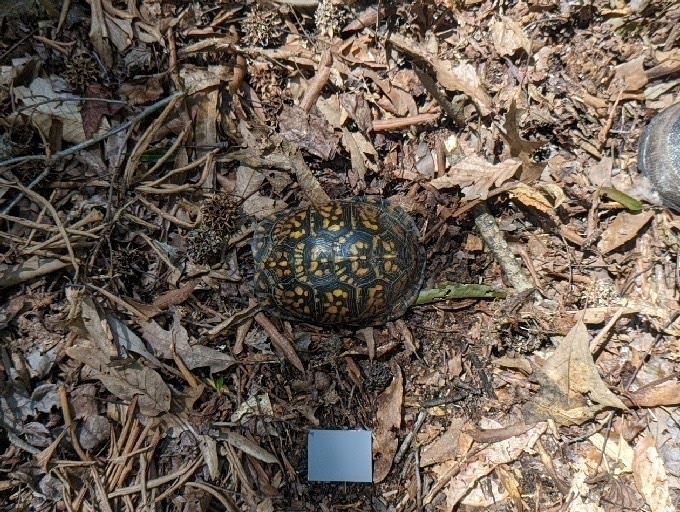

Supplement: Supplemental Information 6 — 98 photos of 98 turtles (single photo, all top view) that were used for the Citizen Science classification analysis. [file peerj-13-19690-s006.zip › TurtleClassification/TURTLE1085.jpg]

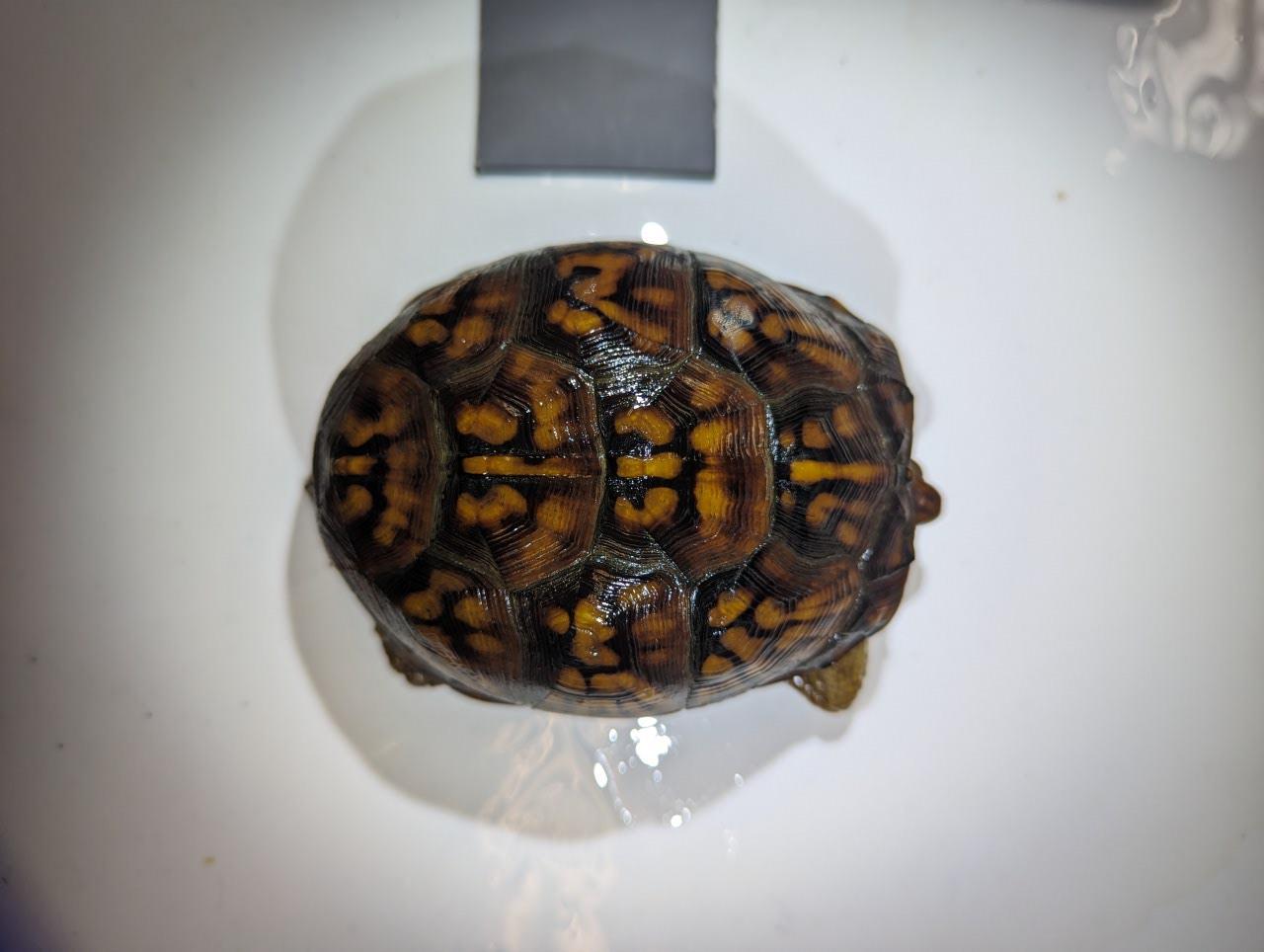

Supplement: Supplemental Information 6 — 98 photos of 98 turtles (single photo, all top view) that were used for the Citizen Science classification analysis. [file peerj-13-19690-s006.zip › TurtleClassification/TURTLE1086.jpg]

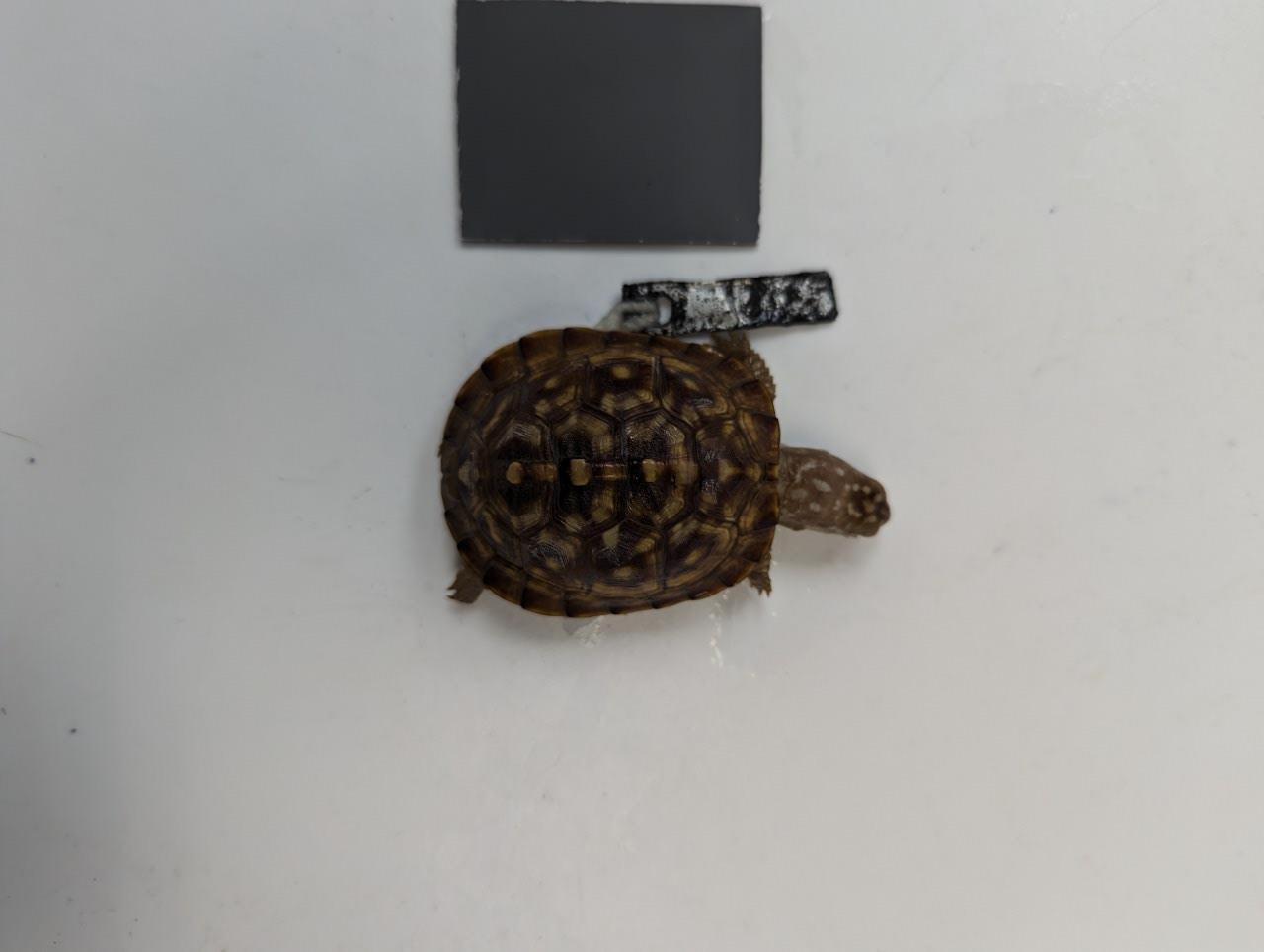

Supplement: Supplemental Information 6 — 98 photos of 98 turtles (single photo, all top view) that were used for the Citizen Science classification analysis. [file peerj-13-19690-s006.zip › TurtleClassification/TURTLE1087.jpg]

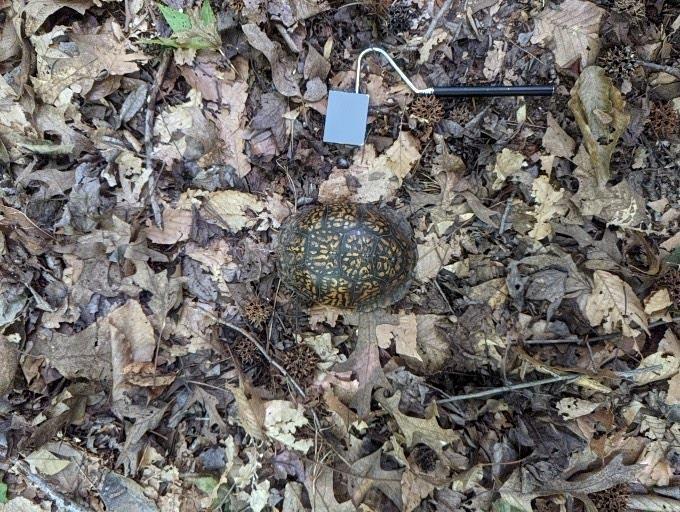

Supplement: Supplemental Information 6 — 98 photos of 98 turtles (single photo, all top view) that were used for the Citizen Science classification analysis. [file peerj-13-19690-s006.zip › TurtleClassification/TURTLE1088.jpg]

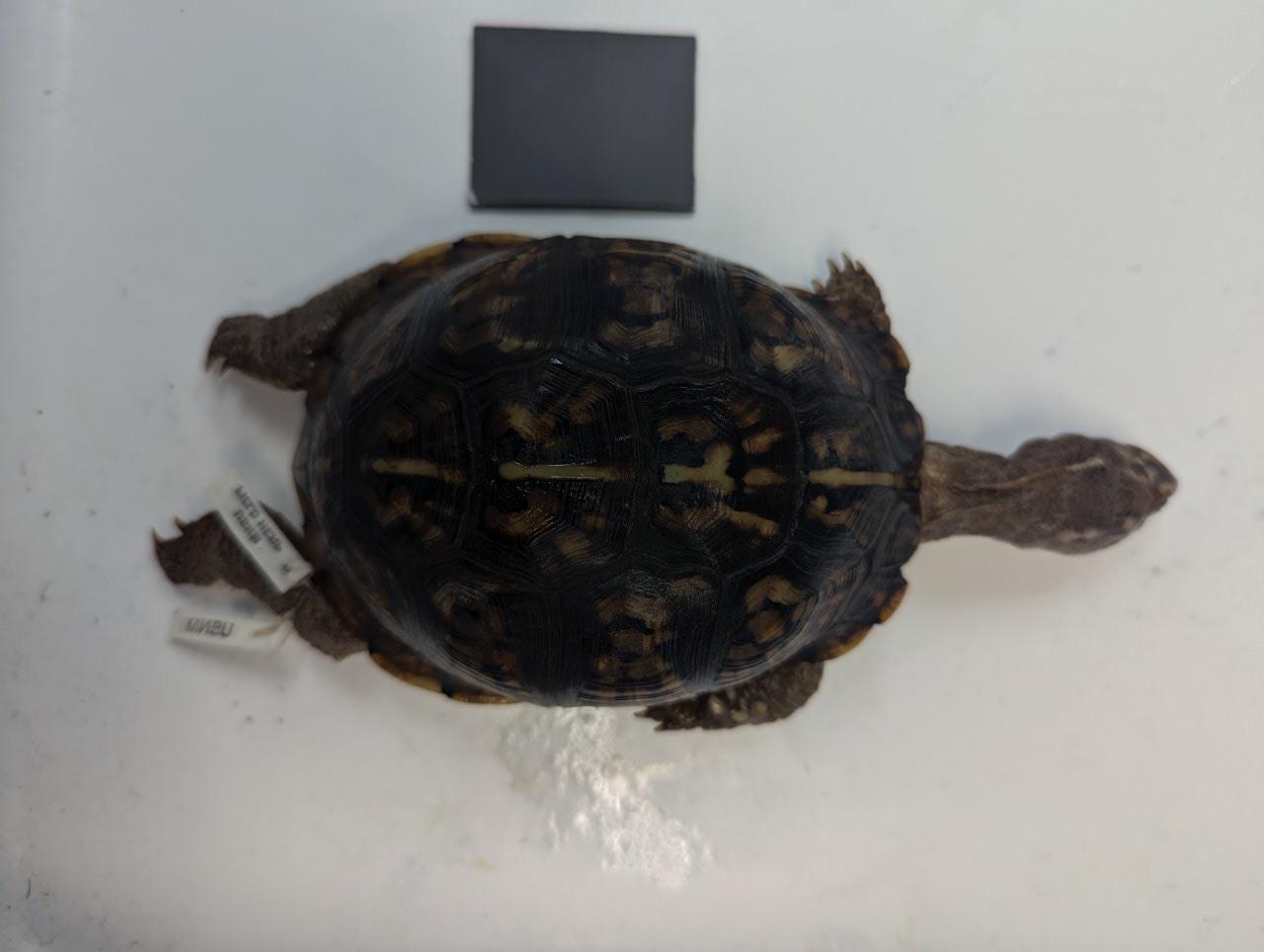

Supplement: Supplemental Information 6 — 98 photos of 98 turtles (single photo, all top view) that were used for the Citizen Science classification analysis. [file peerj-13-19690-s006.zip › TurtleClassification/TURTLE1089.jpg]

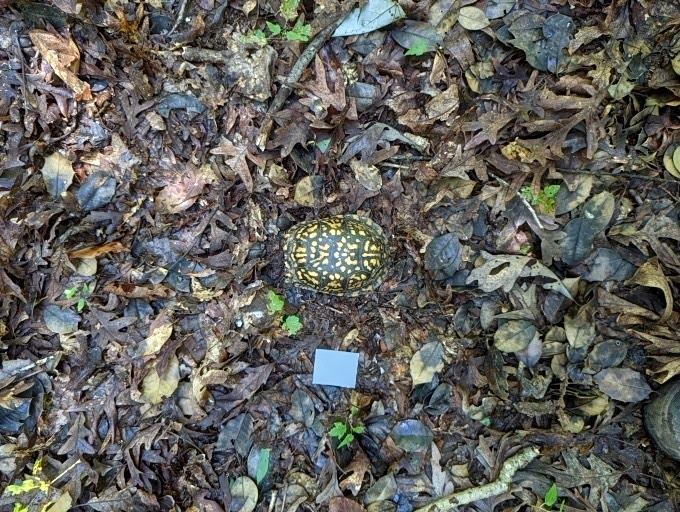

Supplement: Supplemental Information 6 — 98 photos of 98 turtles (single photo, all top view) that were used for the Citizen Science classification analysis. [file peerj-13-19690-s006.zip › TurtleClassification/TURTLE1090.jpg]

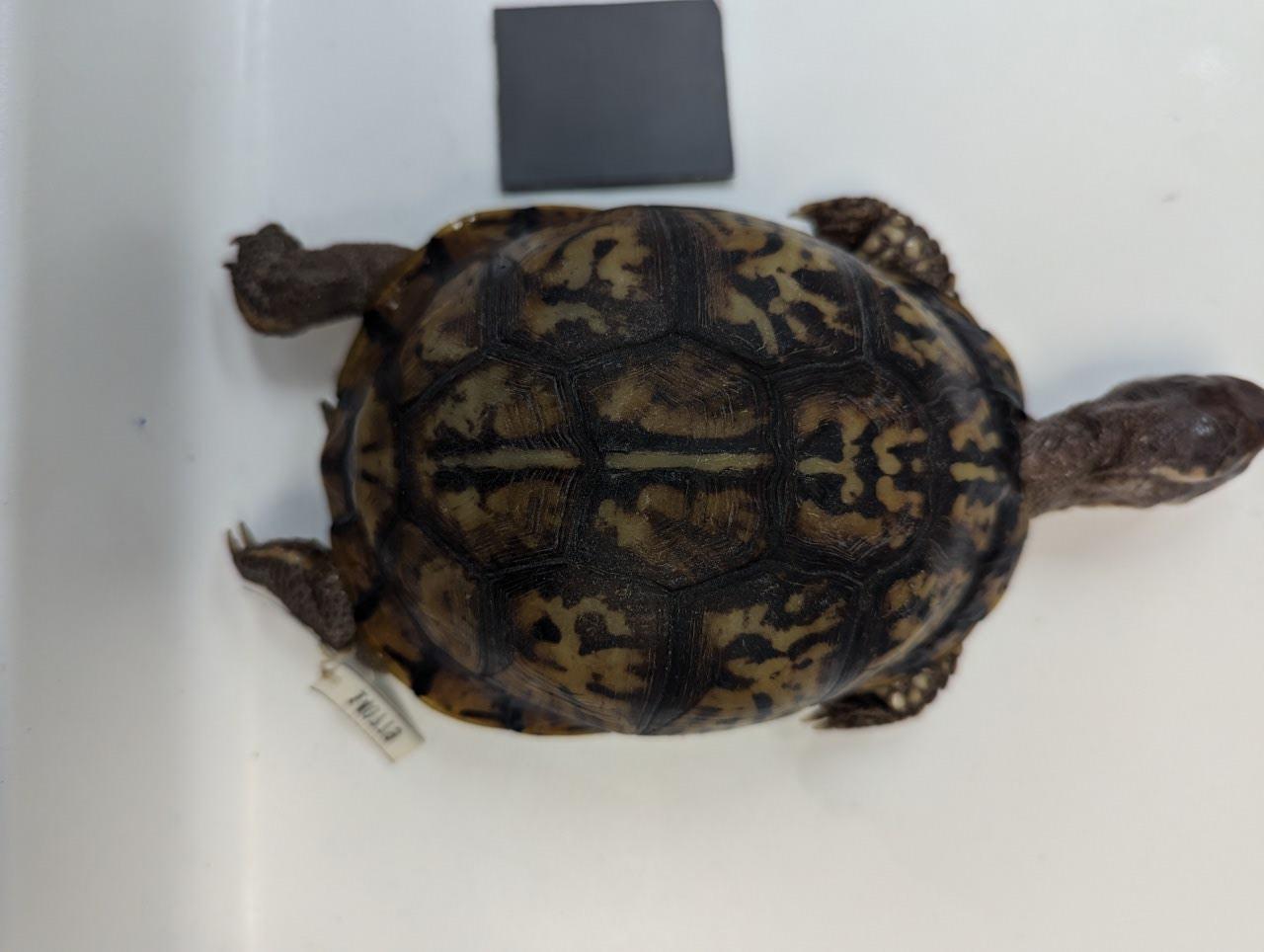

Supplement: Supplemental Information 6 — 98 photos of 98 turtles (single photo, all top view) that were used for the Citizen Science classification analysis. [file peerj-13-19690-s006.zip › TurtleClassification/TURTLE1091.jpg]

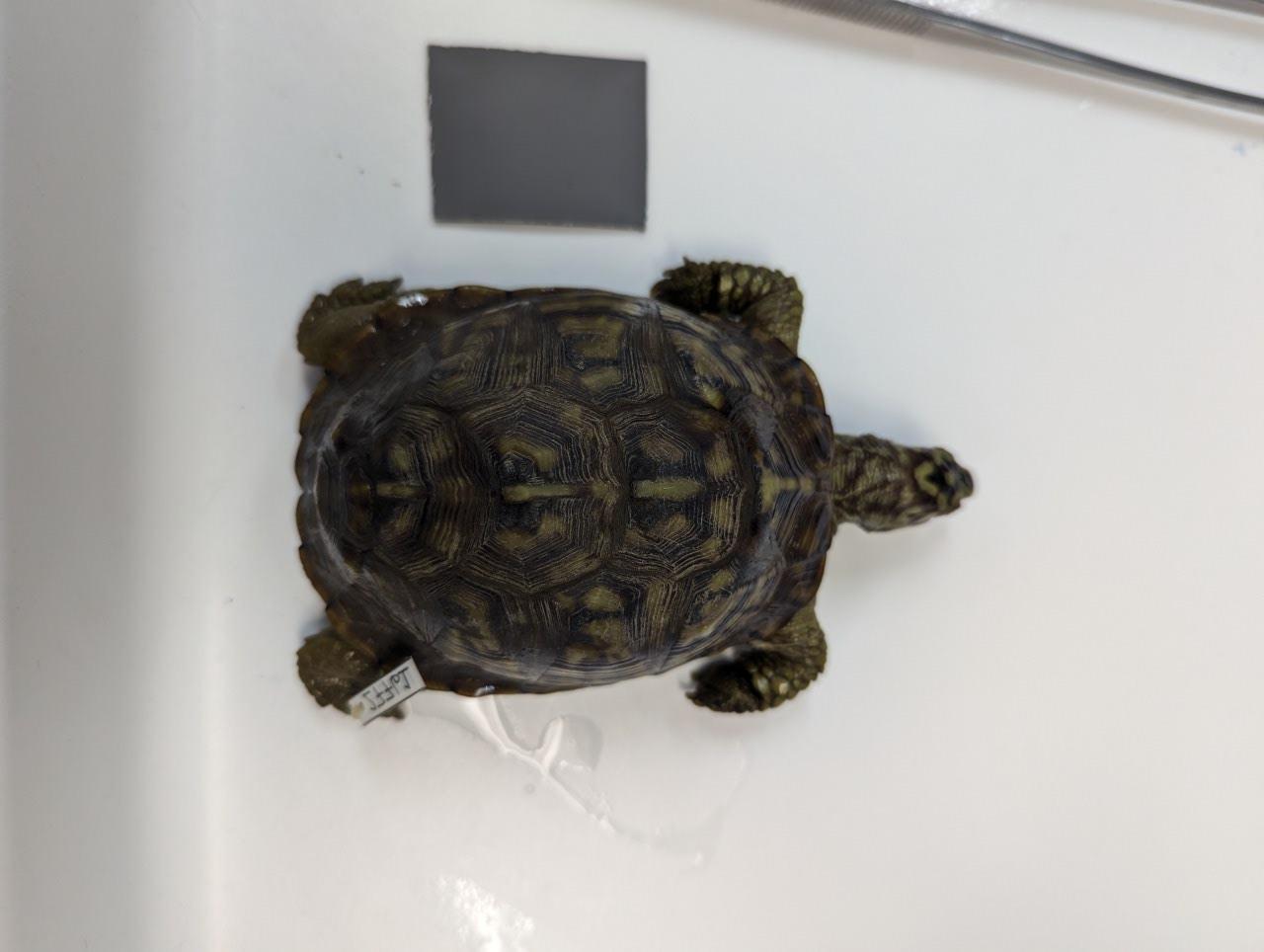

Supplement: Supplemental Information 6 — 98 photos of 98 turtles (single photo, all top view) that were used for the Citizen Science classification analysis. [file peerj-13-19690-s006.zip › TurtleClassification/TURTLE1092.jpg]

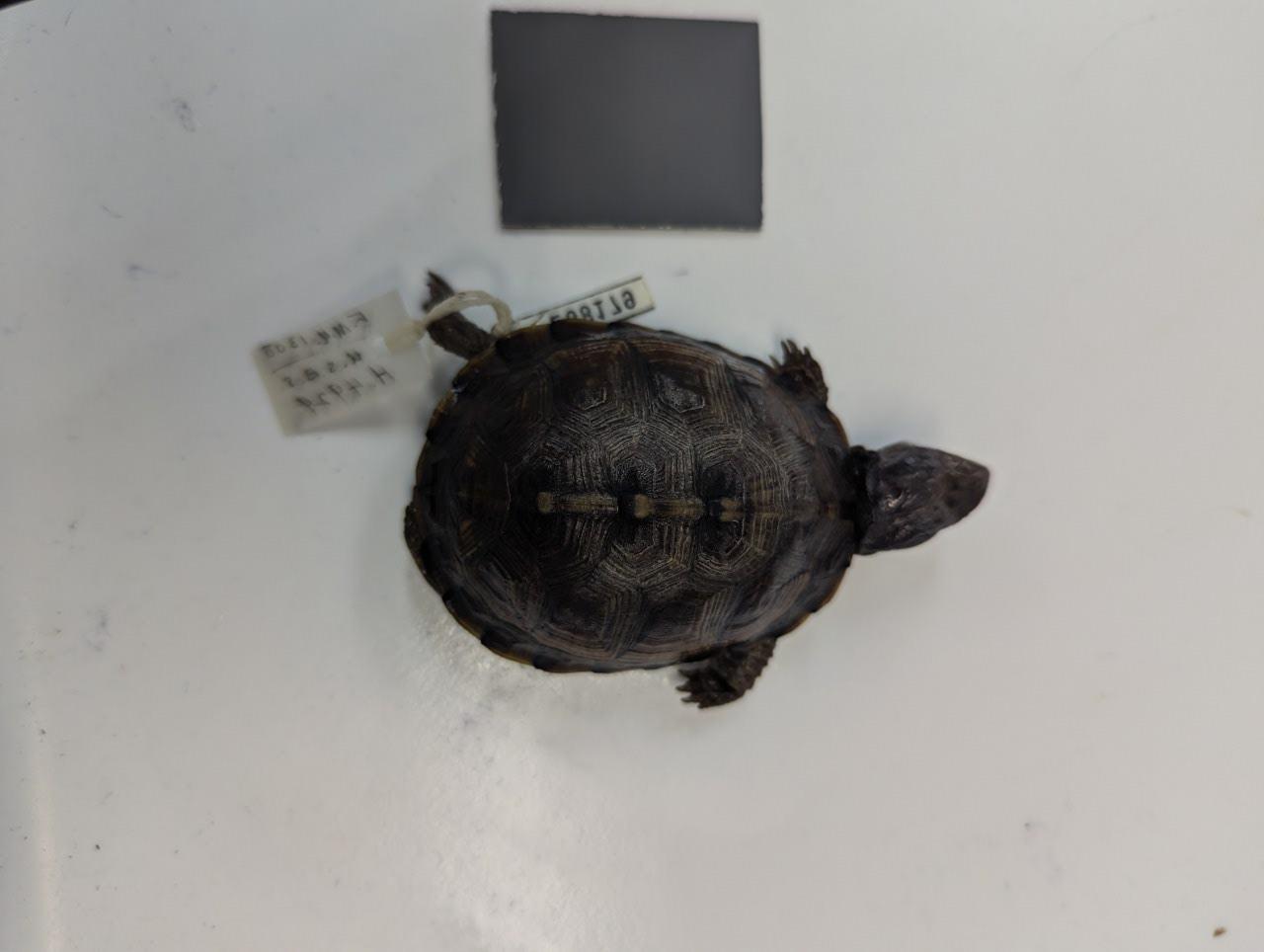

Supplement: Supplemental Information 6 — 98 photos of 98 turtles (single photo, all top view) that were used for the Citizen Science classification analysis. [file peerj-13-19690-s006.zip › TurtleClassification/TURTLE1093.jpg]

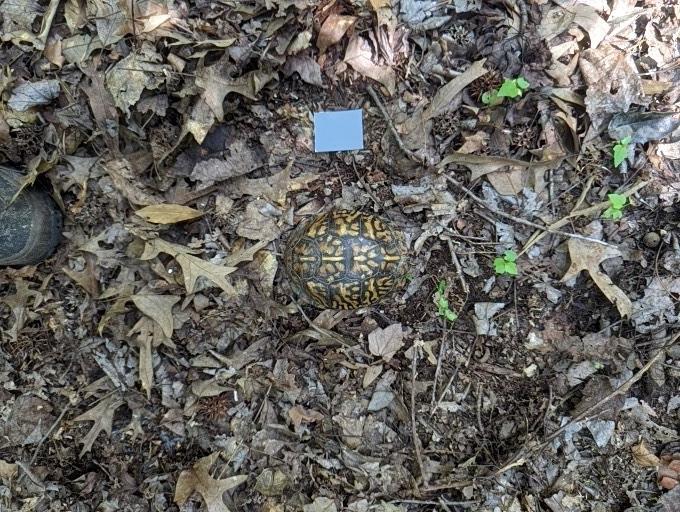

Supplement: Supplemental Information 6 — 98 photos of 98 turtles (single photo, all top view) that were used for the Citizen Science classification analysis. [file peerj-13-19690-s006.zip › TurtleClassification/TURTLE1094.jpg]

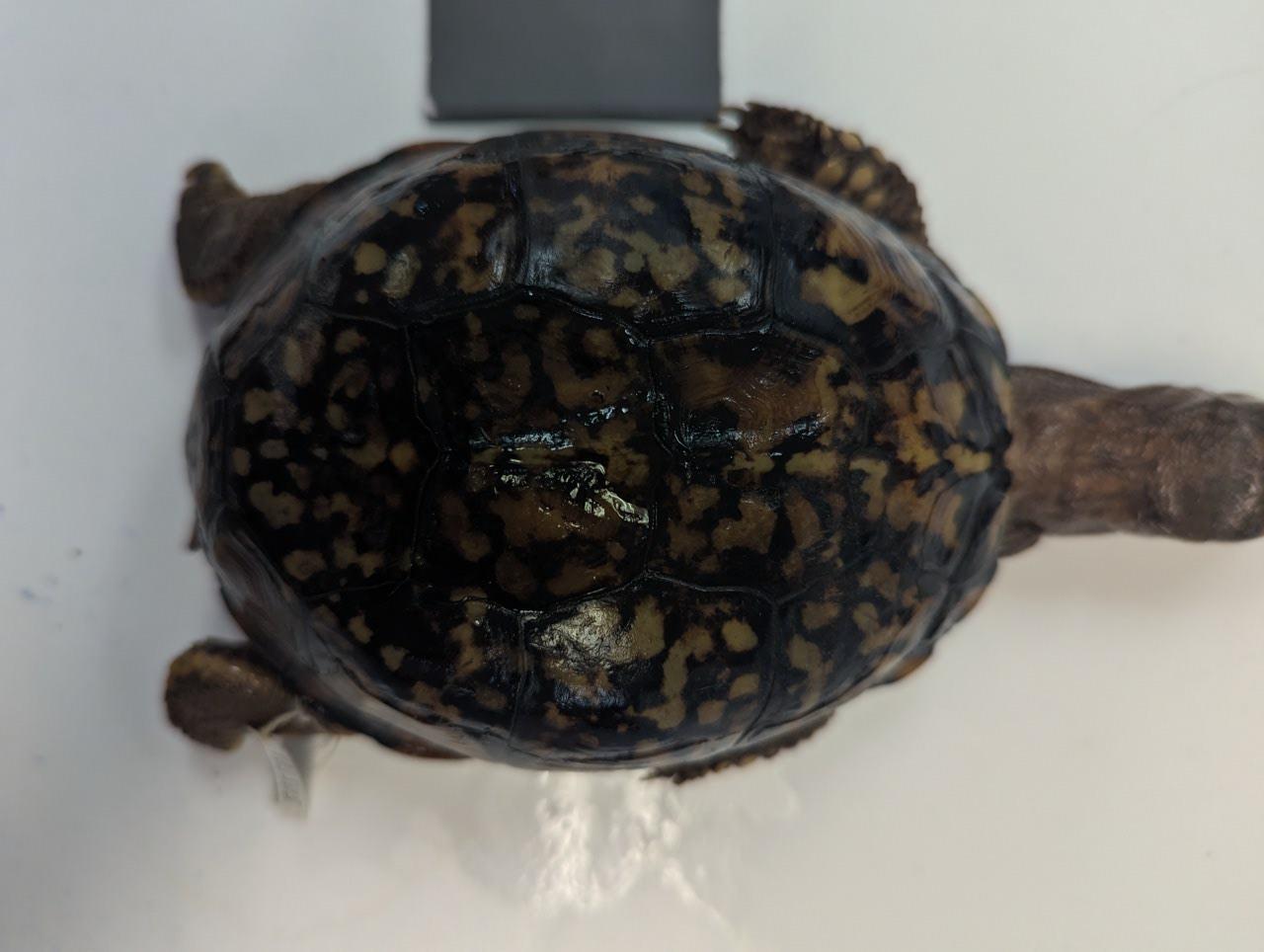

Supplement: Supplemental Information 6 — 98 photos of 98 turtles (single photo, all top view) that were used for the Citizen Science classification analysis. [file peerj-13-19690-s006.zip › TurtleClassification/TURTLE1095.jpg]

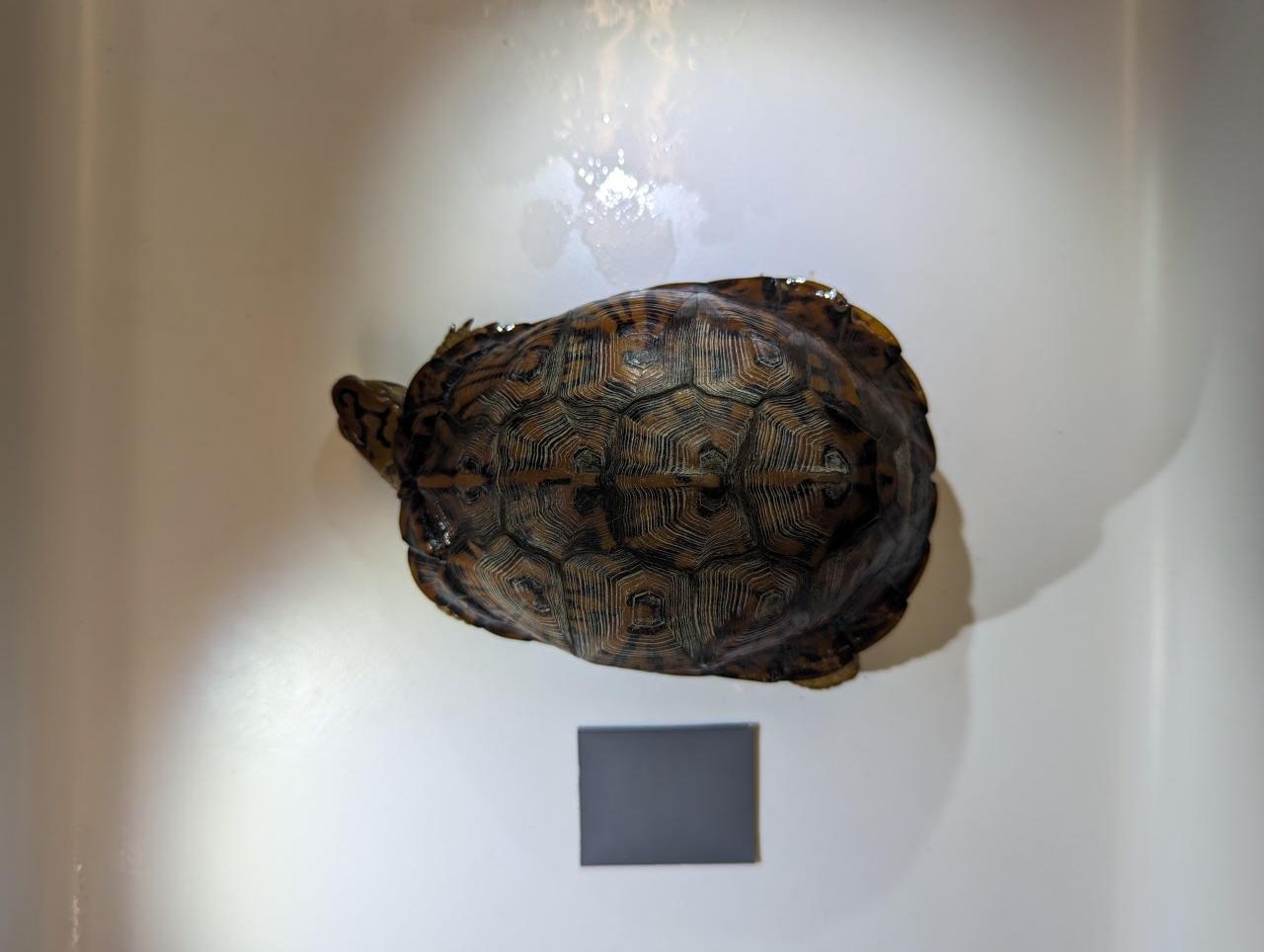

Supplement: Supplemental Information 6 — 98 photos of 98 turtles (single photo, all top view) that were used for the Citizen Science classification analysis. [file peerj-13-19690-s006.zip › TurtleClassification/TURTLE1096.jpg]

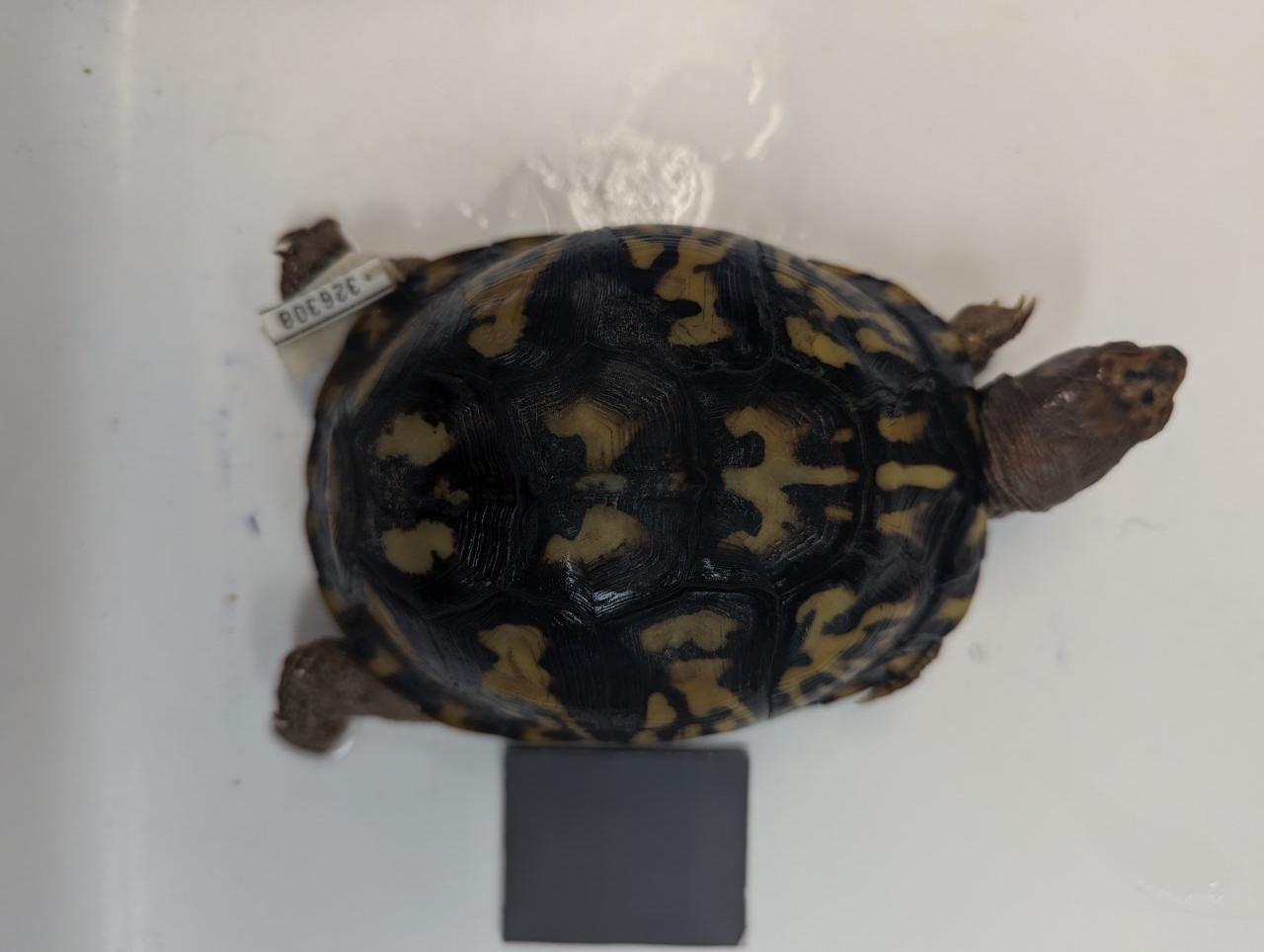

Supplement: Supplemental Information 6 — 98 photos of 98 turtles (single photo, all top view) that were used for the Citizen Science classification analysis. [file peerj-13-19690-s006.zip › TurtleClassification/TURTLE1097.jpg]

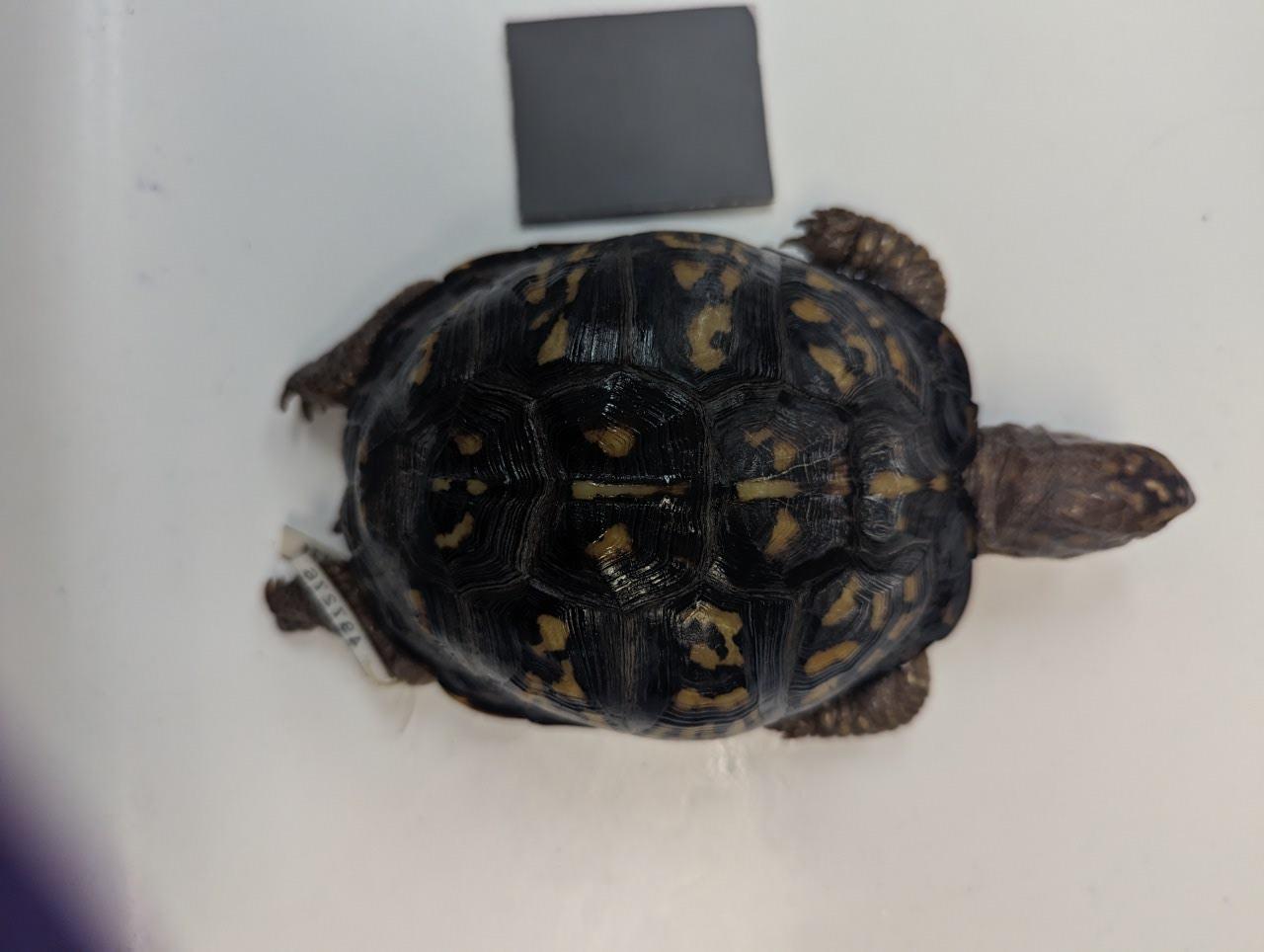

Supplement: Supplemental Information 6 — 98 photos of 98 turtles (single photo, all top view) that were used for the Citizen Science classification analysis. [file peerj-13-19690-s006.zip › TurtleClassification/TURTLE1098.jpg]
